# Oxygen isotopic evidence that Gale crater, Mars, was home to an Early Hesperian water reservoir that underwent significant evaporation

**DOI:** 10.1073/pnas.2511627122

**Published:** 2025-10-20

**Authors:** Amy E. Hofmann, P. Douglas Archer, Amy C. McAdam, Brad Sutter, Thomas F. Bristow, John M. Eiler, Christopher R. Webster, Gregory J. Flesch, Abigail A. Fraeman, Heather B. Franz, Christopher H. House, Elizabeth B. Rampe, Jennifer C. Stern, Paul R. Mahaffy, Charles A. Malespin, John P. Grotzinger, Ashwin R. Vasavada

**Affiliations:** ^a^Jet Propulsion Laboratory, California Institute of Technology, Pasadena, CA 91109; ^b^NASA Johnson Space Center, Houston, TX 77058; ^c^Jacobs Technology, Houston, TX 77258; ^d^NASA Goddard Space Flight Center, Greenbelt, MD 20771; ^e^NASA Ames Research Center, Mountain View, CA 94043; ^f^Division of Geological and Planetary Sciences, California Institute of Technology, Pasadena, CA 91125; ^g^Department of Geosciences, Pennsylvania State University, University Park, PA 16802

**Keywords:** Mars, water, oxygen isotopes, Gale crater, Curiosity

## Abstract

Simultaneous measurements of HDO, H_2_^18^O, and H_2_^16^O in water evolved during pyrolysis of powdered rock samples acquired by the Curiosity rover within Gale crater’s clay-bearing units indicate extreme and variable heavy-isotope enrichments averaging ~4.5 times the D/H ratio and ~1.03 times the ^18^O/^16^O ratio of terrestrial seawater. These enrichments are recorded in water desorbed from mineral surfaces and evolved from poorly crystalline phases, hydrated salts, jarosite, and clays. All evolved waters are deuterium-enriched relative to common terrestrial waters, reflecting hydrogen loss to space. Because oxygen in structurally bound hydroxyl groups is least likely to exchange with other sources over geologic timescales, we focus on oxygen in water evolved during dehydroxylation of smectite clays. Several samples have ^18^O/^16^O ratios commensurate with precipitation from, or near-complete equilibration with, water moderately ^18^O-enriched relative to terrestrial meteoric waters—consistent with other evidence that Mars’s hydrosphere is basically like Earth’s in terms of oxygen isotopes. Unlike hydrogen, oxygen atmospheric escape did not lead to extreme ^18^O enrichments on Mars. Locally, however, most Gale smectites’ ^18^O/^16^O values require a pronounced ^18^O-enrichment of their parental waters. On Earth, the most extreme ^18^O enrichments in surface waters are found in closed basins having undergone significant evaporative loss into a low-humidity atmosphere, and the ^18^O/^16^O of authigenic clay minerals formed in these environs reflect those enrichments. A similar process acting on the hydrologic reservoir local to Gale at the time of clay formation and early diagenesis is a plausible explanation for the distinctive oxygen isotopic compositions of these clays.

## Significance

We present isotopic measurements of mineral-bound water from rocks sampled by the Curiosity rover. These hydrous minerals’ strong ^18^O enrichments stand apart from prior observations of ancient martian water reservoirs. Extreme deuterium enrichments record atmospheric hydrogen loss and reveal chemical weathering in early martian near-surface environments. The amplitude of ^18^O enrichment and its correlation with deuterium in related samples indicate formation in an ancient lacustrine setting that underwent extensive evaporation into a low-humidity atmosphere—an insight into the early martian hydrologic cycle that complements previous sedimentological, mineralogical, and stratigraphic evidence. These data provide the clearest view to date of a martian lake’s hydrology during a period when climate change, chemical weathering, and prebiotic chemistry were active on Mars.

The surface of Mars—most notably its basaltic Noachian crust—has been transformed in its mineralogy and chemistry via extensive interaction with near-surface water early in Mars’s history. Evidence for such interactions comes from remote sensing observations (e.g., refs. 1–4) as well as from previous landed missions (e.g., refs. 5–7), which suggest both global- and local-scale water–rock interactions, varying locally in physical and chemical conditions, processes, and durations over which they persisted specific to individual terrains and localities on Mars. Our understanding of Mars’s current and past climate, geological and geochemical evolution, and potential habitability draws heavily upon what we know about the martian hydrologic cycle that drove these water–rock interactions. The hydrogen and oxygen isotopic compositions of the waters of Mars—bounded by measurements of the atmosphere, ice, and water-derived minerals—offer constraints on these issues.

In August 2012, the Curiosity rover landed in the northern Aeolis Palus area of Gale crater, where remote-sensing observations of outcropping bedrock had documented hundreds of meters of ancient, flat-lying layered rocks varying from lower (older) clay-bearing to higher (younger) sulfate-bearing lithologies (8). Since landing, Curiosity has traversed sedimentary facies reflecting fluvial, deltaic, lacustrine, and aeolian environments (9–16). The rover’s analytical geochemistry instruments have examined scooped soils and drilled rock powders from those facies to search for organic carbon, characterize mineralogical diversity, and determine rock and soil chemical and isotopic compositions. Together, these data document that rocks formed in a variety of potentially habitable environments associated with the global climatic transition from a wetter to a drier Mars.

The rover’s Chemistry and Mineralogy (CheMin) instrument (17) has characterized the mineral makeup of Gale crater lithologies, while both the quadrupole mass spectrometer (QMS) and tunable laser spectrometer (TLS) of the rover’s Sample Analysis at Mars (SAM) instrument suite (18) have determined the compositions of volatiles thermally evolved from the sand and rock powders collected along Curiosity’s traverse. Whereas the QMS surveys the full suite of evolved volatiles, the TLS makes high-precision measurements of the abundances and isotopic compositions of CO_2_, H_2_O, and CH_4_, which have been used to infer recent and ancient interactions between the atmosphere and surface solids (19, 20), ancient atmospheric loss and lacustrine conditions in Gale crater (21, 22), and the possible provenance of various carbon-bearing compounds (20, 23, 24).

**Fig. 1.**
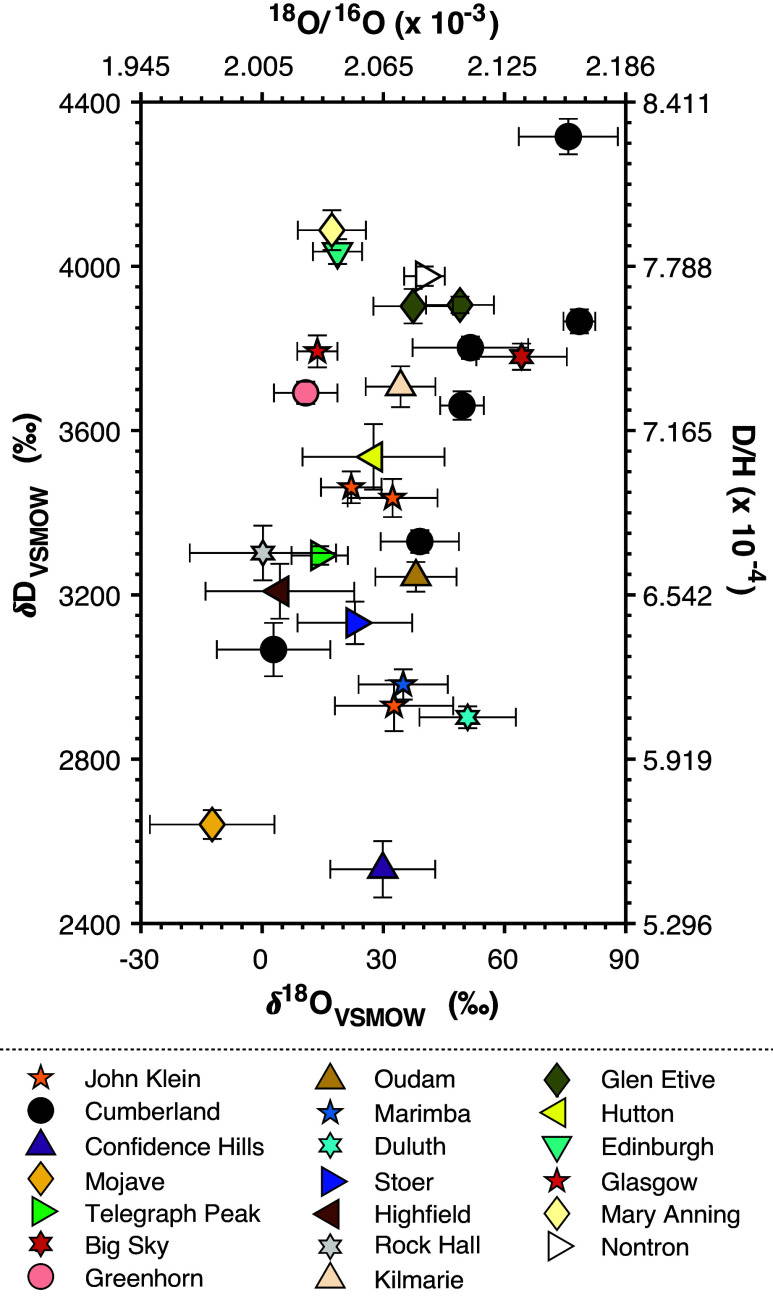
SAM-TLS evolved water isotopic compositions of drill powders sampled from multiple sites within Gale crater with outliers (RN2, GB1, QL) and modern aeolian scooped samples (RN3, RN4, GB2) removed. Error bars are two SEM. Delta notation is given with respect to the Vienna Standard Mean Ocean Water (VSMOW) terrestrial standard. See *SI Appendix* for a discussion of outliers and data culling and *SI Appendix*, Table S1 for figure data.

Here, we present the TLS dataset of paired hydrogen and oxygen isotopic compositions of water thermally evolved from scooped and powdered rock samples collected at Gale (Fig. 1 and *SI Appendix*, Table S1) and interpret these findings with reference to recognized processes of atmospheric escape from Mars and to processes known to control the isotopic compositions of lake waters on Earth. We find that the coupled hydrogen and oxygen isotopic compositions of evolved waters from authigenic minerals likely reflect precipitation from or extensive isotopic exchange with a local water reservoir (i.e., lake or near-surface pore waters) that became increasingly enriched in D and ^18^O. While D enrichments were primarily imparted to the local hydrologic reservoir due to secular loss of H to space (e.g., refs. 21 and 25), progressive evaporation—analogous to that observed in terrestrial closed basins that have undergone evaporative water loss into a low-humidity atmosphere—would have driven the local water reservoir at Gale to increasingly D- and, in particular, ^18^O-enriched compositions relative to the contemporaneous global reservoir values. Evidence for this process is particularly strong in the correlation between D- and ^18^O-enrichments observed in water evolved from clays in the Cumberland mudstone, which was heterogeneously infiltrated by early diagenetic fluids that themselves may have undergone progressive evaporation leading to the formation of syneresis cracks and cement precipitation (26).

## Tracing the Hydrologic Cycles of Earth and Mars with Hydrogen and Oxygen Isotopes

Earth’s oceans constitute the majority of the terrestrial hydrosphere; this planetary-scale reservoir has a near-uniform isotopic composition of approximately δD = 0 ± 5‰ and δ^18^O = 0 ± 1‰ relative to the terrestrial standard (VSMOW)^*^ (27). Most modern terrestrial meteoric waters have isotopic compositions that closely follow an empirical relationship known as the “meteoric water line”: δD = 8 × δ^18^O + 10 (28). Large variations in both the hydrogen and oxygen isotopic compositions of such waters are due to the physical processes responsible for evaporation, transport, and—most importantly—condensation of atmospheric water vapor. These processes drive the isotopic compositions of meteoritic waters to D- and ^18^O-depleted values reaching extremes of δD_VSMOW_ < –450‰ and δ^18^O_VSMOW_ < –55‰ (29). In contrast, terrestrial surface waters from hot springs, closed sedimentary basins, and shallow lakes in hot, windy, or arid areas experience pronounced evaporative losses of D- and ^18^O-depleted vapor, which drives the isotopic composition of residual waters to elevated δD and δ^18^O values. For example, waters sampled from rivers and lakes in East Africa show both D and ^18^O enrichments relative to VSMOW [e.g., δD_VSMOW_ of + 40‰, δ^18^O_VSMOW_ of +6‰ (28)]. Evaporation into a colder, drier climate such as that predicted for ancient Mars would lead to even greater D and ^18^O enrichments due to the increasing magnitude of isotopic fractionation between liquid and vapor-phase water with decreasing temperature and lower relative humidity [(30); *SI Appendix*, Table S6 and Fig. S14].

**Fig. 2.**
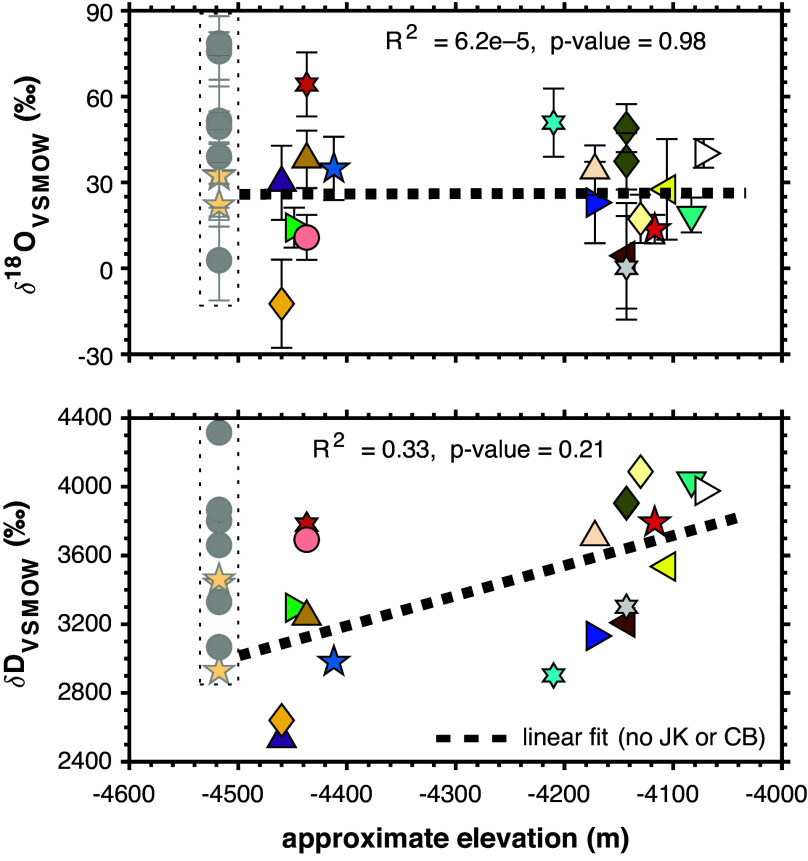
The oxygen isotopic compositions of evolved water appear uncorrelated with stratigraphic position, suggesting the presence of a local water reservoir at Gale that was already ^18^O-enriched prior to mineral precipitation. In contrast, the hydrogen isotopic compositions show a weak correlation up-section, reflecting the imprint of atmospheric evolution (i.e., secular H loss) on martian hydrologic reservoirs. Sample locations are shown within their stratigraphic context in *SI Appendix*, Fig. S2. Yellowknife Bay (YKB) samples (John Klein and Cumberland) are excluded from regressions due to closed system diagenetic alteration observed only in the YKB mudstone samples (40). Linear regressions (thick dotted black lines) are weighted to errors in the isotope values only; corresponding R^2^ and *P*-values are given at the top of each panel. Error bars in δ^18^O_VSMOW_ are two SEM; in δD_VSMOW_, they are smaller than the symbol size. Symbols are the same as in Fig. 1.

Although Mars had significant volumes of liquid water across its surface during the pre-Noachian (4.5 to 4.1 Ga) and Noachian (4.1 to 3.7 Ga) periods, surface waters in the Early Hesperian were likely limited to transient reservoirs (e.g., refs. 31 and 32). Several lines of evidence—particularly the hydrogen isotopic compositions of modern martian atmospheric water vapor (δD_VSMOW_ values of approximately +3,000 to +6,000‰; e.g., refs. 33–35) as well as authigenic (δD_VSMOW_ of ~ +2000‰; e.g., ref. 36) and other hydroxyl-bearing (δD_VSMOW_ of ~ +1,000 to +3,000‰; e.g., ref. 37) minerals in martian meteorites—indicate that atmospheric loss of hydrogen to space was a major driver of Mars’s desiccation and that much of this loss occurred early in Mars’s history. This global D-enriched isotopic signature of the remaining water (reaching δD_VSMOW_ values of +1,000s of per mille) is shared by many hydrogen-bearing martian materials, including those sampled at Gale (Figs. 1 and 2 and ref. 21), and contrasts starkly with the relatively modestly D-depleted isotopic signatures of terrestrial hydrogen in authigenic and other hydroxyl-bearing minerals [e.g., δD_VSMOW_ values of order –80 to –150‰, (38, 39)]. Conversely, all martian materials—apart from the Gale crater authigenic minerals sampled and analyzed by Curiosity—are broadly similar in δ^18^O to their terrestrial equivalents (*SI Appendix*, *Supplementary Discussion* and Fig. S1). This finding is consistent with 1) the relatively modest oxygen isotopic fractionations associated with atmospheric escape and 2) buffering of the oxygen isotopic compositions of residual waters via interactions and exchange with oxygen in the far more abundant rocks of the crust (and possibly deeper lithosphere), particularly early in Mars’s history, and later by exchange with atmospheric CO_2_. In this context, the exceptional ^18^O enrichments of clay-associated water analyzed by the TLS provide evidence for a distinctive local history of martian water in and around Gale crater during the Early Hesperian.

## Interpreting the Isotopic Composition of Water Evolved from Clay Minerals

Terrestrial clay minerals form via weathering reactions and direct precipitation from aqueous solutions (41). In both cases, the D/H and ^18^O/^16^O ratios of the clay hydroxyl groups reflect the physical and chemical conditions under which the minerals formed and serve as a proxy for the isotopic composition of the parent water, thereby providing information on paleoenvironments (39). Studies of terrestrial clays from marine environments indicate that their hydrogen and oxygen isotopic compositions can be retained over geologic timescales up to millions of years in grain size fractions >0.1 micron as long as temperatures remain below ~20 °C (42–47). Similar conclusions have been reached based on studies of clay minerals from terrestrial weathering environments (e.g., refs. 48–50) and diagenetically altered sandstones (e.g., refs. 51–53). Oxygen isotopes appear to exchange less readily than hydrogen isotopes and thus may serve as a more robust indicator of conditions at the time of clay mineral formation (54, 55). Importantly, the δ^18^O values of these terrestrial clays correspond to the weighted average of all structural oxygen within the clay minerals (i.e., hydroxyl and Si- or Al-bound O), whereas hydrogen resides in the hydroxyl groups only.

**Fig. 3.**
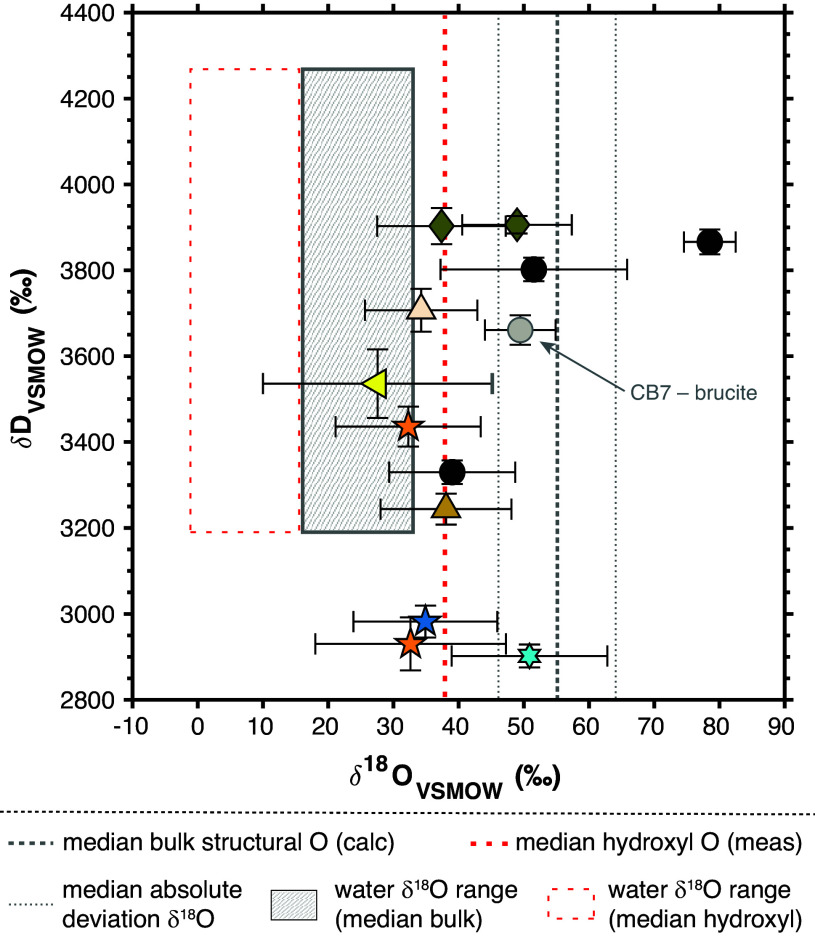
Hydroxyl water evolved during high-temperature smectite dehydroxylation and measured by the TLS (symbols) has a median δ^18^O_VSMOW_ value of 38‰ (red vertical dashed line). The gray vertical dashed line corresponds to the median bulk structural oxygen δ^18^O for the Gale smectites calculated based on the median measured hydroxyl δ^18^O assuming mass balance and an intracrystalline ^18^α_tetO-OH_ value of 1.02. Gray vertical dotted lines denote the adjusted median absolute deviation (MAD) of the bulk structural oxygen values and thus bound the range of bulk structural δ^18^O values for the Gale smectites. The hashed gray rectangle represents the range of isotopic compositions for a liquid water reservoir in isotopic equilibrium at 0 °C with smectite having bulk structural δ^18^O_VSMOW_ values from approximately 46 to 63‰ (i.e., median ± MAD, between gray dotted lines) and δD_VSMOW_ values between approximately 2,900‰ (DU) and 3,900‰ (GE). Given for direct reference to the datapoints, the red dotted rectangle corresponds to the same calculated using the smectite median ± MAD measured hydroxyl δ^18^O_VSMOW_ values. CB7 (gray circle) includes contributions from interlayer brucite dehydroxylation. All other symbols are the same as in Fig. 1.

Paired hydroxyl hydrogen and structural oxygen isotopic compositions of terrestrial smectites span a range of δD_VSMOW_ values from approximately –145 to –5‰ and δ^18^O_VSMOW_ values of +5 to +28‰ (39, 46, 48, 56–59). In comparison, the δD_VSMOW_ values of hydroxyl water from Gale crater clay minerals span a range from approximately +2,900 to +3,900‰, with the corresponding hydroxyl δ^18^O_VSMOW_ values clustering between approximately +30 and +50‰ (median +38‰; Fig. 3). The Gale smectite values reflect only water released by high-temperature heating experiments and are interpreted to derive almost entirely from clay dehydroxylation with little to no contributions from other minerals (e.g., hydrated salts or adsorbed water; *SI Appendix*, Table S1) or from tetrahedrally coordinated oxygen within the clay mineral lattice. Based on studies of intracrystalline oxygen isotopic fractionation in various clay minerals, hydroxyl oxygen may be approximately 10 to 30‰ lower in δ^18^O than the tetrahedrally coordinated Si- and Al-bound oxygen for clay minerals equilibrated near 20 °C (60–62). Terrestrial clay δ^18^O values reported in the literature correspond to a weighted average of the oxygen isotopic compositions of these two structural sites. For direct comparison between the martian clays and their terrestrial counterparts, we use mass balance constraints to calculate the Gale smectites’ corresponding structural oxygen isotopic compositions assuming isotopic equilibrium between the tetrahedrally and octahedrally coordinated oxygens, a median intracrystalline isotopic fractionation of 20‰ (i.e., ^18^α_tetO-OH_ = 1.02), and no kinetic isotope effects associated with dehydroxylation (60–62). Based on this calculation, the structural oxygen isotopic compositions of Gale authigenic clay minerals should be of order +50 to +70‰ in δ^18^O_VSMOW_.

Sedimentological, mineralogical, and elemental data from Curiosity’s instrument suite coupled with geochemical modeling suggest that Gale crater clays formed in situ, likely via low-temperature aqueous alteration of basaltic material during or near the time of sediment deposition (63–66). Clay minerals preferentially incorporate H (the lighter isotope) over D and ^18^O (the heavy isotope) over ^16^O into their structures during isotopic exchange with water, leading to mineral-water differences of approximately – 70‰ in δD and +30‰ in δ^18^O at temperatures from 0 to 10 °C (67, 68). Thus, a circumneutral pH water reservoir in isotopic equilibrium with the Gale clay minerals at 0 °C would need to have had a δD_VSMOW_ value between +3,000 and +4,000‰ and a δ^18^O_VSMOW_ value between +20 and +40‰ based on the smectites’ structural oxygen isotopic compositions calculated above and shown in Fig. 3. This δD range is consistent with the local hydrosphere bearing the global isotopic imprint of atmospheric hydrogen loss, but the δ^18^O range is significantly more ^18^O-enriched relative to previous estimates of martian water reservoirs and higher than expected for surface and near-surface water buffered by exchange with both CO_2_ in the martian atmosphere and rocks in the martian lithosphere (*SI Appendix*, Fig. S1). Thus, the authigenic clay minerals analyzed by the TLS must have formed from or isotopically exchanged with a local water reservoir at Gale that was already enriched in ^18^O relative to most of the martian hydrosphere (i.e., by tens of per mille) and that persisted throughout the period of authigenic clay mineral formation (Fig. 2).

## Implications for Local Hydrology at Gale

The δ^18^O_VSMOW_ of near-surface, modern atmospheric CO_2_ on Mars, as measured in situ by the TLS on Curiosity [48 ± 5‰, 2 SE; (69)] is moderately ^18^O-enriched compared to that of modern terrestrial tropospheric CO_2_ [δ^18^O_VSMOW_ of approximately 39 to 42‰ (70, 71)]. There are no measurements of ancient martian atmospheric CO_2_, either via mineralogical proxies or fluid inclusions in martian meteorites, and numerical simulations of martian atmospheric evolution over geologic time model the carbon—but typically not the oxygen—isotopic composition of CO_2_ (e.g., ref. 72). Carbon dioxide released into the early martian atmosphere via magmatic degassing would have most likely been in isotopic equilibrium with other dissolved volatiles, including H_2_O, and silicate igneous minerals at the high temperatures of basaltic melt. Igneous minerals in martian meteorites have terrestrial-like oxygen isotopic compositions, i.e., δ^18^O_VSMOW_ mean values of +4.5‰ (e.g., ref. 73; *SI Appendix*, *Supplementary Discussion* and Fig. S1). Thus, the degassed CO_2_ initially would have had a δ^18^O_VSMOW_ value close to that of the rocks, but—as described in the paragraphs that follow—subsequent weathering reactions among CO_2_, H_2_O, and the lithosphere on earliest Mars would have driven martian CO_2_ toward a more terrestrial-CO_2_-like oxygen isotopic composition.

On Earth, the oxygen isotopic composition of atmospheric CO_2_ is buffered via isotopic exchange with seawater, which is itself buffered over geologic timescales by isotopic exchange with crustal rocks through weathering reactions and hydrothermal processes. There is no strong case for long-lived global oceans on Mars, yet it has possessed a surface and near-surface hydrosphere for much of its history, and—based on constraints from martian meteorite and surface records—aqueous weathering reactions likely occurred on Mars throughout its earliest geologic epochs. Despite the higher-than-terrestrial *p*CO_2_ conditions during the geologic period(s) when liquid water would have been stable on the martian surface, the oxygen isotopic composition of martian atmospheric CO_2_ suggests it too is buffered by that of rocks in the martian crust—and has been for much of Mars’s history. Any fractionation due to ^16^O escape to space would have led to increasingly ^18^O-enriched CO_2_, but buffering by the large nonatmospheric oxygen–bearing reservoirs on Mars would be expected to dilute the magnitude of such an effect (74).

**Fig. 4.**
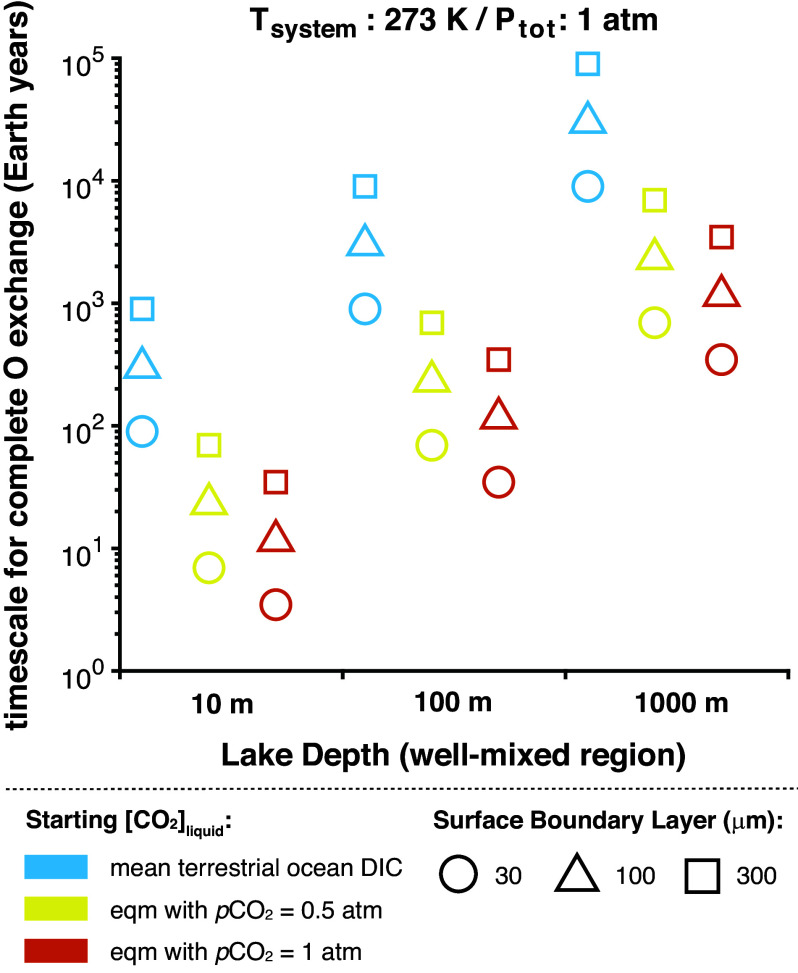
Timescales in Earth years for complete oxygen exchange between H_2_O in a hypothetical lake of constant depth at Gale and atmospheric CO_2_ calculated using the parameter space bounded by variables given in *SI Appendix*, Table S4. Colors correspond to the concentration of dissolved CO_2_, which was assigned to be either equal to mean terrestrial oceanic DIC (3 mmol/L, blue symbols) or the concentration in equilibrium with a CO_2_ partial pressure of either 0.5 atm (yellow symbols) or 1 atm (orange symbols). Calculations were performed for a freshwater system. The thickness of the boundary layer (i.e., the stagnant film at the water surface) was either 30, 100, or 300 microns (circles, triangles, or squares, respectively). The well-mixed region of the water column—taken as a proxy for lake depth—is given on the x-axis. Model details and additional plots can be found in *SI Appendix*, *Supplementary Methods* and Fig. S9.

On early Mars, atmospheric CO_2_, liquid water, and crustal rocks in mutual contact would have undergone the same collective reactions, i.e., dissolved inorganic carbon (DIC) chemistry and the silicate weathering cycle, that lead to quasi-equilibrium among the oxygen isotopic compositions of their terrestrial counterparts. Liquid water in oxygen isotopic equilibrium with atmospheric carbon dioxide should be approximately 45‰ lighter in ^18^O than gas-phase CO_2_ at 0 °C (75). Gale lake waters in isotopic equilibrium with ancient martian CO_2_ (having δ^18^O_VSMOW_ values of approximately +40 to +50‰ based on the preceding arguments) would thus have oxygen isotopic compositions of order –5 to + 5‰—in other words, terrestrial-like. As noted in the preceding section, structural oxygen isotopic compositions estimated from the Gale smectites’ hydroxyl δ^18^O_VSMOW_ values necessitate equilibration with waters having δ^18^O_VSMOW_ values of approximately + 20 to +40‰. If, however, only the hydroxyl oxygens equilibrated with the fluid, then the water would need to have been somewhat less ^18^O-enriched (Fig. 3). In either case, only Gale smectites with the least-enriched oxygen isotopic compositions (i.e., possibly Hutton; Fig. 3) could have isotopically equilibrated with a body of water itself in isotopic equilibrium with martian atmospheric CO_2_. Given near-surface atmospheric *p*CO_2_ of ~10 to 100 s of mbar during the Early Hesperian when the Gale clay-bearing sediments were being deposited (72, 76, 77) and surface temperatures near the freezing point of pure water (e.g., ref. 78), such a scenario may have been possible, albeit only for the smallest water reservoirs (Fig. 4)—i.e., those much less voluminous than the deepest lakes at Gale (79)—or for the upper regions of stratified lakes.

The simplest way to achieve parental waters with ^18^O-enriched compositions necessitated by the Gale smectites is via evaporative water loss, a process well-documented in terrestrial lakes (80). For evaporation to drive isotopic enrichment, the lake [or near-surface (81)] water must evaporate faster than the CO_2_-water equilibration timescale. As summarized in ref. 12, standing bodies of water in Gale crater during the time of sediment deposition were likely stable against net evaporation for hundreds to thousands to tens of thousands of years. Such persistence times are comparable to those required for complete oxygen isotope exchange between CO_2_ in the martian atmosphere and H_2_O in a well-mixed hypothetical freshwater lake ~100 to 1,000 m deep under a *p*CO_2_ of 0.5 atm (~500 mbar) and temperatures near freezing (Fig. 4; see *SI Appendix*, *Supplementary Methods*, Fig. S9, and Table S4 for modeling details). And yet, the Gale smectites’ oxygen isotopic compositions are too ^18^O-enriched to be consistent with such a scenario.

A 300-m-deep lake at Gale would have to evaporate at a rate faster than 300 mm/y to prevent the lake water from isotopically equilibrating with atmospheric CO_2_ at the ~1,000-y e-folding timescale. Long-term mean evaporation rates measured for modern terrestrial lakes in cold, arid to semiarid climates (82) range from ~300 to 1,100 mm/y (83). At equivalent temperatures and relative humidities, the lower total near-surface atmospheric pressures on Mars would facilitate comparable to slightly faster evaporation rates (e.g., ref. 84). It is thus plausible that the Gale smectites’ δ^18^O values reflect isotopic exchange with water that was evaporatively enriched in ^18^O while it was ponded in the crater itself. Indeed, the many lines of geologic evidence indicating long-lived lake stands at Gale do not preclude episodic, short-term, partial dry-down and refilling, from, for example, the rise of an interconnected groundwater table (85).

## Local Evaporative Conditions at Gale and the Case of the Cumberland Mudstone

Additional support for our evaporative enrichment hypothesis comes from the suite of paired hydrogen and oxygen isotope data for water evolved from six separate subsamples of the drilled Cumberland mudstone (CB; *SI Appendix*, Table S1). The Cumberland drill site is situated within the Yellowknife Bay formation’s stratigraphically lowest unit, the >1.5 m-thick Sheepbed member mudstone (*SI Appendix*, Fig. S2). This mudstone is inferred to have been deposited subaqueously, possibly via suspension settling, in an ancient freshwater lake at the distal end of an alluvial fan (11, 86, 87). Major element chemistry reflects a broadly basaltic composition consistent with average martian crust, while the chemical index of alteration indicates that the sediments experienced negligible chemical weathering prior to deposition, possibly reflecting cold, arid conditions (87). Based on Sheepbed mudstone mineralogy (86) as well as the clay mineral compositions and geochemical mass balance constraints (64), Cumberland smectites likely formed in situ via aqueous alteration of olivine soon after sediment deposition. Thermochemical (63) and thermodynamic (64) modeling results support inferences that the observed mineral assemblage could have formed via reactions between the Fe-rich sedimentary rocks and a CO_2_-poor, dilute, circumneutral-to-alkaline aqueous solution at clement surface conditions and that aqueous fluids may have persisted for thousands to hundreds of thousands of years. The 13.5 Å interlayer spacing measured in Cumberland smectite (86) most likely reflects partial intercalation by Mg-hydroxyl groups driven by ingress of alkaline Mg-rich waters—possibly the parental fluid from which the Cumberland raised ridges’ Mg-silicate cements ultimately precipitated—during early diagenesis (26, 40, 64). This partial chloritization of smectite in Yellowknife Bay was heterogeneous: the John Klein mudstone, which is located ~2.75 m away from and within ~30 cm stratigraphically of Cumberland, had less partially chloritized smectite than Cumberland (40). Notably, partial chloritization is not observed in any other Gale minerals, indicative of variable early diagenetic alteration over small spatial scales at Gale.

**Fig. 5.**
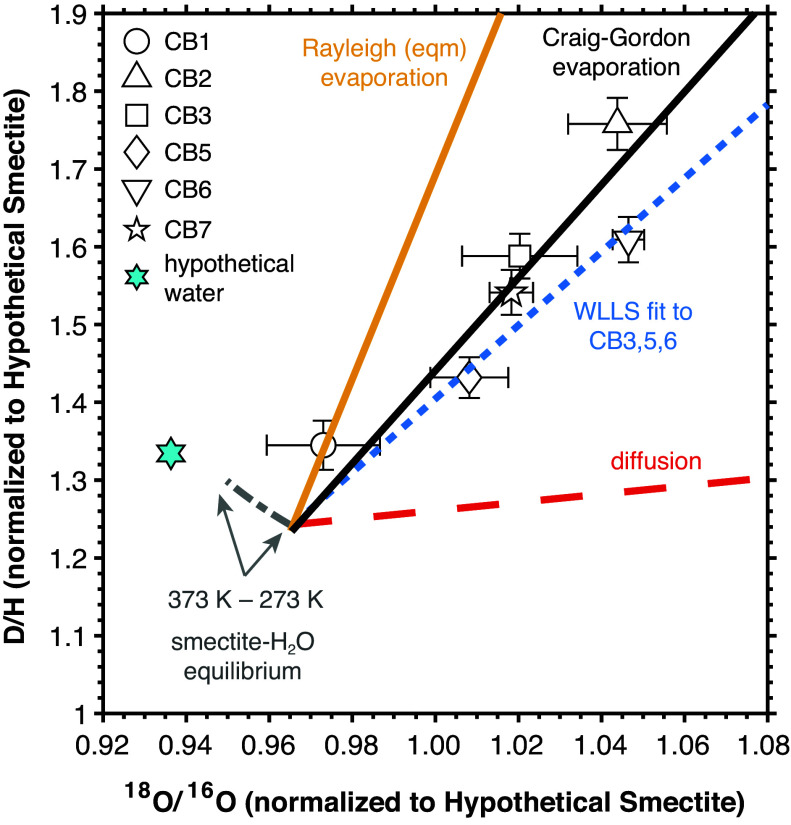
Schematic comparison of the Cumberland data (open symbols) to diagnostic slopes of isotopic fractionation driven by interdiffusion of molecular water through a He carrier gas (dashed red line), smectite-water equilibrium isotope exchange for temperatures of 373 to 273 K (dotted-dashed gray line), and the isotopic composition of smectite hydroxyl groups isotopically equilibrated with a body of water at 273 K undergoing either classic Rayleigh evaporation (an equilibrium phenomenon; gold line) or Craig-Gordon evaporation (a modified Rayleigh process; black line). All data have been normalized to the D/H and ^18^O/^16^O values of a “Hypothetical Smectite” described in *SI Appendix*, Supplementary Methods. Smectite compositions were calculated using the smectite ^D^α_OH-H2O_ and ^18^α_bulkO-H2O_ values given in *SI Appendix*, Table S5 assuming isotopic equilibrium with a hypothetical freshwater reservoir that had an initial isotopic composition corresponding to the cyan hexagon. Craig-Gordon evaporation was modeled for a relative humidity (*p*_H2O_/*p*_satH2O,273K_) of 20%. The dotted blue regression line is a weighted (in both *x* and *y*) linear least squares fit (WLLS) to the normalized Cumberland high-temperature smectite dehydroxylation data (CB3, CB5, CB6). Error bars are two SEM. The hypothetical water composition was chosen for illustrative purposes such that all the “processes” lines would be anchored to the same location in normalized ratio space. See *SI Appendix*, *Supplementary Methods* and Fig. S12 for detailed information.

The paired hydrogen and oxygen isotopic compositions of water released from the six Cumberland subsamples plot along a linear array in D/H versus ^18^O/^16^O (Fig. 5; see also *SI Appendix*, Figs. S10–S12). The most widely known linear relationship between D/H and ^18^O/^16^O ratios in water is the terrestrial meteoric water line, the slope of which (~8) can largely be explained by Rayleigh distillation driven by progressive condensation from an atmospheric air mass. In addition to Rayleigh-type processes, two-component mixing, diffusive fractionation, and equilibrium isotopic exchange between minerals and liquid water are also characterized by distinct slopes in normalized isotope ratio space. In Fig. 5, we compare the slope of the Cumberland data array to diagnostic slopes of isotopic fractionation driven by interdiffusion of molecular water through a He carrier gas (as might occur within the SAM instrument), smectite-water equilibrium isotope exchange, and the isotopic composition of smectite isotopically equilibrated with a body of freshwater undergoing either classic Rayleigh evaporation (an equilibrium phenomenon) or Craig-Gordon evaporation (a modified Rayleigh process). Additional and more detailed figures, descriptions of the numerical models, and associated equations and constants can be found in *SI Appendix*, *Supplementary Methods*.

The sources of Cumberland (CB) evolved water include decomposition of hydrated salts (CB1, CB2), dehydroxylation of smectite clays (CB3, CB5, CB6), and dehydroxylation of “brucite-like” interlayers in partially chloritized smectite (CB7). If all six Cumberland subsamples share a common paragenesis, then the hydrogen and oxygen isotopic compositions of their mineral-evolved waters would reflect an evolution in the isotopic composition of the local water reservoir during mineral formation. If the six subsamples come from two or more paragenetic sequences, then the linear array in Fig. 5 could instead reflect mixing between mineral-bound water of diverse origins. All six subsamples fall along a common regression line having a slope within one SE of that for the smectite-only regression (*SI Appendix*, Fig. S10) and together pass a collinearity permutation test with a *P*-value of ~0.034 (*SI Appendix*, *Supplementary Methods*). It is thus plausible that the mineralogical sources of evolved water from all six subsamples are paragenetically related. The mineralogical and geochemical self-similarity of the Cumberland smectites and their dissimilarity to all other clay minerals analyzed by Curiosity strongly suggests that the CB smectites are related, regardless of their relationship to the hydrated salts. For consistency with the rest of the manuscript, we focus on the smectite subpopulation in the discussion that follows.

If the Cumberland smectites isotopically equilibrated with Gale waters while the lake was evaporating and/or with variably enriched infiltrating fluids (that themselves were likely undergoing evaporation) during early diagenesis, then the isotopic compositions of the clay hydroxyl waters would be passively tracking the changing isotopic composition of the local hydrologic reservoir. As shown in Fig. 5, the slope of a weighted linear least squares fit (WLLS blue dotted line) to the high-temperature smectite-evolved water subsamples (CB3, CB5, CB6) most closely aligns with that defined by Craig-Gordon evaporation (black line). Unlike classic Rayleigh evaporation (gold line), which is parameterized in terms of instantaneous thermodynamic equilibrium between vapor and liquid phases (88), the Craig-Gordon model explicitly accounts for kinetic effects—such as diffusive fractionation through a boundary layer between the surface of a lake and the atmosphere—and atmospheric relative humidity (27). Inclusion of these parameters has been shown to better capture observed isotopic fractionations associated with the terrestrial hydrologic cycle because evaporation is not occurring under conditions of thermodynamic equilibrium at the liquid–vapor interface (e.g., ref. 89 and references therein). The Craig-Gordon model calculation used to generate results in Fig. 5 tracks the isotopic fractionation associated with progressive evaporation of a hypothetical Gale lake into a water-vapor-undersaturated atmosphere with a relative humidity of 20% (90) and a constant isotopic composition at 273 K. Although authigenic terrestrial Mg-smectites are often found within shallow, closed-basin lakes undergoing evaporation—as recorded in the smectites’ hydroxyl hydrogen and structural oxygen isotopic compositions (91, 92)—hypersalinity is not required for initiating chloritization. The latter is significant given the absence of evaporative salts in the Cumberland mineral assemblage (86), which negates a complete or nearly complete dry-down of the Gale paleolake and is consistent with only partial chloritization of the Cumberland smectites.

The range in paired isotopic compositions measured in the Cumberland dataset (Fig. 5) suggests that the smectites are likely mixtures of clay populations that underwent different extents of isotopic exchange with an evolving aqueous fluid during their formation and/or with younger, increasingly D- and ^18^O-enriched aqueous solutions that infiltrated the sediments during early diagenesis. In terrestrial settings, a single sediment sample can contain mixtures of multiple clay fractions that exhibit different chemistries and oxygen isotopic compositions associated with crystallite size as well as degree and mode of alteration (e.g., refs. 43, 46, and 62). The Cumberland sample is an aggregation of material drilled over a few centimeters’ depth of a natural rock target with observed (e.g., nodules, a minor vein) and microscopic heterogeneity. While mixing is expected to have occurred during sample transfer and processing (e.g., sieving) of the material, aliquots may retain some heterogeneity.

## Conclusions

The most parsimonious interpretation of the TLS EGA smectite dataset is that these clay minerals were passively recording the isotopic composition of waters at Gale that likely began with a terrestrial-like oxygen isotopic composition—consistent with other similarities in the ^18^O/^16^O oxygen isotope systematics of Earth and Mars—but was subsequently enriched in ^18^O through progressive evaporation, possibly mediated by groundwater replenishment, into a water-undersaturated, CO_2_-rich atmosphere during the Early Hesperian. Further support for this hypothesis comes from the observed linear relationship between the paired hydrogen and oxygen isotopic compositions of hydroxyl waters evolved from Cumberland clay minerals. These clay minerals are compositionally unlike all others seen at Gale, likely reflecting differences in local physicochemical conditions and, thus, the authigenic (and diagenetic) pathways during and soon after in situ clay mineral formation. It is also likely that later-stage diagenetic fluids pervading the rocks were themselves ^18^O-enriched, as suggested by the highly enriched carbon and oxygen isotopic compositions of CO_2_ derived from iron-rich carbonates in sediments stratigraphically spanning the climatic transition from a wetter (clay-rich) to a drier (sulfate-rich) Mars (22). All mineral-evolved waters studied here also bear the imprint of deuterium enrichment imparted to the martian global hydrosphere due to early and ongoing loss of hydrogen to space.

Although NASA’s Perseverance rover will not be caching rocks from Gale crater, the collection by Perseverance and delivery to Earth of ancient martian sedimentary rocks from Jezero crater by Mars Sample Return would enable petrographically contextualized, high-precision hydrogen and oxygen isotopic measurements of individual hydrated mineral phases under controlled laboratory conditions. Those data from martian clay minerals would provide more robust, comprehensive constraints on the hydrology of the lake system at Jezero—a place not unlike Gale, where standing bodies of water persisted for long periods of time, albeit interleaved with intermittent dry periods as Mars lost its water to space, the ice caps, deep subsurface reservoirs, and crustal hydration.

## Methods

### Evolved Gas Analysis Experiments with SAM.

Evolved gas analysis (EGA) was used to determine the identity and quantity of volatiles produced during desorption from, dehydration of, or thermal decomposition of materials. Because these processes are thermodynamically and kinetically driven, the volatile source (e.g., smectite) and associated reactions (e.g., dehydroxylation) can be determined based on the volatile release pattern as a function of temperature. In a typical SAM EGA experiment, the evolved gas stream is briefly diverted into the TLS over a predetermined temperature interval to measure the carbon, oxygen, and hydrogen isotopic compositions and concentrations of CO_2_, H_2_O, and CH_4_. The QMS continually receives some or all the evolved gas stream throughout the experiment.

Prior to receipt of the solid sample, both the SAM gas transfer lines and manifold are heated to 135 °C for 150 min and the receiving quartz cup is heated to ~900 °C for 5 min while pumping to drive off adsorbed gases and minimize background signal. Transfer lines then remain at 135 °C for the duration of the experiment to facilitate volatile transfer. A few sols later, SAM receives via its Sample Manipulation System (SMS) a scooped or drilled sample typically sieved to ≤150 μm from Curiosity’s Sample Acquisition Sample Processing and Handling system (SASPaH) for all samples up to Quela (sol 1722). A drill feed mechanism failure caused all samples after Quela to be delivered to SAM using the feed-extended sample transfer (FEST) protocol. The FEST procedure deposited drilled sample into SAM by placing the drill over the SAM inlet and then reversing the drill to permit the fines to fall into the SAM sample cup (93). The sample is delivered to the preconditioned quartz cup, which is then sealed within one of the pyrolysis ovens and heated from ~40 °C (the ambient temperature inside SAM) up to ~890 °C at a ramp rate of ~35 °C per minute. Pure helium carrier gas (25 mbar in the SAM oven) was flushed at a flow rate of ~0.8 cm^3^ per minute (STP) through the porous frits of the quartz cup, carrying the evolved gases past the capillary inlet of the QMS for continuous mass spectrometric analysis. Gaseous species were identified by mass-to-charge ratio (*m/z*) of the molecular species itself or one of its isotopologues. The TLS gas analysis began by first evacuating the TLS sample (Herriott) cell to record empty cell spectra, and then the sample cell was opened to receive a “temperature cut” ingest (e.g., 200 to 350 °C). The cell was then sealed off for analysis. Empty cell water abundances were extremely low compared to the full cell EGA values (tens of parts-per-million by volume, ppmv) so that empty cell subtractions were not needed. TLS full cell pressures (mainly helium) were typically 5 to 15 mbar depending on the width of the temperature cut.

The TLS analyzed water thermally evolved from three of four John Klein subsamples (JK2, JK3, JK4) and six Cumberland subsamples (CB1, CB2, CB3, CB5, CB6, CB7). Subsamples JK2, CB1, CB2, CB3 were all run under the experimental conditions described in the preceding section. The CB5 subsample was delivered to a SAM cup that was initially preheated to 870 °C in the pyrolysis oven, allowed to cool, reheated to ~250 °C, and then moved outside the oven for sample receipt; these modifications were performed in order to limit contributions from adsorbed MTBSTFA and DMF vapor associated with other derivatization experiments (94). The cups used for John Klein subsamples JK3 and JK4 and Cumberland subsamples CB6 and CB7 were preheated to 250 °C and then held at that temperature for 27 min prior to starting the continuous ramp to 870 °C (95).

### Determining the Origins of Volatiles Generated During EGA Experiments.

The origins of gases detected by SAM (sample composition, mineralogy, etc.) were determined by comparison to databases of thermal analyses for natural or synthetic samples expected to be present on Mars. For example, decomposition of gypsum produces a distinctive release of low temperature (<200 °C) H_2_O (96). A release of CO_2_ at 400 °C would be consistent with an iron carbonate (22). The determinations are frequently nonunique because natural samples contain complex mixtures of species that, for example, might catalyze a decomposition reaction so that a mixture of two species would appear different in EGA than either species run alone. Thus, many lab runs of different analog materials are conducted, and data are compared across different samples, to best understand sample composition.

The mineralogical sources of evolved water in martian rock powders analyzed by SAM-TLS (*SI Appendix*, Table S1) are deduced from the overall mineralogy of an individual sample as determined by CheMin, the EGA water evolution curve, and the temperature cut over which gas is sent to the TLS (e.g., *SI Appendix*, Fig. S5). Over the lowest temperature ranges (i.e., <200 °C), the TLS is likely sampling residual water adsorbed to mineral surfaces as well as water evolved from multiple mineralogical sources, including gypsum as well as that of the so-called “X-ray amorphous component” (e.g., ref. 95). The exact nature of the amorphous material (or materials) remains uncertain, but it bears resemblances to basaltic glasses (97, 98), terrestrial allophane-containing palagonitic dust found in volcanic soils on Mauna Kea in Hawai’i (99), amorphous silica (100), ferrihydrite (101), and to the nanophase ferric oxides observed in basaltic soils analyzed by the Mars Exploration Rovers (102). Within the TLS dataset, mineralogical sources of water evolved at temperatures above ~200 °C include interlayer water from various phyllosilicates [100 to 300 °C (103)], akaganeite [dehydroxylation, ~200 to 300 °C (104, 105)], dehydration of hydrated salts, perchlorates [100 to 330 °C (106, 107)], and sulfates [100 to 400 °C (108–110)], jarosite [dehydroxylation, the temperature range for which depends on the identity of the primary cation, ~200 to 400 °C (111–113)], and smectite (dehydroxylation, the temperature range for which depends upon the clay mineralogy). Smectite dehydroxylation temperatures range from ~300 to 600 °C (dioctahedral) and ~600 to 900 °C (trioctahedral) (e.g., refs. 114–116). There are exceptions to these temperatures that can be caused by interlayer cations [e.g., Fe-nontronite dehydroxylation begins at temperatures <300 °C (117)]. Depending on the exact temperature cut received by the TLS, mixing of water extracted from some of these minerals also occurs. Complications to isotopic interpretations associated with the receipt of mineralogical water mixtures are presented in *SI Appendix*, *Supplementary Discussion*. Of note, the TLS measures the infrared absorption lines of molecular water and its isotopologues rather than, e.g., HD and H_2_; thus, only those effects that lead to isotopic fractionation manifested in molecular water would be observed.

### SAM-TLS Data Processing and Isotope Ratio Retrieval.

The TLS data processing technique for water isotopes has been described in an earlier publication (21) that is updated here. After gas delivery and cell closure, the near-infrared laser scans over the water lines near 2.78 µm wavelength (3593.6 cm^–1^) every second, and on-board, TLS captures average spectra over sequential 50-s periods that are downloaded with associated housekeeping data. Analysis of each of these ~1-min spectra was done on a spectral line-by-line basis through comparison with HITRAN 2016 calculations [that employ SMOW terrestrial δD and δ^18^O isotopic values (118)], so that volume mixing ratios in ppmv were retrieved for each of the target lines. TLS recorded both direct absorption and second-harmonic spectra simultaneously; however, the water isotope spectra were processed using only the direct absorption spectra, integrating over each identified line to retrieve individual volume mixing ratios for direct comparison with HITRAN. Minor wholesale adjustments (2% for the H_2_^18^O line and 5% for the HD^16^O line) to the HITRAN line strengths were made as a result of prelaunch calibrations using the flight instrument TLS with water isotope standards including SMOW, and as a result of improved He-broadening coefficients from laboratory studies of these same spectral lines (119). Without these minor adjustments (referred to as “multipliers”), direct comparison with HITRAN would produce δD_VSMOW_ and δ^18^O_VSMOW_ values 50‰ higher and 20‰ lower, respectively.

Each TLS EGA run typically produced ~20 sequential spectra whose analysis produced ~20 sets of the retrieved volume mixing ratios for H_2_^16^O, HDO, and H_2_^18^O. Within each set, isotopic delta values were calculated and plotted in time sequence. These ~20 points for each delta value were then statistically analyzed to produce mean δD and δ^18^O values with SE of either 67% CI or 95% CI normalized to the terrestrial VSMOW reference expectations. *SI Appendix*, Fig. S5 shows an example of retrieved isotopic delta values for Glen Etive 3, each point representing analysis of a ~1-min averaged spectrum. For these plotted points, statistical analysis produces results of δD_VSMOW_ = 3,906 ± 20‰ and δ^18^O_VSMOW_ = 49 ± 8‰ (CI 95%) as given in *SI Appendix*, Table S1. See *SI Appendix*, *Supplementary Discussion*, Table S3, and Fig. S8 for additional methodological information and assessment.

## Supplementary Material

Appendix 01 (PDF)

## Data Availability

All SAM-TLS EGA data are available on NASA’s Planetary Data System (PDS) Geoscience node (https://pds-geosciences.wustl.edu/msl/msl-m-sam-2-rdr-l0-v1/mslsam_1xxx/data/) (120) and are identifiable by the test identification (TID) number listed in *SI Appendix*, Table S1. All other data are included in the manuscript and/or *SI Appendix*.

## Footnotes

^*^δXVSMOW = RxsampleRxVSMOW − 1 × 1000 where *X* corresponds to D or ^18^O, *R* refers to the isotope ratio *x*, in this case either D/H or ^18^O/^16^O, and δ values (i.e., δD or δ^18^O) are given in units of per mille (‰) with respect to the Vienna Standard Mean Ocean Water (VSMOW) terrestrial standard.
