# Supplementary material for: Oxygen isotopic evidence that Gale crater, Mars, was home to an Early Hesperian water reservoir that underwent significant evaporation
Source: Proc Natl Acad Sci U S A. 2025 Oct 20;122(43):e2511627122. doi: 10.1073/pnas.2511627122 (PMC12582284; doi:10.1073/pnas.2511627122)
Supplement: Supplementary file 1 — Appendix 01 (PDF) [file pnas.2511627122.sapp.pdf]

PNAS

Supporting Information for
Oxygen isotopic evidence that Gale crater, Mars was home to an Early Hesperian water reservoir that underwent significant evaporation

Amy E. Hofmann[1*], P. Douglas Archer Jr.[2,3], Amy C. McAdam[4], Brad Sutter[2,3], Thomas F. Bristow[5], John M. Eiler[6], Christopher R. Webster[1], Gregory J. Flesch[1], Abigail A. Fraeman[1], Heather B. Franz[4], John P. Grotzinger[6], Christopher H. House[7], Elizabeth B. Rampe[2], Jennifer C. Stern[4], Paul R. Mahaffy[4], Charles A. Malespin[4], and Ashwin R. Vasavada[1]

[1]Jet Propulsion Laboratory, California Institute of Technology, Pasadena, CA 91109, USA
[2]NASA Johnson Space Center, Houston, TX, 77058, USA
[3]Jacobs Technology, Houston, TX, 77258, USA
[4]NASA Goddard Space Flight Center, Greenbelt, MD 20771, USA
[5]NASA Ames Research Center, Mountain View, CA 94043, USA
[6]Division of Geological and Planetary Sciences, California Institute of Technology, Pasadena, CA 91125, USA
[7]Department of Geosciences, Pennsylvania State University, University Park, PA 16802, USA

*Corresponding Author: Amy E. Hofmann
Email: amy.e.hofmann@jpl.nasa.gov

This PDF file includes:

Supporting Information Text:
- Supplementary Discussion
- Supplementary Methods

Figures S1 to S14
Tables S1 to S7
SI References

SI Table of Contents

Click the TOC entry to jump to the corresponding section within the document

SUPPLEMENTARY DISCUSSION 3

SAM-TLS data within the context of other martian water analyses 3

Oxygen isotope analyses of martian H_2O 3

Hydrogen isotope analyses of martian H_2O 5

Geologic context and sample descriptions 6

Further analysis of the TLS evolved water $^{18}O/^{16}O$ and D/H measurements 8

Identification of outliers and data culling 10

Isotopic analyses by primary mineralogical water source 11

Mixtures of structural hydroxyl and molecular water 12

Structural molecular and adsorbed water 12

Dissolved water in poorly crystalline phases 14

Predicting the $^{18}O/^{16}O$ composition of readily-exchangeable water 15

Complications to isotopic interpretations 16

Accuracy of the HITRAN-based approach for calculating the oxygen isotopic compositions of ^{18}O-enriched waters 23

SUPPLEMENTARY METHODS 28

Numerical Modeling I. H_2O–CO_2 oxygen isotope exchange 28

Calculation variables and parameter space 29

Model assumptions, rationale, and implementation 29

Numerical Modeling II. Linear relationships in isotope ratio space 33

Diffusion-driven fractionation within the SAM instrument 36

Mineral-water equilibrium fractionation 37

Rayleigh fractionation via evaporation into an infinite atmosphere under conditions of liquid-vapor thermodynamic equilibrium 40

Craig-Gordon model for evaporative fractionation into a low-humidity atmosphere of constant isotopic composition 43

SI TABLES 48

SI REFERENCES 57

A NOTE TO READERS: All figures and tables called out in the Supplementary text have been hyperlinked such that, by clicking on the bolded figure or table reference (e.g., **Figure S1** or **Table S1**), the reader can jump to the associated figure or table entry within the document. Figures are embedded within the text closest to their first mention; tables appear at the end of the document.

SUPPLEMENTARY DISCUSSION

SAM-TLS data within the context of other martian water analyses

Oxygen isotope analyses of martian H_2O

Studies investigating the oxygen isotopic composition of martian materials have primarily focused on bulk rock and igneous silicate mineral analyses, which together tightly constrain the oxygen isotopic composition of the martian mantle to be of order $\delta^{18}O_{VSMOW} = 4.5 \pm 0.8$‰ (average and standard deviation of analyses presented in (1-9)), which is slightly less than that of Earth's mantle (~5.5‰; (10)). Some authors have investigated the oxygen isotopic compositions of phosphates (11, 12) and carbonates (13-18) in martian meteorites via in situ analyses, while others have performed acid digestion experiments (19-27) or step-wise heating experiments (28-31) on bulk rock powders (all plotted in **Figure S1**). Among these investigations, only the studies reported in (28-31) resemble the SAM-EGA experiments described here.

Although bulk rock analyses presented in (28) yielded $\delta^{18}O_{VSMOW}$ values consistent with values previously reported for shergottite, nakhlite, and chassignite (SNC) meteorites (i.e., 4 to 6‰), nearly all of the evolved water samples (including the terrestrial control) in the corresponding step-wise heating experiments yielded negative $\delta^{18}O_{VSMOW}$ values. Including all temperature cuts for all meteorites, the evolved water $\delta^{18}O_{VSMOW}$ values span nearly a 35‰ range from roughly –23 to +12‰, and—although the $\delta^{18}O_{VSMOW}$ values differed among the different meteorites—the water released in the 600–1000°C step was consistently the most ^{18}O-depleted from each. Concerned that this pattern reflected a kinetic isotope effect associated with the vacuum pyrolysis approach to dehydration, (28) did not attempt to interpret the $\delta^{18}O$ data further. Similar vacuum pyrolysis experiments by (30) on NWA 7034 yielded negative $\delta^{18}O_{VSMOW}$ values of roughly –20 to –1.5‰ between temperatures of 50 and 500°C. In contrast, water released at 1000°C from Lafayette in the (28) study and NWA 7034 in the (30) study yielded positive $\delta^{18}O_{VSMOW}$ values of 5‰ and 11‰, respectively. These values are within error of the lowest Gale crater $\delta^{18}O$ values, nearly all of which correspond to water evolved from mixtures that include hydrated salts and dissolved water from poorly crystalline phases (**Table S1**). Thermodynamically, water bound to these phases should be much more exchangeable than water released during dehydroxylation of phyllosilicates and jarosites. Waters released during clay mineral dehydroxylation in Gale samples cluster around a median $\delta^{18}O_{VSMOW}$ value of ~38‰, which suggests that the local hydrologic reservoir present at Gale must have been more ^{18}O-enriched than either the source(s) of water entrained in martian meteorites or most terrestrial hydrologic reservoirs (**Figure S1**).

The highest $\delta^{18}O$ values in the SAM-TLS evolved water dataset are twice the smectite median value (**Table S1**; **Figure S1**), and it may be tempting to attribute those isotopic signatures to near complete exchange with ^{18}O-enriched atmospheric water vapor. To date, the oxygen isotopic composition of modern martian atmospheric water vapor has been reported from measurements made by three instruments. In chronological order, they are the Fourier Transform Spectrometer onboard the *Kuiper Airborne Observatory* (Earth-based, full disk: $\delta^{18}O_{VSMOW} = -100 \pm 33$‰; (32)); the Atmospheric Chemistry Suite onboard the *ExoMars Trace Gas Orbiter* (remote-sensing, solar occultation, vertical profile

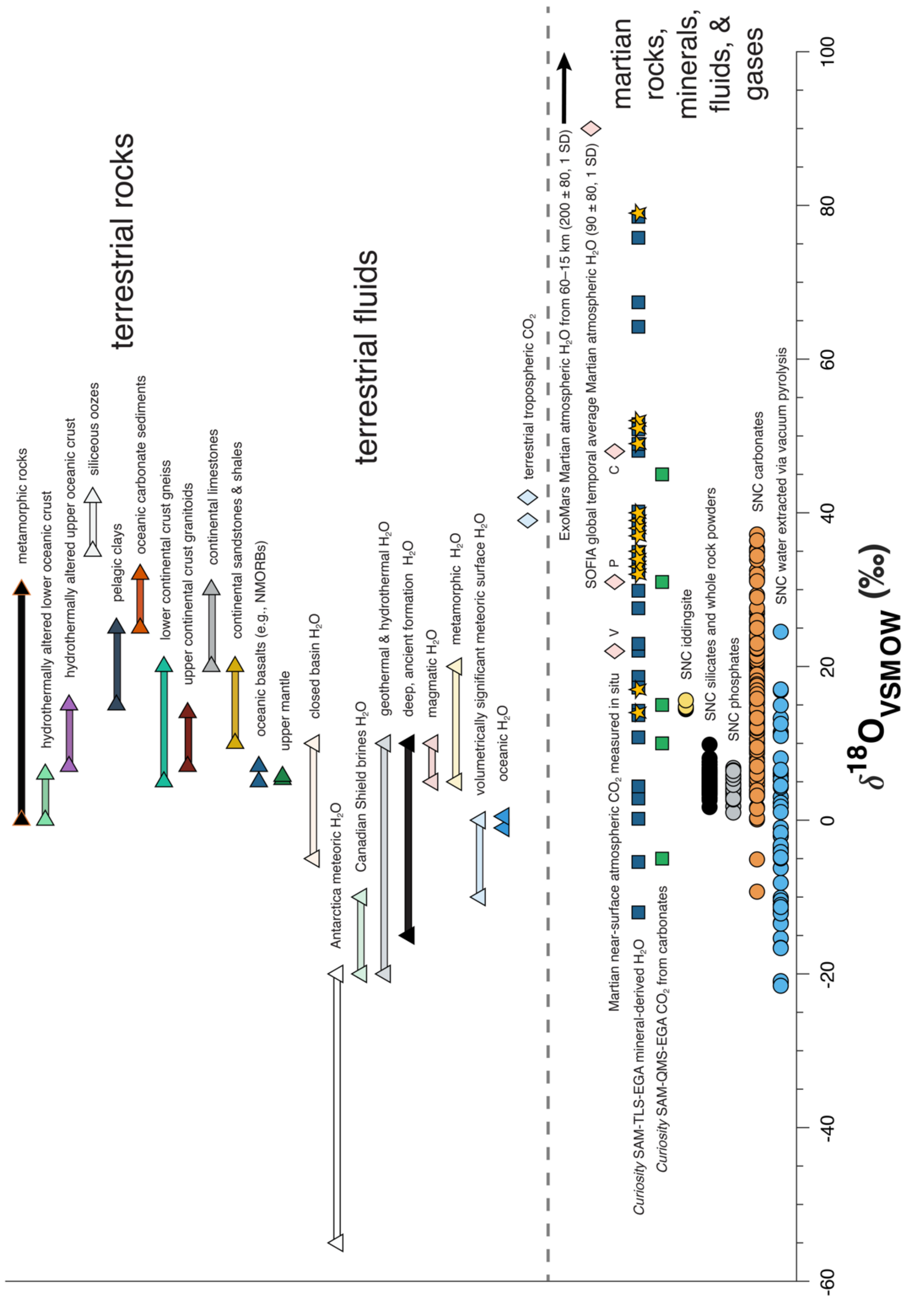

Figure S1. Compilation of oxygen isotopic compositions of various martian and terrestrial materials. Compositional ranges in terrestrial materials are presented above the gray dashed line and are indicated by horizontal bars between two end members. Terrestrial rocks and fluids are represented using right-facing

arrows and triangles, respectively; data can be found in (10, 33, 34). Terrestrial tropospheric CO_2 data are from (35, 36). The mean in situ oxygen isotope measurements of martian atmospheric CO_2 are from *Viking* (V) (37), *Phoenix* (P) (38), and *Curiosity* (C, via TLS) (39). SAM-TLS mineral-derived water isotopic data (blue squares) can be found in **Table S1**. Stars denote water sourced from Gale clay minerals. SAM-QMS mineral-derived isotopic data for carbonate-evolved CO_2 are limited to data collected within the clay-bearing units published in (40). Laboratory oxygen isotope data from martian SNC meteorites were compiled from the following references: iddingsite (41), silicates and whole rock powders (1-9, 30, 41, 42), phosphates (11, 12), carbonates (ion microprobe (13-17, 27); acid liberation of CO_2 (20-23, 25-27, 43); vacuum pyrolysis (29)), and water extracted via vacuum pyrolysis of bulk samples (28, 30, 31).

weighted average of all data from altitudes between 15–60 km: $\delta^{18}O_{VSMOW}$ = 200 ± 80‰, weighted 1 SD (44)); and the EXES instrument on the *Stratospheric Observatory for Infrared Astronomy* (*SOFIA*; Earth-based, globally and temporally averaged: $\delta^{18}O_{VSMOW}$ = 90 ± 80‰, weighted 1 SD (45)). The *ExoMars* and *SOFIA* values are consistent with one another, overlapping at the 1-sigma level and indicative of ^{18}O enrichment in modern upper atmospheric water vapor relative to the terrestrial standard. Although SAM-TLS was able to measure the hydrogen isotopic composition of an ingest of martian near-surface water vapor, no oxygen isotope data were reported due to the extremely low signal-to-noise in the measurement. In lieu of a true atmospheric sample, (39) interpreted water evolved from Rocknest aeolian fines over the temperature range 234–425°C to be adsorbed atmospheric water vapor. However, as discussed in the ***'Predicting the $^{18}O/^{16}O$ composition in readily-exchangeable water'*** section below, the rapid rate of oxygen isotope exchange between gas-phase CO_2 and thin film liquid (or adsorbed) H_2O should drive readily-physically-exchangeable H_2O in contact with martian near-surface atmospheric CO_2 (having a $\delta^{18}O_{VSMOW}$ of approximately 48‰ (39)) towards $\delta^{18}O_{VSMOW}$ values around 0‰ given sufficient time to approach isotopic equilibrium. Near-surface water vapor should be even more ^{18}O-depleted than this H_2O adsorbate (or thin film or frost) due to the fractionation associated with vapor-adsorbate (or vapor-thin film or vapor-frost) isotopic exchange. The large positive $\delta^{18}O_{VSMOW}$ values reported for water in the martian upper atmosphere from remote sensing observations may thus reflect oxygen isotopic disequilibrium driven by isotopically fractionating photochemical reactions and/or interaction with the solar wind and subsequent loss of ^{16}O to space (e.g., (46)) and not be representative of water vapor and frost in the modern martian near-surface environment.

Hydrogen isotope analyses of martian H_2O

Numerous studies have documented the hydrogen isotopic compositions of various mineral phases and glasses from martian meteorites as well as in bulk samples. In studies focused on silicate minerals and glasses, high δD measurements (i.e., 1000s of per mille enriched with respect to VSMOW) have largely been interpreted to reflect the composition of ancient martian crustal and/or atmospheric water reservoirs while low δD values (i.e., depleted or enriched with respect to VSMOW by only ~10s to 100s of per mille) are typically interpreted as evidence of either terrestrial contamination or a primitive martian magmatic component (e.g., (47-53)).

The only martian meteorite hydrogen isotope data directly relevant to the TLS EGA dataset come from (29) and (30), who used stepwise heating experiments to measure the hydrogen isotopic composition of water thermally evolved from SNC rock powders. The lowest temperature cuts (~50 to ~325°C) in the study of martian meteorite NWA 7034

evolved water with negative δD_{VSMOW} values of approximately –140 to –90‰ (30). Although the negative values could be evidence of terrestrial alteration, all evolved water had $\Delta^{17}O$ anomalies, which the authors interpreted as evidence for the waters' martian origin. Stepwise heating of various SNC whole rock powders by (29) produced water with δD_{VSMOW} values as low as –75‰ at 120°C—i.e., comparable to the –90‰ measured by (30) for water released from NWA 7034 over a ~150–325°C temperature ramp. In contrast to Agee et al. (30), Leshin et al. (29) interpreted their negative δD_{VSMOW} values as reflecting terrestrial contamination. Both studies report positive δD_{VSMOW} values for water evolved at temperatures >325°C, with the greatest measure of D enrichment in the highest temperature water release: δD_{VSMOW} of ~330‰ for NWA 7034 at 1000°C (30) and δD_{VSMOW} values from ~265 to 2140‰ for extractions ≥ 600°C from different SNC meteorites (29).

Leshin et al. (29) interpreted their high positive values as consistent with a martian origin based on 1) a current martian atmospheric water vapor δD_{VSMOW} value of ~4000‰, 2) the assumption that H has been preferentially lost (relative to D) from the martian atmosphere since the time when those meteoritic minerals crystallized, and 3) the fact that many SNCs carry secondary phases indicative of low-temperature aqueous alteration by crustal fluids that likely became D-enriched via isotopic exchange with the martian atmosphere. As shown in **Figure 1** of the main text, the Gale samples' hydrogen isotopic compositions span a range of over 1500‰, and nearly all Gale samples have δD_{VSMOW} ≥3000‰, i.e., approaching modern martian atmospheric water vapor values based on numerous Earth-based observations and various spacecraft at Mars (e.g., (32, 45, 54-63)). As discussed in the main text in conjunction with **Figure 2**, it is plausible that the same explanation applies to the Gale crater samples' hydrogen isotopic compositions: they reflect incorporation of variably, but highly, D-enriched fluids during mineral formation.

Geologic context and sample descriptions

Based on crater-counting estimates, Gale crater formed between ~3.8 to 3.6 billion years ago (Ga) (64, 65). Potassium-argon dating of an aliquot of Sheepbed mudstone sampled at the Cumberland site indicates that the mean age of materials in Gale crater exceeds 3.6 Ga, making it Hesperian in age and consistent with the sediments' deposition during early infilling of the crater (66). Remote sensing data—including infrared, visible, imaging, and topographic information—show that the crater rim is dissected with fluvial channels, all flowing into the crater (67, 68). The valley networks and associated catchment basin(s) drain upland plains, which, along with the crater rim and walls, likely source the detrital material observed within Gale itself (68-72). Among multiple fans, deltas, and channels identified in and around Gale crater, the Peace Vallis alluvial fan extends from the crater's northwest rim, terminating in an enclosed topographic basin in the vicinity of Yellowknife Bay formation, the basal member of the Bradbury group; however, the relationship between the alluvial fan and Yellowknife Bay sedimentary rocks has not been definitively determined (68, 73). Geomorphological evidence supports at least three major lake stands in the crater following the formation of Mount Sharp, suggesting that the sedimentary rocks *Curiosity* has encountered have experienced several wet-dry cycles with the lowest sedimentary rocks (i.e., those of the Yellowknife Bay formation) among the last to dry out, possibly as part of a period of small, shallow, localized lake formation and evaporation (70).

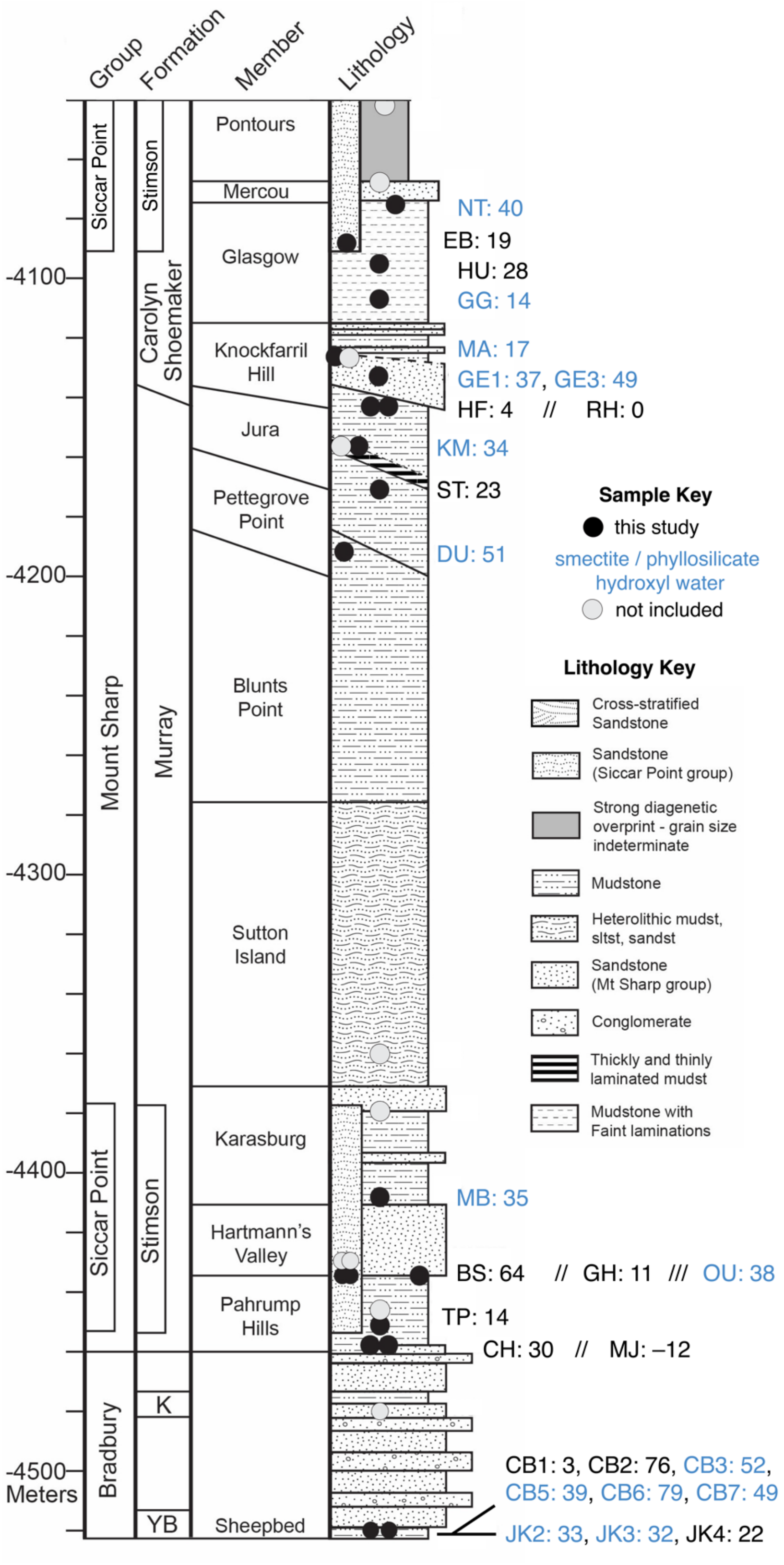

Figure S2. Oxygen isotopic compositions of water evolved from a subset of samples (black circles) identified in **Table S1**. Numbers next to each sample abbreviation are given in units of $\delta^{18}O_{VSMOW}$ per mille (‰). Samples are identified within their sedimentary and stratigraphic context. Data for samples from the same stratigraphic horizon (horizontally adjacent black circles) are separated by '//', except for John Klein (JK) and Cumberland (CB), which are separated due to space constraints. Lithologies as noted in the legend. Additional details are provided in **Table S2**. Aeolian samples from Rocknest (RN) and Gobabeb (GB) are not plotted.

"Not included" (gray circles) denotes samples in the stratigraphic sequence that either were not sent to SAM for evolved gas analyses or were identified in this SI as outliers. YB = Yellowknife Bay; KB = Kimberley.

Yellowknife Bay's thinly bedded mudstones (Sheepbed) underlie sheetlike sandstones with poorly defined crossbedding (Gillespie Lake), which are themselves overlain by the cross-stratified sandstones and interleaved siltstones of the Glenelg member; this coarsening-upward sequence is consistent with deposition in a fluvio-lacustrine setting (73). Remote-sensing data show that the central mound consists of a lower formation of gently dipping, parallel beds dominated by clay mineralogy (i.e., the Bradbury group), the uppermost facies of which interleave with the lowermost facies of the Mount Sharp group, which in turn transition from hematite- and clay-bearing lithologies into rocks dominated by sulfates (74, 75). This lithologic transition has been interpreted as indicative of a shift from a warmer, wetter climate and low-salinity, circumneutral-to-alkaline aqueous conditions in Gale to a colder, drier climate favoring the precipitation of salts under more acidic and oxidative aqueous conditions (e.g., (76) and references therein). Data from *Curiosity* have enabled mapping of over 620 meters of elevation gain of outcropping sedimentary strata, the lithologies of which are consistent with associated fluvial, deltaic, and lacustrine depositional environments (some of which were sculpted later by aeolian processes)—as well as active and ancient dry aeolian environments (77-80)—and continued diagenetic alteration extending long past the depositional stages (72, 81-102). Additionally, certain regions contain both physical and geochemical evidence for episodic drying and refilling of the lake (103-109). The lithologies associated with the SAM-TLS data presented herein are limited to those sampled prior to entering the clay-sulfate transition region and are identified in **Table S2**. The corresponding stratigraphic column—annotated with the oxygen isotopic compositions of evolved water from each sample—is given in **Figure S2**. The overall mineralogy—particularly the lack of soluble salts—and geochemistry of the clay-rich sediments broadly support a low-salinity, circumneutral aqueous environment during sediment deposition (e.g., (76) and references therein). In contrast, the chemistry and mineralogy of diagenetic features within certain strata indicate variable groundwater compositions of different pH, alkalinity, salinity, and solute compositions; in some sections, the variety of late diagenetic features likely reflect multiple generations of fluid infiltration (e.g., (110-112)).

Further analysis of the TLS evolved water $^{18}O/^{16}O$ and D/H measurements

As described in the **Methods** section of the main text, water thermally evolved during SAM-EGA experiments is primarily derived from mineralogical sources. **Figure S3** differs from **Figure 1** in the main text in that here we include outliers and other analyses that were omitted from **Figure 1**. The data analysis and interpretation subsections that follow include: 1) the rationale for data culling as reflected in all figures save **Figure S3**, 2) a brief discussion of the isotopic data for mineralogical sources more susceptible to isotopic exchange followed by 3) an interpretation of the oxygen isotopic compositions of readily exchangeable waters within the dataset, and 4) a presentation of several possible means by which the isotopic composition of martian water measured by the TLS could have been altered prior to analysis.

A final note: In laboratories on Earth, we have the luxury of running various 'blank' analyses to ascertain the contributions of, e.g., a wet-chemistry method to the signal observed during the analysis of an actual sample or standard. Such 'blanks' are essential for making background corrections and, thus, robust interpretations of the collected data.

Given planetary mission resource limitations, however, running a ‘blank cup’ experiment before each SAM-EGA experiment is not practical or, in some cases, feasible. Furthermore, applying the compositional background from a single blank cup run as a general correction to later EGA experiments is not appropriate due to differences in experimental parameters among different runs and the fact that volatiles observed in a preceding blank may be driven off during the temperature ramp for the next sample analysis. Finally, the extremely low water concentrations observed in SAM-EGA blank cup experiments leads to extraordinarily low signal-to-noise ratios (with concomitantly large errors) in the TLS water spectra, particularly in the oxygen isotope data. For these reasons, we have not applied any ‘blank cup’ corrections to data presented here.

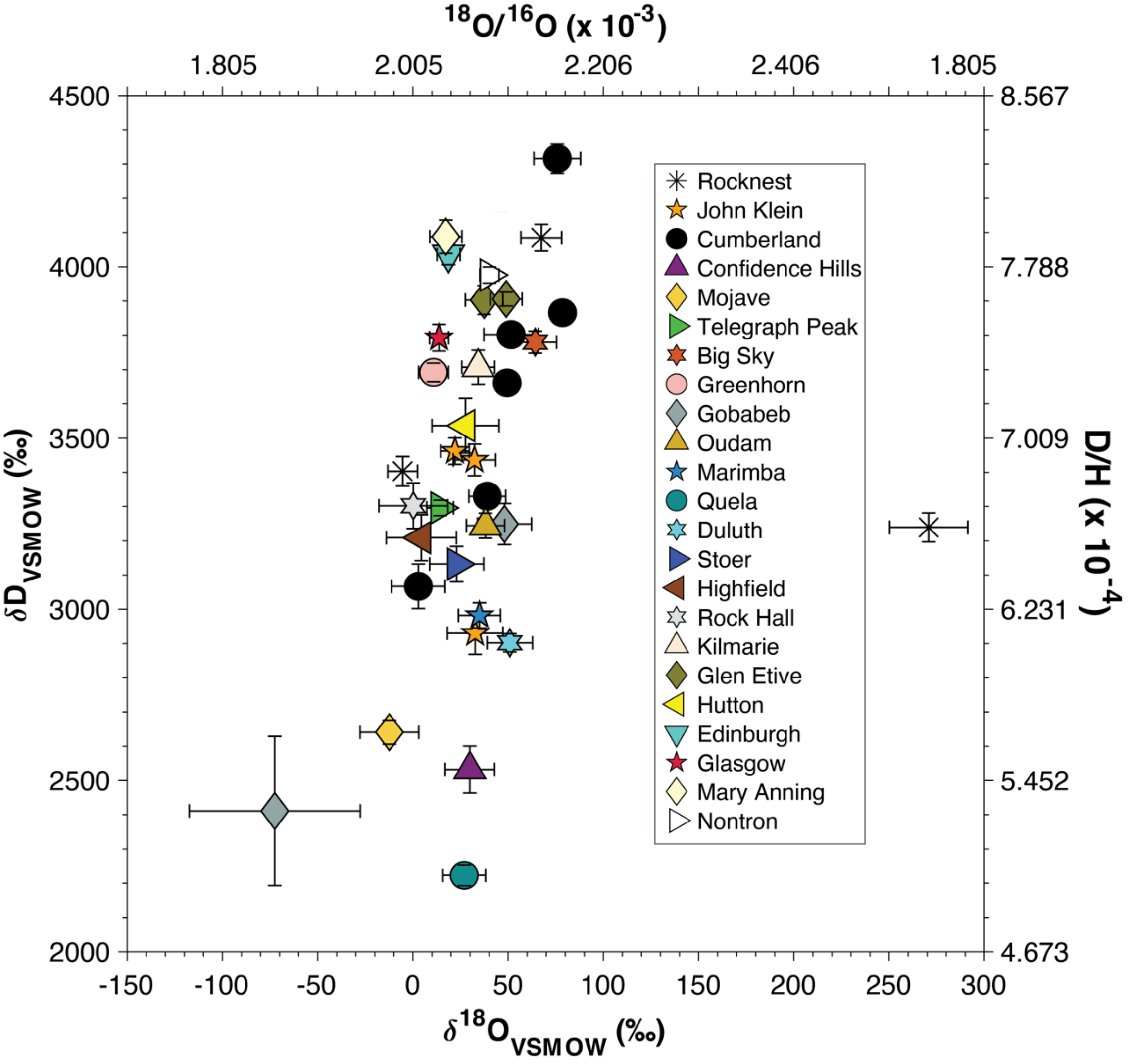

Figure S3. Complete SAM-TLS evolved water isotope dataset as presented in **Table S1**. Data points included here but excluded from Figure 1 in the main text include scooped (aeolian) samples as well as the isotopic outliers identified in the Supplementary Text. Delta notation is given with respect to the VSMOW terrestrial standard, i.e., $^{18}O/^{16}O = 2.005171 \times 10^{-3}$ and $D/H = 1.5576426 \times 10^{-4}$ based on relative abundances of the isotopes given in (113). Error bars represent two standard error of the mean.

Identification of outliers and data culling

Brief descriptions of the Rocknest 1 and 2 (RN1, RN2), Gobabeb 1 (GB1), and Quela (QL) samples are presented here. The oxygen isotopic composition of RN1 was not measured; because this manuscript focuses on the paired D/H and $^{18}O/^{16}O$ in situ measurements of rock powders from Mars, there is no place for RN1 in our analysis space. The δD_{VSMOW} of RN1 was previously discussed in (114) and is not reproduced here. The hydrogen isotopic composition of RN2 (δD_{VSMOW} = 3239 ± 42‰, 2 SE) is within error of almost all of the other hydrogen isotope values presented in **Table S1**; however, its oxygen isotopic composition ($\delta^{18}O_{VSMOW}$ = 271 ± 21‰, 2 SE) is nearly a factor of 3 greater than the next-highest oxygen isotope measurement, that of Cumberland 2 (CB2), which has a $\delta^{18}O_{VSMOW}$ = 79 ± 4‰ (2 SE). Water from the RN2 TLS cut is attributed to residual adsorbed water and that from hydrated salts (**Table S1**). Such a high, positive $\delta^{18}O_{VSMOW}$ value could represent the end-product of nearly complete evaporation of ^{18}O-enriched water following a Rayleigh distillative process under relative humidity conditions (P/P_0) of nearly 0; however, it is not obvious why the corresponding D/H of the evolved water would appear unaffected (i.e., be within the error bounds of nearly all other measurements).

Subsample GB1 produced the second-lowest hydrogen isotopic composition (δD_{VSMOW} = 2411 ± 218‰, 2 SE) and the greatest uncertainty among all hydrogen isotope analyses. Water received by the TLS from GB1 evolved over a temperature range of 34–147°C and is attributed to a mixture of residual adsorbed water and water released from hydrated salts in this aeolian dune sample (**Table S1**). A second subsample of Gobabeb (GB2) was also analyzed, for which the TLS received water evolved over a much higher temperature range (i.e., 534–798°C). Water evolved from GB2 was attributed to the same components as GB1 plus additional contributions of dissolved water released from poorly crystalline phases. The hydrogen isotopic compositions of water from GB1 and GB2 differ by ~800‰, likely due to the evolved water coming from very different minerals that decompose over the extremely different temperature cuts sampled by TLS. The two analyses were performed on separately collected subsamples: GB1 consisted of finer-grained material (sieved powder <150 μm diameter) from Scoop #1 and GB2 (material 150 μm to 1 mm diameter) came from Scoop #2 (115, 116). Evidence for high rates of sediment transport at Gobabeb (116) makes it even more likely that water evolved from GB1 and GB2 came from different minerals and/or were sourced from different sediments, transported to, and intermixed at the Gobabeb site. More significant than the low D/H ratio in GB1 is its anomalous oxygen isotopic composition: $\delta^{18}O_{VSMOW}$ = –73 ± 45‰ (2 SE). In contrast, GB2 has an oxygen isotopic composition of $\delta^{18}O_{VSMOW}$ = 48 ± 14‰ (2 SE), which is within uncertainty of the median of the culled population. Only Rocknest 4 (RN4) and Mojave (MJ) also have negative $\delta^{18}O$ values, but these are ~15 and ~6 times less negative than GB1, respectively: $\delta^{18}O_{VSMOW}$ = –5 ± 8‰ (RN4, 2 SE) and –12 ± 15‰ (MJ, 2SE) (**Table S1**). The evolved water from these latter two samples comes from a mixture of adsorbed water and various hydrated salts (including perchlorates), but neither of these two measurements is resolved at two standard error from a $\delta^{18}O_{VSMOW}$ of 0‰. There are certain physical conditions under which a negative $\delta^{18}O_{VSMOW}$ value in martian water could arise. For example, near-surface atmospheric water vapor in oxygen isotopic equilibrium with near-surface atmospheric CO_2 could have a $\delta^{18}O_{VSMOW}$ as depleted as –10‰. Water

residual to freezing would also be depleted in ^{18}O relative to the $^{18}O/^{16}O$ composition of the water ice.

As noted in '***SAM-TLS data within the context of other martian water analyses***', negative $\delta^{18}O_{VSMOW}$ values have been reported in oxygen isotope datasets from meteorite stepwise heating experiments (28, 30, 31). In the vacuum pyrolysis experiments reported in (28), ground powders of multiple SNC meteorites were heated to a set temperature, held at that temperature for one hour, and then ramped to the subsequent temperature of interest. With the exception of the Lafayette meteorite, Karlsson et al. (28) measured negative $\delta^{18}O_{VSMOW}$ values for water evolved from all SNC meteorites at nearly all temperature plateaus (i.e., 150°C, 350°C, 650°C, and 1000°C) down to values of $\delta^{18}O_{VSMOW} \sim -20‰$ at the highest temperatures (numerical data provided in (31)). Based on the martian host rock bulk isotopic compositions ($\delta^{18}O_{VSMOW} \sim +4.5‰$) and the fact that the terrestrial control also exhibited unexpected negative $\delta^{18}O_{VSMOW}$ values, (28) interpreted these data as indicative of a strong kinetic isotope effect associated with dehydration during vacuum pyrolysis. In an attempt to minimize contamination, reactions between the evolved water and remaining meteorite powder, and kinetic isotope effects, Agee et al. (30) and Maltsev (31) performed their step-heating experiments under flowing He gas rather than under vacuum; their experiments are thus more closely aligned to the SAM-EGA experiments than those by (28). Despite these procedural modifications, both (30) and (31) measured negative $\delta^{18}O_{VSMOW}$ values in meteoritic evolved water. The ^{18}O data were reported by (30) with respect to the associated $\Delta^{17}O$ values but not discussed further. Maltsev (31) attributed the negative $\delta^{18}O_{VSMOW}$ values to some combination of mixing (possibly terrestrial contamination and/or oxygen exchange between different sites within hydroxylated minerals) and kinetic effects. Given the extreme magnitude of ^{18}O depletion in GB1 evolved water (unlike all other analyses in the TLS EGA dataset) and our inability to rule out experimental artifacts and/or associated kinetic isotope effects, we have chosen to exclude GB1 from further analysis.

The lowest hydrogen isotope ratio ($\delta D_{VSMOW} = 2223 \pm 31‰$, 2 SE) measured by TLS was of water from Quela (QL) that evolved over a temperature window of 432–895°C and was attributed to smectite dehydroxylation. The TLS-measured oxygen isotopic composition ($\delta^{18}O_{VSMOW} = 27 \pm 11‰$, 2SE) is within one standard error of the mean over all TLS water oxygen isotope measurements except for the outliers identified above. However, as indicated in **Table S1** the Quela sample sat in Curiosity's Sample Handling System (SHS) for almost 300 sols prior to SAM analysis. Although hydroxyl waters are expected to be largely robust against hydrogen (and oxygen) isotope exchange under ambient pressures and temperatures, the effects of prolonged storage within the SHS on the hydrogen isotopic composition of mineralogical water(s) are unknown. Due to Quela's anomalous handling and the fact that this manuscript focuses on paired hydrogen and oxygen isotopic data from evolved waters, we have chosen to treat Quela as an outlier.

Isotopic analyses by primary mineralogical water source

For the purposes of this manuscript, we define three operational categories of water: that which evolves over low, medium, and high temperatures ranges—generally corresponding to adsorbed, structurally bound molecular water, and structurally bound hydroxyl, respectively. These three 'types' of water can be placed on a continuum according to the amount of energy required to break mineral-water bonds, which we take

as a proxy for the exchangeability of mineralogically bound and 'free' water (e.g., atmospheric water vapor). For a given set of intensive thermodynamic properties and time, adsorbed water is most easily exchanged, while structural molecular water (e.g., waters of hydration in gypsum) is less readily exchanged, and structural hydroxyl groups (e.g., in clays or jarosite) are least easily exchanged over geologic timescales. Structural hydroxyl waters are the focus of the main text; their isotopic compositions are reproduced here in the upper left panel of **Figure S4**. In the text that follows, we present the remainder of the dataset, classified by primary mineralogical water source. The isotopic compositions support our interpretation based on the smectite data, but these other data are relegated to the Supplement due to the increased susceptibility of non-hydroxyl waters to isotopic exchange.

Mixtures of structural hydroxyl and molecular water

The upper right panel in **Figure S4** presents samples for which the isotopic data correspond to mixtures of water evolved from phyllosilicates (mostly smectite) and from one or more sources of molecular water (e.g., structural water from hydrated salts). The water 'type' (e.g., structural molecular versus structural hydroxyl) and thus relative ease of exchange differs, thus the fractionation factors differ, and the relative abundances of each's contribution to the total water abundance 'seen' by TLS is unknown. The data generally cluster near the median $\delta^{18}O_{VSMOW}$ value for all samples and have hydrogen isotopic compositions more enriched in deuterium than the median. The relative contributions of, e.g., smectite hydroxyl to molecular water from hydrated salts have not been quantified. Although it is plausible that the higher δD_{VSMOW} values for these samples reflect disproportionately large contributions of hydrated-salt-derived molecular water that has undergone some extent of hydrogen isotope exchange with the deuterium-enriched modern martian atmosphere, that hypothesis can be neither confirmed nor refuted at present.

Structural molecular and adsorbed water

Data presented in the lower right panel of **Figure S4** largely reflect structural molecular water evolved from a diverse array of mineralogical sources, including Ca,Mg,Fe-sulfates and Fe-oxyhydroxides, as well as other hydrated salts like perchlorates, chlorates, and Mg,Ca-chlorides. The isotopic compositions measured by the TLS are most likely weighted averages of water (with different isotopic compositions) evolved from different minerals. In some cases, these mixtures may include residual adsorbed water as well. Perhaps unsurprisingly, these data encompass the largest range in isotopic composition space of the mineralogical classifications presented in **Figure S4** and include both the highest and lowest δD and $\delta^{18}O$ values of the culled dataset.

The Confidence Hills (CH) and Mojave (MJ) TLS cuts both consist of water evolved over the second-lowest temperature range of the accepted dataset, 39–156°C. Water from these samples consists of a mixture of adsorbed water (evolved at temperatures below 200°C) and some contribution from hydrated salt decomposition. Within the accepted dataset, CH and MJ also have the two lowest hydrogen isotopic compositions of all samples measured by the TLS: $\delta D_{VSMOW} = 2532 \pm 69‰$ (2 SE) and $\delta D_{VSMOW} = 2641 \pm 35‰$ (2 SE), respectively. Although it is plausible—based on SNC laboratory data—that these values reflect the true isotopic compositions of the hydrated salts, they are not consistent

with expectations for adsorbed water, which should dominate at temperatures <200°C, unless the water were mixed (or isotopically exchanged) with terrestrial water inside SAM.

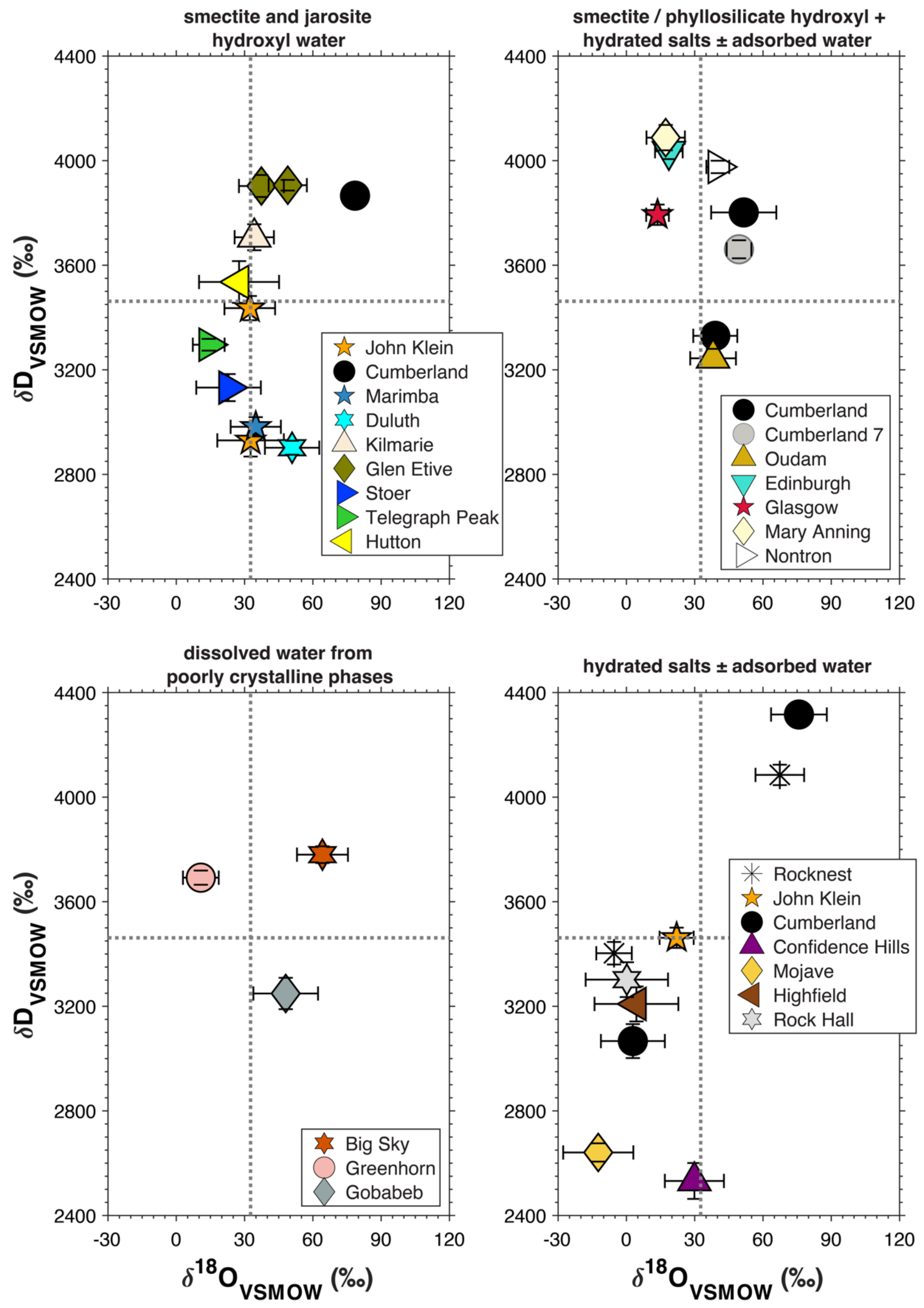

Figure S4. Isotopic compositions of evolved water classified by dominant mineralogical sources as identified in **Table S1**. Error bars represent two standard error of the mean. Dashed gray lines correspond to the median isotopic compositions of the culled dataset. Outliers not included.

If the water were adsorbed martian atmospheric water vapor, one would expect to measure a high D/H ratio consistent with either direct adsorption of water vapor from the D-enriched martian atmosphere or with a distillative residue of adsorbed water slowly desorbing (or evaporating / subliming) from the mineral surface. While MJ's $\delta^{18}O_{VSMOW}$ value (–12 ± 15‰, 2 SE) is the most depleted in ^{18}O within the accepted dataset, it is not abnormally so, and CH's $\delta^{18}O_{VSMOW}$ value (30 ± 13‰, 2 SE) is consistent with the median of the culled martian values. The TLS measures absorption lines from molecular water. Thus, explaining a 'normal' $^{18}O/^{16}O$ ratio and an 'abnormal' low D/H ratio in water evolved from the same sample could reflect hydrogen isotope exchange decoupled from that of oxygen or hydrogen and oxygen from two different sources recombining as H_2O.

In contrast, water evolved from CB2 is the most D- and ^{18}O-enriched sample within the TLS dataset (**Table S1**). Its high δD value (δD_{VSMOW} = 4316 ± 43‰, 2 SE) could reflect exchange between D-enriched water vapor in the modern martian atmosphere and the surface-adsorbed and/or molecular water characteristic of many of the hydrated salt candidates for CB2, like sulfates and oxyhydroxides. As noted in **Table S1**, CB2's water release (99–349°C) includes contributions from both adsorbed water and hydrated salt decomposition. Thus, it is plausible that CB2's oxygen isotopic composition reflects significant contributions of isotopically enriched residuum, such as that left behind after the progressive loss of readily exchangeable adsorbed and mineral-bound water molecules into a nearly dry martian atmosphere. This hypothesis could also explain the high δD and $\delta^{18}O$ values of RN3 (**Table S1**).

Dissolved water in poorly crystalline phases

The TLS received water released from the ancient aeolian sediments at Big Sky (BS) and Greenhorn (GH1) and the modern aeolian dunes at Gobabeb (GB). The detrital nature of such material makes provenance determination difficult. All the rocks analyzed by CheMin contain an X-ray amorphous material, the bulk elemental composition of which has been determined by *Curiosity*'s Alpha Particle X-ray Spectrometer (APXS). The identity of the amorphous component (likely comprising more than one material) can be constrained by these data along with full diffraction-pattern modeling (e.g., (117)) and the results of SAM evolved gas analyses (e.g., (115)); however, prescriptive identification of individual components is not possible. Suggestions include various crystalline phases below CheMin detection limits (i.e., <1 wt.%), volcanic or impact glass, hisingerite (or silica + ferrihydrite), amorphous sulfates, and nanophase ferric oxides (118). Given its high temperature of release (~600–800°C), water from BS, GH1, and GB2 may be at least in part released via decrepitation from volcanic or impact glasses. The hydrogen isotopic compositions of water released from these samples do fall within the middle of the range in δD_{VSMOW} values reported for measurements of mafic and feldspathic glasses in martian meteorites (e.g., (47, 48, 51, 119)); however, the meteorite δD_{VSMOW} range spans 1000s of per mille and thus is not particularly diagnostic. To the best of our knowledge, no oxygen isotope data exist for those same meteoritic glasses or for melt or fluid inclusions within silicate minerals. As shown in the lower left panel of **Figure S4**, the oxygen isotopic compositions of evolved water from all three samples are within error of the majority of the smectite and hydrated salt measurements, reflecting the limited overall range in the oxygen isotope dataset.

Predicting the $^{18}O/^{16}O$ composition of readily-exchangeable water

Although adsorbed water released from powdered rock samples at low temperatures is surely in contact with the current martian atmosphere over some time interval, the isotopic composition of that adsorbed water need not be in equilibrium with that of modern, near-surface atmospheric water vapor. This is particularly true for the majority of rock powders analyzed by SAM, which were collected via drilling and thus represent material from >1.5 cm below the rock surface (120). These same statements apply to structural molecular water, such as interlayer water in phyllosilicates or waters of hydration in hydrated sulfate salts. However, without a near-surface atmospheric measurement taken at Gale for comparison (and from which to model the oxygen isotope evolution of martian atmospheric water vapor over time), oxygen isotopic equilibrium between adsorbed or rapidly exchangeable water and water vapor cannot be directly assessed. We can instead predict what the oxygen isotopic composition of the adsorbed/exchangeable water should be based on two plausible physical scenarios. In **Case 1**, we assume that exchangeable H_2O behaves as a liquid thin film; in **Case 2**, we assume that exchangeable H_2O behaves like an adsorbate on a mineral surface. We assume a freshwater composition for the exchangeable H_2O for model simplicity: in **Case 1**, we do not consider any cation or anion contributions to the thin film that might result from mineral surface dissolution; in **Case 2**, we treat the adsorbate as analogous to terrestrial atmospheric water vapor, which has no salinity.

Case (1): A liquid-like thin film of water adsorbed onto mineral surface should isotopically equilibrate with atmospheric CO_2 within tens of hours based on experimentally determined exchange rates at 5°C (121). Using the isotopic fractionation factor $\alpha^{18}_{(\text{CO2,v-H2O,l})}$ for gas-phase CO_2 and liquid H_2O at 10°C (the lowest temperature evaluated experimentally in (122)) of 1.0442, we predict that the $\delta^{18}O_{VSMOW}$ of liquid-phase water in isotopic equilibrium with martian CO_2 having a $\delta^{18}O_{VSMOW}$ of 48‰ (39) should be of order +3.6‰. Extrapolating the α-temperature relationship from (122) to 0°C ($^{18}\alpha$ = 1.0465) leads to a slight decrease in the predicted $\delta^{18}O_{VSMOW}$ of water to +1.4‰. These isotopic compositions are consistent with the five lowest $\delta^{18}O_{VSMOW}$ values in the TLS-EGA dataset: –12, –5, 0, 3, and 4‰ (**Table S1**), all of which are attributed to mixtures of adsorbed water and molecular water lost from hydrated salts. Although not all evolved water attributed to these same components has comparably low $\delta^{18}O_{VSMOW}$ values, many do (**Table S1**, **Figure S4**), suggesting that at least some of the more exchangeable water evolved from Gale crater samples may have isotopically equilibrated with CO_2 in the martian atmosphere.

Case (2): There is precedent for expecting a non-negligible isotopic fractionation between an adsorbate and co-existing vapor: restricted translation, hindered rotation, and coupling between the two among molecules in condensed phases leads to a 'structural effect' that causes fractionation between condensed and vapor phases upon isotopic substitution (123); this effect was shown to apply to adsorbate-vapor systems, albeit with modifications (124). In the case of surface-adsorbed water, the 'structural effect' also manifests in the disruption of the hydrogen-bonding network along the surface and thus the magnitude of fractionation for a given isotopic system (e.g., D/H versus $^{18}O/^{16}O$) can differ depending on surface properties such as the availability of surface 'sites', the orientation of terminating hydroxyls along the surface, and the cation to which either water molecules or hydroxyl groups are bound (125-128). To simplify our calculations, we use the experimentally determined equilibrium fractionation factor (i.e., alpha value) for

oxygen isotope exchange between water vapor and water adsorbed on mesoporous silica (15 nm and 6 nm pore diameters) at 30°C and extrapolated to dry air (i.e., a monolayer of adsorbed water) as given in (128). The smaller of the two pore diameters is comparable to that in nontronite, montmorillonite, and palagonitic dust (~4 nm) evaluated for their water-adsorption capacity under martian conditions (129).

In the water-as-adsorbate scenario, we ask: What would the oxygen isotopic composition of adsorbed / readily-exchangeable water be if the adsorbed / readily-exchangeable water itself were in isotopic equilibrium with atmospheric water vapor, which in turn was in isotopic equilibrium with atmospheric CO_2? **Case (2)** is structured in this way (rather than simply a repeat of **Case (1)** albeit with a different alpha value) because no fractionation factor currently exists for oxygen isotope exchange between adsorbed H_2O and vapor-phase CO_2 or between co-adsorbed species. Based on the relationship among known alpha values for three phases, A, B, and C ($\alpha_{A\text{-}C} = \alpha_{A\text{-}B} / \alpha_{C\text{-}B}$) and the alpha values for oxygen isotope exchange between adsorbed water (A)–water vapor (B) (~1.004; (128)) and between CO_2 vapor (C)–water vapor (B) (1.0587 calculated from beta factors for oxygen isotope exchange at 0°C (130)), we estimate the oxygen isotope fractionation factor between adsorbed H_2O and CO_2 vapor ($\alpha_{A\text{-}C}$) to be of order 0.9484 at ~0°C. Thus, adsorbed water in oxygen isotopic equilibrium with CO_2 having a $\delta^{18}O_{VSMOW}$ value of 48‰ should have a $\delta^{18}O_{VSMOW}$ value of order –6‰, which is within the TLS measurement uncertainties of the $\delta^{18}O_{VSMOW}$ value predicted for a liquid water thin film in oxygen isotopic equilibrium with atmospheric CO_2 in **Case (1)**.

Complications to isotopic interpretations

Although most of the water received by the TLS is attributed to thermal release from minerals, some minor fraction may be derived from other sources. The most common complicating factor is inherent to EGA experimental methodology: namely, that the TLS receives water evolved over a pre-determined temperature window. In most cases, the TLS receives some fraction—rather than the entirety—of the evolved gas peak (e.g., **Figure S5**). Thus, in many cases the analyzed water is a mixture of water from different mineral sources that may differ greatly in their isotopic compositions. Multiple factors can contribute to the isotopic composition of water in or associated with a given mineral. These include—but are not limited to—temperature, salinity, internal energies, extents of reaction, differences in the magnitudes of equilibrium fractionation between liquid water and specific minerals, isotopic exchange between waters from different mineralogical sources or between different types of water in a particular mineral (e.g., interlayer water and hydroxyl groups in clays), kinetic isotope effects associated with diffusion and other processes, and the cumulative effects of the overall chemical and isotopic evolution of the system over time. In the laboratory, such variables can be isolated and their effects on fractionation well-defined. In an in situ planetary context, the integrative effects of such processes are nearly impossible to map onto the isotopic compositions presented in **Table S1**, which are snapshots in time and place from an alien environment less-well-characterized than even ancient terrains on Earth.

Complications can also arise depending on whether the TLS receives water primarily from the beginning or the end of the water evolution curve, both of which are subject to the effects of Rayleigh distillation. The progressive thermal release or vacuum extraction of water—regardless of form (i.e., adsorbed, bound molecular, bound hydroxyl)—from a

geologic material for stable isotope analysis can be modeled as a Rayleigh-type distillation process. 'True' Rayleigh distillation assumes loss from a well-mixed reservoir, which is an oversimplification when applied to volatile loss from minerals due to the compounding effects of diffusion through the crystal lattice. In general, however, the overall effect remains the same: the lightest isotopologue (i.e., $H_2{}^{16}O$) tends to be preferentially released first, such that the remaining water in or adsorbed onto the mineral is enriched in the heavy isotopologues (e.g., $HD^{16}O$, $H_2{}^{18}O$). In the case of incomplete extraction, the isotopic compositions of the two 'pools' of water (i.e., extracted and retained) will reflect this fractionation. The magnitude of heavy-isotope depletion in the extracted water depends upon the fraction of total water recovered, the form of the water, and its mineralogical source. Although diffusive effects can lead to a flattened release profile (and hence a pseudo-steady-state isotope ratio), in general the greater the fraction of water recovered, the closer the isotopic composition of the extracted water will be to that of the actual mineralogical water.

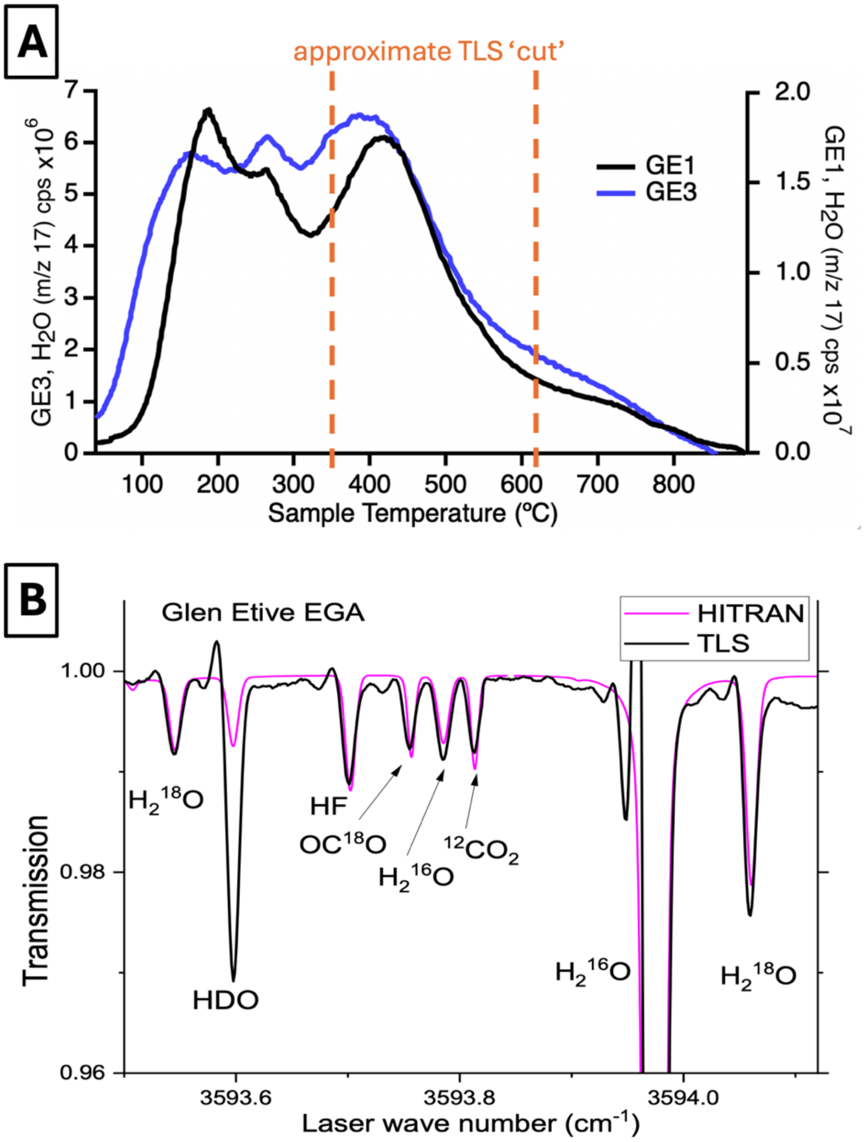

Figure S5 (previous page). (A) EGA trace showing water abundance as a function of temperature for the two Glen Etive TLS measurements. The TLS received water evolved over the temperature range ~350–615°C for both GE1 (black solid line) and GE3 (blue solid line), as identified by the dashed orange lines. The 'humps' in both traces between approximately 320 and 600°C correspond to the temperature range over which water from the dehydroxylation of Fe^{2+} rich smectite is expected to evolve. **(B)** A single spectrum (~1-minute average) from TLS for the Glen Etive sample compared to HITRAN-generated spectrum that uses SMOW (δD, $\delta^{18}O$ = 0 ‰). Note the large enrichment in $HD^{16}O$. The $H_2{}^{16}O$ abundance is retrieved from the strong line near 3594 cm^{-1}, and its value verified with a weaker $H_2{}^{16}O$ recorded in the CO_2 spectral scan of the same gas sample. Undulations in the single spectrum baseline are discriminated against by the TLS data processing that integrates over the complete individual lines.

This distillative effect can manifest in EGA experiments in at least two ways, the simplest end-member examples of which are: 1) The TLS 'cut' does not encompass the full water release but rather captures a fraction of the sample; 2) The TLS 'cut' captures a mixture of a nearly complete release from one material and either the residue or the initial release from another material. In the first case, the D/H and $^{18}O/^{16}O$ ratios of the water measured by TLS could be artificially increased, decreased, or nearly equal to that of the 'true' ratios corresponding to total extraction depending on what fraction of total extractable water and where along the release curve (e.g., first 10%, middle third, last 10%) it is collected. Quantifying the magnitude and direction of fractionation would require considerable experimental and analytical work on various Mars soil simulants and mineral mixtures to determine 'how much' (i.e., what fraction of total water recovered) is sufficiently representative of the bulk and is thus beyond the scope of this work.

Per (2), the D/H and $^{18}O/^{16}O$ isotopic ratios measured by TLS would be artificially increased in the case where a high-temperature water release (e.g., phyllosilicate dehydroxylation) mixes with the release of heavy-isotope-enriched residual water from a partial extraction at lower temperatures. Similarly, the isotope ratios would be artificially decreased if water released at low temperatures mixed with the very first 'whiff' of water released from a different mineral at a higher temperature. Results from laboratory-based testbed experiments indicate that this 'phase mixing' exerts a larger control than distillative effects on the measured isotopic composition of evolved gases. Given that the water analyzed by the TLS is almost always a mixture of mineralogical sources, 'phase mixing' almost surely affects the isotopic compositions presented in **Table S1**. Except for later experiments where the TLS cut occurred over intermediate temperature ranges (i.e., Glasgow through Nontron), SAM-TLS analyses targeting clay dehydroxylation effectively isolated clay-mineral-derived hydroxyl water for isotopic analysis. We therefore assume in our discussions of those samples that any other contributions are negligible and thus have had little to no effect on the isotopic compositions as measured.

There are two other general categories of complications to consider: sample effects other than mixing and instrument effects. Here, "sample effects" refer to other possible sources of water that can be traced back to either minerals in the sample itself or reactions between and/or among other species released by the sample during the thermal ramp. For example, intra-mineral exchange of oxygen and/or hydrogen isotopes between different 'types' of water within a mineral (e.g., molecular interlayer water and hydroxyl groups) or exchange of oxygen isotopes between framework oxygen atoms and oxygen atoms in dehydroxylating or dehydrating water is a plausible mechanism for modification of the measured water's isotopic composition (131-133). The magnitude and direction of such fractionation in the case of oxygen isotopes is a function of the mineral(s) involved and the

extent of exchange—both of which are temperature-dependent over a range that may not coincide with the TLS 'cut'—and thus potential effects of such a process cannot be predicted *a priori* for our dataset.

Other sample effects could include the formation of H_2O via the combination of O_2 and H_2 released from other sources with significantly different hydrogen and/or oxygen isotopic compositions (such as decomposition of chlorate or perchlorate salts (134) and pyrolysis of refractory organics (115), respectively). Although highly exothermic, the trimolecular, collision-driven, gas-phase combination reaction of H_2 and O_2 to produce H_2O ($2\ H_2 + O_2 \rightarrow H_2O$) is statistically unlikely to occur inside SAM, let alone at abundances high enough to affect the isotopic composition of mineral-evolved water.

Given the concomitant evolution of CO_2 during many of the EGA runs, oxygen isotope exchange between CO_2 and water vapor within the instrument must also be considered. Under ambient conditions, the equilibrium isotopic fractionation between the two species is large (~45‰), but the kinetics of the reaction inhibit vapor-vapor oxygen isotope exchange. The higher temperatures of the EGA experiments may facilitate exchange; however, at such temperatures, the magnitude of fractionation decreases significantly, e.g., $\alpha^{18}_{(\mathrm{CO2,v\text{-}H2O,v})}$ ~1.006 at 300°C (130). The flow-through nature of EGA experiments (i.e., evolved gas is rapidly carried away from the oven in a He stream to the two SAM instruments) would seem to obviate this problem; however, marginal exchange could still occur in the combustion experiments described by Stern et al. (135) in which the evolved gases remained in a closed, small-volume system at temperatures above 550°C for 25 minutes. As shown in **Figure S6a**, within the culled dataset presented here, there is no statistical correlation between the oxygen isotopic compositions of evolved CO_2 and that of evolved H_2O sampled and measured by the TLS over the same temperature 'cut': linear least squares fits weighted to errors in both variables yield goodness of fit parameters R^2 ~ 7.62e–3 (p-value of 7.71e–8) and R^2 ~ 5.05e–4 (p-value of 7.52e–2) with and without the Cumberland data, respectively.

There is, however, an apparent linear correlation between the oxygen isotopic compositions of CO_2 and H_2O evolved from the Cumberland subsamples (**Figure S6b**). The CO_2 releases from all Cumberland subsamples peaked at temperatures <300°C and thus may correspond in part to thermal decomposition of carbonates. However, some of the CO_2 produced during pyrolysis of the Cumberland rock powders is thought to derive from (partial) combustion of the derivatizing agent *N*-methyl-*N*-(*tert*-butyldimethylsilyl)-trifluoroacetamide (MTBSTFA) vapor and its reaction products in the presence of O_2 released during perchlorate and/or chlorate decomposition (134). This assertion is based in part on the observations of concurrent low-temperature (~125–300°C) release of CO_2, O_2, HCl, chlorinated hydrocarbons, and a known reaction product between MTBSTFA and H_2O, i.e., 1,3-bis(1,1-dimethylethyl)-1,1,3,3-tetramethyldisiloxane—as shown in the EGA trace for CB2 (134). This assertion is also based on work by (136), who postulate that O_2 from perchlorate decomposition + carbon from partial combustion of MTBSTFA (and its byproducts) could lead to the generation of CO_2 observed in SAM-EGA of Rocknest samples pyrolyzed above 200°C. Thus, it is plausible that reactions between MTBSTFA and other evolved volatiles have contributed to an increase in evolved CO_2 abundances in some of the Cumberland SAM-EGA experiments as well. Although these reactions can affect the $\delta^{13}C$ value of, e.g., CO_2 and CH_4 (137, 138), the direction and magnitude of any effects on the oxygen isotopic compositions of, e.g., CO_2 and H_2O has not been quantified.

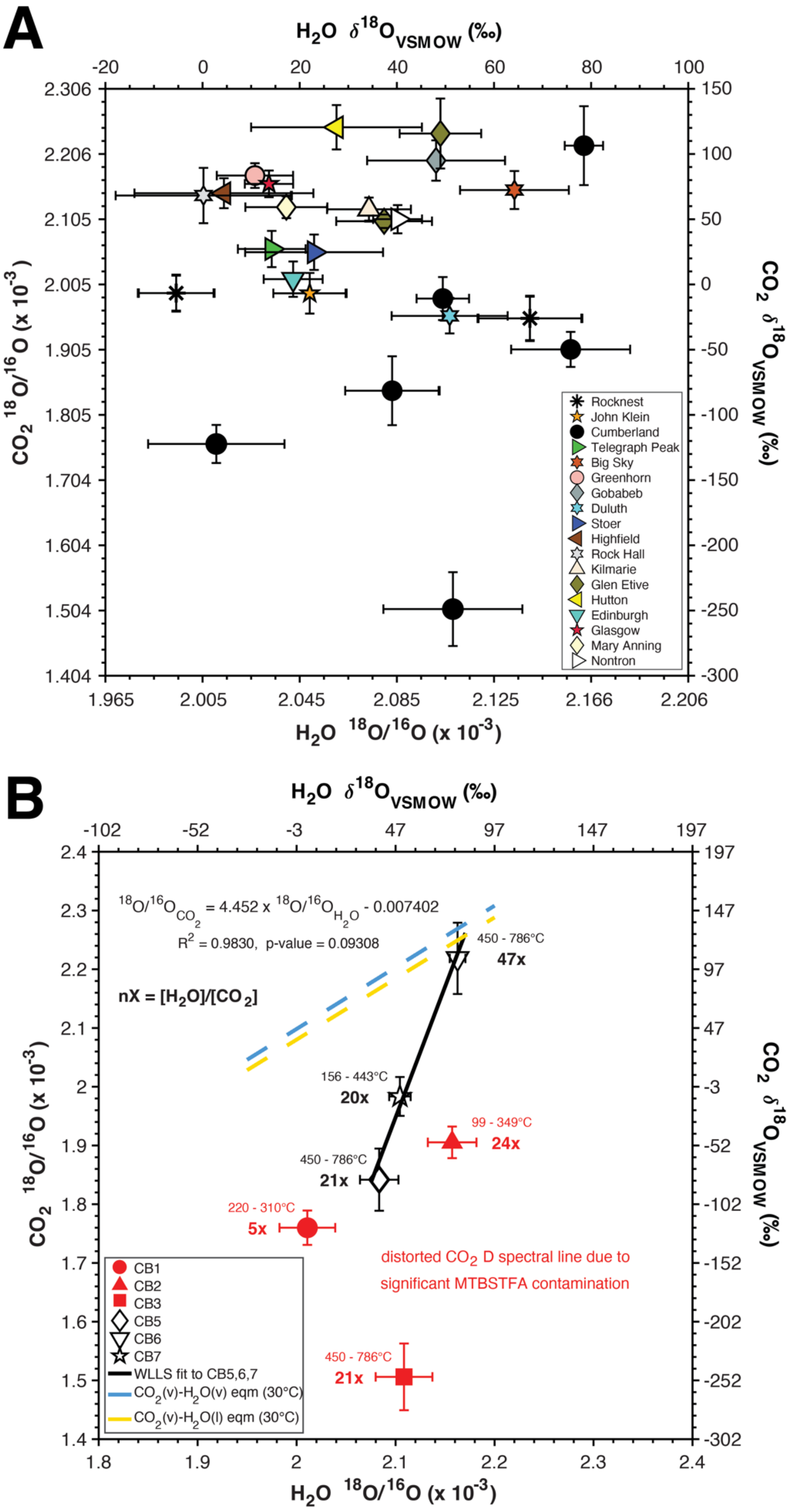
A
H_2O $\delta^{18}O_{VSMOW}$ (‰)
CO_2 $^{18}O/^{16}O$ (x 10^{-3})
CO_2 $\delta^{18}O_{VSMOW}$ (‰)
H_2O $^{18}O/^{16}O$ (x 10^{-3})
Rocknest
John Klein
Cumberland
Telegraph Peak
Big Sky
Greenhorn
Gobabeb
Duluth
Stoer
Highfield
Rock Hall
Kilmarie
Glen Etive
Hutton
Edinburgh
Glasgow
Mary Anning
Nontron
B
H_2O $\delta^{18}O_{VSMOW}$ (‰)
$^{18}O/^{16}O_{CO_2}$ = 4.452 x $^{18}O/^{16}O_{H_2O}$ - 0.007402
R^2 = 0.9830, p-value = 0.09308
nX = $[H_2O]/[CO_2]$
450 - 786°C
47x
156 - 443°C
20x
99 - 349°C
24x
450 - 786°C
21x
220 - 310°C
5x
distorted CO_2 D spectral line due to
significant MTBSTFA contamination
450 - 786°C
21x
CB1
CB2
CB3
CB5
CB6
CB7
WLLS fit to CB5,6,7
CO_2(v)-H_2O(v) eqm (30°C)
CO_2(v)-H_2O(l) eqm (30°C)
CO_2 $^{18}O/^{16}O$ (x 10^{-3})
CO_2 $\delta^{18}O_{VSMOW}$ (‰)
H_2O $^{18}O/^{16}O$ (x 10^{-3})

Figure S6 (previous page). (A) TLS measurements of the oxygen isotopic compositions of carbon dioxide and water evolved over the same temperature 'cut' for samples from the culled dataset. Because TLS CO_2 EGA data are unavailable for the Confidence Hills, Mojave, Oudam, and Marimba samples, these have also been omitted from the figure. **(B)** Apparent correlation between the oxygen isotopic compositions of evolved CO_2 and H_2O from a subset of Cumberland subsamples. Symbols in red correspond to samples showing distorted D lines (CO_2) in the TLS spectra; such distortions are interpreted to reflect significant MTBSTFA contamination. CB1, CB2, and CB3 are thus not included in the York-type weighted linear least squares fit to the Cumberland data (black line and corresponding regression equation). Bolded numbers adjacent to each symbol correspond to the ratio of evolved H_2O/CO_2, each in nanomoles: e.g., 5x = ~5 times more H_2O (nanomoles) than CO_2 (nanomoles) evolved over the temperature cut sent to the TLS. Temperature cuts are given as a range adjacent to the corresponding subsample. Blue and yellow dashed lines correspond to the expected CO_2 $^{18}O/^{16}O$ isotopic ratio assuming isotopic equilibrium between CO_2 vapor and H_2O vapor (blue) or CO_2 vapor and H_2O liquid (yellow) at 30°C. Error bars correspond to two standard error of the mean.

Evolved H_2O can react with the MTBSTFA derivatizing agent to make monosilyated (MSW) and/or bisilyated water (BSW). BSW concentrations have been used as a proxy for the degree of background MTBSTFA contamination in the SAM instrument suite in multiple studies, including investigations into the carbon isotopic compositions of evolved CH_4 (137, 138). Similarly, H_2O can react with Cl_2 gas generated during thermal decomposition of perchlorate and/or chlorate salts to generate HCl, which in turn can react with C to generate chlorinated hydrocarbons (136, 139, 140). All these products are present in the Cumberland mass spectra.

Although the carbon dioxide spectral lines are clearly affected by the presence of MTBSTFA and/or its reaction byproducts, we observe no comparable effect on the TLS's H_2O absorption spectra. For MTBSTFA + evolved water reactions to demonstrably alter the 'true' oxygen isotopic composition of evolved water, either the reactions would need to be extremely fractionating OR the abundances of the MSW and BSW would need to correspond to loss of a significant fraction of the total evolved water relative to that measured by the TLS. Fractionation factors for MTBSTFA + water reactions are presently unknown, and their determination is beyond the scope of this study. To the latter point, however, TLS water abundances measure in the thousands to tens of thousands of nanomoles (**Table S1**; **Figure S7**), whereas MSW and/or BSW are present at ~10s to ~100 nanomoles in SAM EGA experiments (e.g., (134)). Five of the six Cumberland evolved water measurements have $\delta^{18}O_{VSMOW}$ values within two standard error of one another (**Figure S7**). This observation is particularly noteworthy for subsamples CB2 (a low-temperature release) and CB3 (a high-temperature release): their CO_2 IR spectra show significantly distorted D lines, but their H_2O IR spectra show no comparable aberrations. Although circumstantial, these observations suggest that the reactions between MTBSTFA and other volatiles, which occur primarily at temperatures <300°C (136, 139, 140), may not significantly modify the isotopic composition of evolved water regardless of temperature release.

In contrast to sample effects, "instrument effects" include processes and factors internal to the SAM instrument suite that could modify or contribute to modification of the evolved water's isotopic composition during an EGA experiment. As shown in the SAM gas flow diagram (Figure 3 in (141)), the TLS is positioned 'downstream' from the pyrolysis ovens and the QMS, such that the evolved gases delivered to the TLS have traveled through multiple valves and transfer lines; thus, it is conceivable that the water vapor has undergone diffusive fractionation by the time it reaches the TLS. Isotopic fractionation via diffusion would lead to preferential enrichment in the light isotopologue

($H_2{}^{16}O$) due to its lower mass and faster relative diffusivity, which would manifest as D/H and $^{18}O/^{16}O$ ratios lower than the 'true' martian values. However, as described in the **Methods** section of the main text, those transfer lines are maintained at a constant 135°C and the evolved volatiles are lofted in a He carrier gas at a constant flow rate throughout the experiment, which should negate any such effects as the gas is no longer within a diffusive regime.

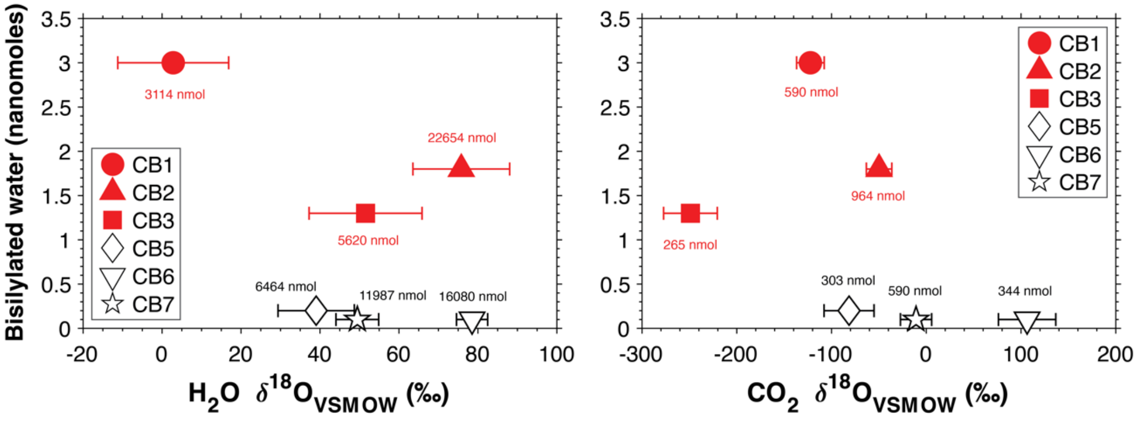

Figure S7. TLS oxygen isotopic compositions of evolved CO_2 and H_2O from Cumberland subsamples compared to the amount of bisilyated water (BSW) present in the SAM instrument. BSW abundances (given on the y-axis) are presented as a proxy for the degree of MTBSTFA contamination within SAM; these abundances (in nanomoles) for CB1 through CB6 were calculated based on QMS data and first reported in (137). We estimate a comparable BSW concentration for CB7 based on (1) the decrease in BSW as a function of time and (2) the fact that the CB7 EGA experiment underwent the same thermal pre-treatment as CB6. Filled red symbols correspond to subsamples whose TLS CO_2 spectra contain distorted D lines—interpreted as evidence for significant MTBSTFA contamination. Open symbols with black outlines correspond to experiments in which the sample cup was thermally pre-treated to drive off MTBSTFA and DMF (dimethylformamide) contaminants prior to filling the cup with rock powder. Annotations adjacent to each symbol correspond to the nanomoles of either H_2O (left panel) or CO_2 (right panel) present in the TLS Herriott cell during each subsample analysis.

Could there be unidentified gaseous 'contaminants' within the TLS Herriott cell whose presence affects the water isotopologue spectra and thus the retrieved isotope ratios? The TLS spectral region of 3593.2 to 3594.4 cm^{-1} (2.7830 to 2.7821 μm) includes two spectral lines from each of the $H_2{}^{16}O$, $HD^{16}O$ and $H_2{}^{18}O$ species (**Figure S5**). Isotopic delta values calculated from each pair of lines agree well within error, but for the analyses presented in this manuscript we use only the fully-isolated lines at 3593.6 cm^{-1} for $HD^{16}O$, 3593.85 cm^{-1} for $H_2{}^{16}O$, and 3594.08 cm^{-1} for $H_2{}^{18}O$. Nearby multiple CO_2 lines are interleaved with the water lines across this scan and provide useful relative abundance verification but show no interference. In general, water evolves from the samples in significant amounts, so that the water lines dominate the recorded spectra at high signal-to-noise ratios, with no evidence seen of unknown or interfering lines. Typical line-center depths for $HD^{16}O$ lines, for example, are 2–10% equivalent absorption levels, compared to values of <0.01% for underlying optical interference fringes. Absence of contributions from possible interfering lines is further verified in the individual line shapes of $H_2{}^{16}O$, $HD^{16}O$, and $H_2{}^{18}O$ that show no measurable deviation from HITRAN predictions at the Doppler limit that might otherwise present as shoulders or perturbations. The likelihood

that contaminant spectral lines are exactly at the same line center is very low, since for triatomic or larger molecules—such as those produced in reactions with the MTBSTFA derivatizing agent—additional lines would be expected to also be seen across the relatively-wide spectral region but are not. Although reactions between martian materials and MTBSTFA can produce methane in the TLS 3.27-µm region, we have not seen unexpected lines in the 2.78-µm water region at levels that would produce significant error in our water isotope delta value retrievals. Regardless, laboratory-derived spectral line surveys of MTBSTFA and its relevant reaction products within the SAM-TLS spectral regions could resolve any outstanding concerns regarding potential interferences.

Residual water adsorbed to internal walls, transfer lines, or other instrument components that 'see' the evolved gas during an EGA experiment is the most likely instrument-derived source of isotopic 'contamination' to the water ultimately measured by the TLS; however, degassing of other species (e.g., H_2 embedded in metal) at high temperatures could also confound the isotopic signature as measured given the possibility of 'downstream' exchange or reactions between evolved gases. Both (114) and (142) identified terrestrial hydrogen sources in blank cup runs performed prior to EGA experiments on Rocknest and Cumberland subsamples, respectively. Because the hydrogen isotopic composition of martian water contaminated by such sources could be artificially lowered, the D/H ratios reported in both papers required corrections based on the abundance and D/H ratio of the contaminant. Based on the range of oxygen isotope ratios measured by the TLS (**Table S1**) compared to the range generally observed for terrestrial water (**Figure S1**), the oxygen isotopic composition of martian water, too, could be artificially lowered via terrestrial contamination. To first order, the effect of residual adsorbed martian water (i.e., from a previous experiment) to the isotopic composition of evolved water measured by the TLS should be less than that of terrestrial water, but the magnitude of the effect will still depend on the relative abundance of the contaminant to the total water seen by TLS and on their relative isotope ratios.

A combination of blank experiments and additional heating and purging of the instruments should minimize such 'contaminant' effects. However, as previously noted, running blanks prior to each EGA experiment is infeasible and applying a blanket background correction from one blank to multiple later EGA experiments is not scientifically appropriate. Instead, a "preconditioning" run is conducted prior to each sample analysis to clean the instrument in preparation for the next sample. In a preconditioning run, gas lines are heated to the same temperature as during a sample run, and the oven is heated to roughly the same temperature as during a sample run. All of this occurs with the pumps on to remove any residual or evolved gas. Unlike regular sample runs, the preconditioning run does not include the use of the He carrier gas; however, the low pressure and heated lines were demonstrated to be effective at preventing most cross-contamination between runs in support of this resource-saving decision.

Accuracy of the HITRAN-based approach for calculating the oxygen isotopic compositions of ^{18}O-enriched waters

As illustrated in **Figure S1**, the majority of Gale crater evolved water samples are significantly ^{18}O-enriched relative to terrestrial materials. Although the relative absorptions of isotopologue spectral lines measured by SAM-TLS were calibrated pre-launch, the water calibration consisted of a single NOAA-certified standard: 'Boulder water', which,

per the Supplement to (39), had a δD_{VSMOW} value of –110.11 ± 0.05‰ (1 SD) and a $\delta^{18}O_{VSMOW}$ value of –14.91 ± 0.05‰ (1 SD). SAM-TLS data processing involves the use of calibration multipliers for comparison to HITRAN line strengths, but the HITRAN 2016 database (143) does not include line intensities for water that is as ^{18}O- and D-enriched as that measured in the EGA of martian mineralogical water samples from Gale. To address potential complications in Mars water D/H isotope ratio retrievals associated with this discrepancy, (144) determined rovibrational line strengths as well as self- and He-broadening coefficients for the water isotopologues $H_2{}^{16}O$, $H_2{}^{18}O$, and $HD^{16}O$ using different samples of Boulder water with independently verified isotopic compositions (δD_{VSMOW} = –120 ± 0.4‰ (1 SD) and $\delta^{18}O_{VSMOW}$ = –16.2 ± 0.36‰ (1 SD)) and a deuterated variant, "δD-enriched Boulder water" (δD_{VSMOW} = 1171.6 ± 0.4‰ (1 SD) and $\delta^{18}O_{VSMOW}$ = –16.6 ± 0.61‰ (1 SD)). Those laboratory experiments were undertaken because foreign-broadening by He—not accounted for in the HITRAN-12 database used in the initial SAM-TLS data processing—can affect observed isotopologue linewidths and thus modify the retrieved isotope ratios.

With appropriate experimentally derived corrections, (144) showed that that they could retrieve accurate and precise D/H ratios for the isotopically normal Boulder water using either the HITRAN-based approach (as described in (39, 142) and the main text) or calculated directly from experimental data. Results from the D-enriched Boulder water are less accurate: the experimentally derived isotopic composition of the D-enriched Boulder water was still within two standard error of its 'true' δD value, while the HITRAN-retrieved results were within ~3 standard error (144). Results from Table 8 in (144) indicate that D/H isotope ratios retrieved using the HITRAN-based approach versus that calculated directly from experimental data differ by ~10‰ at δD_{VSMOW} values of ~1200‰; this offset increases to ~60‰ for δD_{VSMOW} values close to 3500‰—i.e., martian water isotopic compositions. Offsets of ~60‰ are comparable to the largest 2 SE uncertainties on δD values from our Gale crater EGA dataset (**Table S1**). Although (144) also report a ~1.8‰ offset between water $\delta^{18}O$ values calculated via the HITRAN-based approach versus that calculated directly from experimental results, this offset applies to water with a terrestrial $\delta^{18}O_{VSMOW}$ value of approximately –16.2‰. We therefore undertook a series of laboratory experiments using a benchtop TLS comparable—but not identical—to the SAM-TLS to assess the validity of the HITRAN approach for calculating the $\delta^{18}O_{VSMOW}$ values of water with ^{18}O enrichments spanning the values in our Gale crater EGA dataset.

An ^{18}O-enriched water sample ('SEW'; $\delta^{18}O_{VSMOW}$ of approximately 110‰) was created via addition of ~100 microliters of 10 atom% ^{18}O water (Aldrich Chemistry, Lot #MBBB2989V) to ~40 mL of 18Ω Milli-Q water, which was assumed to have an isotopic composition comparable to that of Pasadena, California tap water, i.e., $\delta^{18}O_{VSMOW}$ of approximately –9‰ (145). Aliquots of SEW and Pasadena DI water were set aside for analysis as endmember isotopic compositions. Different proportions of SEW and Pasadena DI water were mixed volumetrically to create four additional samples with different degrees of ^{18}O enrichment (**Table S3**). An aliquot of evaporatively enriched water ('eBOC'; $\delta^{18}O_{VSMOW}$ of approximately 25‰) previously prepared in the Caltech Laboratories for Stable Isotope Geochemistry was set aside for analysis, as was a 70:30 (v/v) mixture of eBOC and Pasadena DI water. To minimize headspace (and associated isotopic fractionation due to vapor pressure isotope effects), ~2 mL from each of the eight

samples were transferred into individual 2 mL GC vials and capped prior to isotopic analysis via a commercial cavity ring-down spectrometer. A duplicate set of samples was prepared for single-blind isotopic analysis using a JPL benchtop TLS equipped with a 2.78 μm laser, Versal electronics system, and new collimating lens.

Samples were analyzed at Caltech's Resnick Water and Environment Lab (WEL) using a PICARRO L2140-i Isotopic Water Analyzer attached to an A0211 High-Precision Vaporization Module during a single analytical session over a 24-hour period from 13–14 April 2023. Four WEL natural water working standards with known isotopic compositions ($\delta^{18}O_{VSMOW}$ given in parentheses ± 1 SD) were used as internal calibrants during the PICARRO analyses: GNP17 (–16.8 ± 0.1‰), CIT17 (–9.8 ± 0.1‰), MAUI (–3.2 ± 0.1‰), and DeepBlue (1.5 ± 0.2‰). All four working standards' isotopic compositions were calibrated against NIST reference materials VSMOW, GISP, and VSLAP in 2020. In our April 2023 analytical session, working standards were analyzed in order of increasing $\delta^{18}O_{VSMOW}$ values prior to sample analyses and then in order of decreasing $\delta^{18}O_{VSMOW}$ values following sample analyses. The samples described in the preceding paragraph (including our 18Ω MilliQ water endmember) were also analyzed in order of increasing ^{18}O-enrichment. The full analytical session was bracketed by analyses of WEL MilliQ water. Each analysis consisted of nine 2-μL water sample (or standard) injections using an autosampler and syringe rinsed three times in WEL MilliQ water prior to each injection and then rinsed another two times after each injection. Preliminary data processing to calculate mean D/H, mean $^{18}O/^{16}O$, and associated standard deviations for each injection was performed using an in-house script; the resulting dataframe was then exported to a .csv file for manual inspection and further data analysis.

There are no NIST water standards or WEL working standards with $\delta^{18}O_{VSMOW}$ values comparable to our most-^{18}O-enriched sample compositions, largely because terrestrial waters rarely reach isotopic enrichments greater than +20‰. This mismatch leads to greater measurement uncertainties on our most ^{18}O-enriched water samples, but—notably—the corrected and calibrated PICARRO results are still within uncertainty of the expected (calculated) $\delta^{18}O_{VSMOW}$ values for those same samples (**Table S3**). PICARRO data were post-processed—including drift correction and calibration to working standards—using the FLIIMP software (146). The CIT17 and MAUI working standards were used for drift correction while the GNP17 and DeepBlue working standards were used to calibrate the sample results according to IAEA recommendations. FLIIMP calculates a combined uncertainty from the square root of the squared sum of all error components, including the assigned uncertainty of the calibration standards, the measurement uncertainty of the calibration standards, and the long-term measurement reproducibility and repeatability (146). We then used the vapor pressure isotope effect (VPIE) expression from (147) (see **Table S6**) to calculate the $^{18}O/^{16}O$ ratio of water vapor in isotopic equilibrium with each liquid water sample at a temperature of 25°C. These data are given in the 'Calculated Vapor' column and are directly comparable to the TLS 'Measured Vapor' data in the right-most column of **Table S3**.

Immediately prior to the TLS laboratory testbed analyses, aliquots of each water sample were transferred via pipette from the sealed 2 mL GC vials into custom blown-glass sample vials consisting of an ~3-cm-long hollow glass 'finger' separated from an access port via a Teflon / PTFE stopper. These sample fingers were connected via the access port to an inlet on the laboratory testbed TLS, the Teflon stopper opened, and the vapor-liquid

system allowed to equilibrate (i.e., reach a stable vapor pressure) at 25°C. The testbed TLS Herriot cell was then filled with ~5 mbar of the headspace vapor and direct absorption spectra were collected between 3594.3 and 3593.5 cm^{-1} for a minimum of 30 minutes (corresponding to ~20 data points). There are five water isotopologue spectral lines within the measurement region: $H_2{}^{16}O$ (parent) lines C and D, $H_2{}^{18}O$ lines B and F, and HDO line E. Although $\delta^{18}O$ values are calculated using only the B line for *Curiosity* SAM-TLS data, here we calculated $\delta^{18}O$ values from both the B and F lines (using the D-line values for the denominator when evaluating isotope ratios).

As described above, numerical multipliers are used to adjust the HITRAN line strengths prior to retrieval of isotope ratios in the form of delta values. At lower vapor pressures, the multiplier for the D parent line (1.02) is determined by comparison with the C parent line (multiplier fixed at 1), so that the D line can be used in isotope ratio calculations at higher vapor pressures (~5 mbar) where the signal-to-noise ratio on the weaker HDO and $H_2{}^{18}O$ lines is greater. Initial isotope ratios were calculated by averaging over all data points for a given sample and assuming multipliers of 1 for all lines except the D line. A sample of Boulder water, with a δD_{VSMOW} value of –197‰ and a $\delta^{18}O_{VSMOW}$ value of –25‰, was used as an internal calibration target for setting the B-, E-, and F-line multipliers to values of 1.011, 0.980, and 1.054, respectively. Each sample's $\delta^{18}O_{VSMOW}$ and δD_{VSMOW} values were then adjusted as follows: $\delta^{18}O_{new,spectral\ line} = \delta^{18}O_{initial,spectral\ line} \times Multiplier_{spectral\ line} + (Multiplier_{spectral\ line} - 1) \times 1000$. Initial standard errors on each retrieved delta value were also adjusted using the corresponding spectral line multiplier. Final $\delta^{18}O_{VSMOW}$ values for each unknown were calculated by averaging the adjusted $\delta^{18}O_{new,B}$ and $\delta^{18}O_{new,F}$ values. Adjusted standard errors were added together in quadrature. These average values are reported in the right-most column of **Table S3**.

In **Figure S8**, we plot the results of our TLS testbed water vapor measurements against the calculated vapor $\delta^{18}O_{VSMOW}$ values in equilibrium with the PICARRO corrected and calibrated liquid results (**Table S3**). In general, the two measurements show good agreement: the median offset between the true (PICARRO) values and the average TLS testbed (HITRAN-derived) values is ~7‰, which is comparable to the precision on the SAM-TLS $\delta^{18}O$ values given in **Table S1** (i.e., a median uncertainty of ~10‰ at the level of two standard error). The largest discrepancy between the PICARRO results and the corresponding TLS testbed analyses (corresponding to an offset of ~37‰) is for the most-^{18}O-enriched unknown water sample, 'SEW'. This unknown is ~110‰ more enriched in ^{18}O than our most enriched PICARRO working standard and ~135‰ more enriched in ^{18}O than the single point calibration used in the TLS testbed analyses, and thus most likely to be affected by errors in linear extrapolation. SEW is also ~30‰ more enriched in ^{18}O than the most-enriched Gale crater evolved water (CB6, which has a $\delta^{18}O_{VSMOW}$ of 79‰; **Table S1**). This contrasts with our second-most-^{18}O-enriched unknown (20:80 DI:SEW; **Table S3**), which not only has a coexisting vapor $\delta^{18}O$ value comparable to that of CB6 but also falls within error of the 1:1 line on **Figure S8**. Given the preceding information, we have chosen to omit SEW from the Deming regression, which we present in **Figure S8** as a quantitative metric for assessing the accuracy of the HITRAN-based $\delta^{18}O$ values. However, we note that a 30+ per mille discrepancy between the true $\delta^{18}O$ value and that calculated from the TLS testbed spectra suggests that using the HITRAN-based approach

may lead to significant overestimates in oxygen isotopic compositions when the true $\delta^{18}O_{VSMOW}$ values are greater than ~80‰.

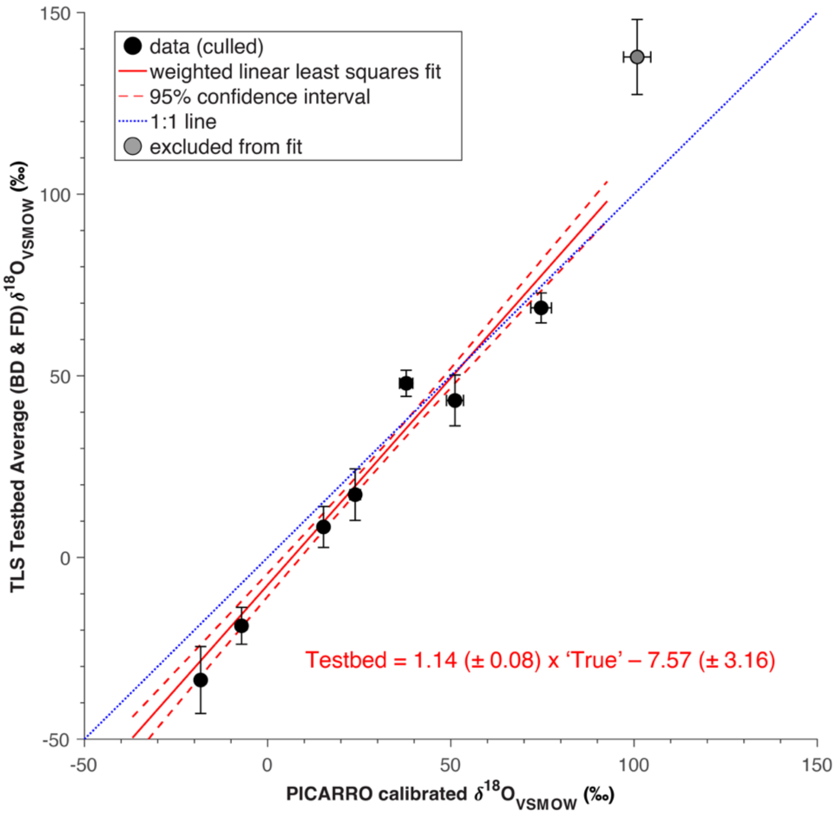

Figure S8. Comparison of JPL testbed TLS and commercial PICARRO water isotope analyzer results for analyses of the same ^{18}O-enriched samples. The dotted blue 1:1 line represents perfect agreement between the two measurements. The solid red line corresponds to a Deming (linear) fit to the solid black circles accounting for errors in both variables—in this case, the measurement uncertainties; the regression expression includes 1σ uncertainties calculated using a Monte Carlo approach (148). Uncertainties on individual data points correspond to two standard error. Data from **Table S3**.

SUPPLEMENTARY METHODS

Numerical Modeling I. H_2O–CO_2 oxygen isotope exchange

In this section, we summarize the calculations used to determine the timescale for complete oxygen isotope exchange between atmospheric carbon dioxide and lake water under Mars-like conditions. These calculations—and nomenclature—are based on those from the 'stagnant film' model implemented in chemical oceanography to determine the timescale for equilibration between atmospheric CO_2 and CO_2 dissolved in a body of water (i.e., the residence time of CO_2 in that body of water), which we then couple to the relative abundance of CO_2 to H_2O in that body of water. Full derivations and detailed explanations of the model can be found in (149).

As its name implies, the stagnant film model of gas exchange across a boundary layer ignores the effects that turbulence at the air-water interface play in facilitating gas exchange. Rather, the model is divided into four boxes: a well-mixed atmospheric layer; an atmospheric boundary layer within which gas-phase diffusion to and from the air-water interface governs transport to the interface; a water boundary layer just below the interface within which diffusion in the liquid phase governs gas transfer; and a well-mixed liquid layer. The stagnant film model proposes that the time it takes for a gaseous species to cross the air-water interface is governed by the diffusivities of this gas through the two 'stagnant' boundary layers.

In the absence of sources or sinks of the gaseous species of interest in the boundary layers, and ignoring the atmospheric stagnant film—as is commonly done due to the rapid transfer of gases through the atmospheric boundary layer relative to that through the liquid water boundary layer (i.e., >>1000 cm hr^{-1} versus ~10 cm hr^{-1} (150))—gas exchange across the air-water interface can be modeled as:

$$\Phi = -k_w([A]_a - [A]_w) \qquad \textbf{Eqn. S1.}$$

In this expression, the flux, Φ, is given in mmol m^{-2} s^{-1}, the piston velocity (also known as the gas transfer coefficient), k_w, corresponds to the ratio of the molecular diffusivity of gas A in water (ε_w, in units of m^2 s^{-1}) to the thickness of the stagnant film in the water (Δz_w, in m). The term in parentheses represents the concentration difference between gas A in the atmosphere, $[A]_a$, and in the water, $[A]_w$, both in mmol m^{-3}. The temporal rate of change for A in a body of water that exchanges only with the atmosphere can be represented by the differential equation

$$\frac{\partial [A]_w}{\partial t} = -\frac{\partial \Phi}{\partial z} = \frac{k_w}{z_{ml}}([A]_a - [A]_w) \qquad \textbf{Eqn. S2,}$$

in which z_{ml} is the thickness of the mixed layer that exchanges A with the atmosphere. Assuming that the concentration of A in the atmosphere is constant, the general solution to **Equation S2** is an exponential function whose time constant, τ, equals the ratio of the mixed layer depth to the piston velocity. Of note, for CO_2, the residence time calculated in this manner is typically multiplied by an additional factor to account for the fact that CO_2 also needs to equilibrate with the dissolved inorganic carbon (DIC) pool in the mixed layer.

Calculation variables and parameter space

The following variables were used to calculate the residence time of CO_2 in a hypothetical lake in Gale crater: system temperature (T), total atmospheric pressure (P), atmospheric partial pressure of CO_2 (pCO_2), lake salinity (S_{salt}), the lake mixed-layer depth (z_{ml}), the volumetric solubility function for CO_2 in water (F_{CO2}) and the saturation vapor pressure of water (pH_2O_{sat}). These last two variables were used to calculate the CO_2 solubility parameter (S_{CO2}). The water stagnant film boundary layer thickness (Δz_w) and the molecular diffusivity of CO_2 in water (ε_w) were used to calculate the piston velocity, k_w. We lay out our assumptions that bound the explored parameter space and describe the variables below. Numerical values used in the model are given in **Table S4**. Note that the units given in **Table S4** are those common to the chemical oceanography literature and must be converted for use in the equations.

Model assumptions, rationale, and implementation

Numerical values for system temperature, total atmospheric pressure and CO_2 partial pressure, water salinity, and lake mixed-layer depth were chosen to represent bounding cases relevant to putative conditions at Gale crater based on Mars atmospheric and paleoclimate models (e.g., (151-159)), geomorphological data from orbital imagery (e.g., (70, 151)), and geochemical and mineralogical data collected by instruments on the *Curiosity* rover (e.g., (105, 107, 108, 110, 160, 161)). The volumetric solubility function for CO_2 in water (F_{CO2}) is an empirically derived function of system temperature and water salinity. The equation and coefficients used in calculating F_{CO2} include corrections for the non-ideality of carbon dioxide (149). The CO_2 solubility parameter (S_{CO2}) was calculated for the 'moist air' case: air at the air-water interface was assumed to be water-saturated, and thus the partial pressure of H_2O (corresponding to the saturation vapor pressure at system temperature) was used along with the total system pressure to convert F_{CO2} to S_{CO2}.

Implementation of the stagnant film model—particularly in the calculation of CO_2 piston velocity (k_w)—requires definition of the film thickness at the surface of the water body. This 'thickness' varies in terrestrial waters as a function of surface agitation due to wind and wave activity (e.g., (162)). For simplicity, we performed calculations using three film-thickness bounding cases: 30 microns, which corresponds to the approximate mean value across terrestrial oceans; 100 microns, which corresponds to the minimum film thickness determined experimentally for CO_2 in an agitated freshwater system (representing wind and wave action); and 300 microns, which corresponds to the maximum thickness for the same, albeit in sea water (162). The maximum film thickness in a low-agitation freshwater system and the minimum film thickness in a high-agitation sea water system fall within this 100–300 micron range (162). Values for the self-diffusion coefficient of molecular CO_2 dissolved in water (ε_w) were calculated based on the empirical fit to molecular dynamics simulation results given in (163). We chose to use these data for three reasons. First, simulations were conducted over a range of temperatures down to 273 K, whereas experimental and observational studies performed to-date under ambient pressure conditions bottom-out at 278 K (164). Second, the MD results are within error of observational and experimental data taken over our temperature range of interest, 278–293 K. Finally, the simulations were performed for idealized systems and thus—although subject to errors associated with molecular dynamics simulations—are independent of the vagaries associated with different experimental methods and analytical techniques.

Per **Equation S2** and assuming a constant atmospheric pCO_2, we calculated the fundamental time constant, τ, for the complete physical exchange of dissolved CO_2 molecules for atmospheric CO_2 molecules. As noted in our presentation of **Equation S2** above, $\tau = z_{ml} / k_w$. In a well-mixed water column, the mixed layer depth (z_{ml}) corresponds to that region over which no thermal or chemical gradients exist. Mineralogical and geochemical evidence at Gale suggest that, at least over part of the crater's hydrologic history, standing lakes were redox-stratified (107). In our simplified model, however, the mixed layer depth represents the water column depth within which air-water gas exchange, evaporation, and carbonate chemistry affect the oxygen isotope budget. As a result of this simplification, our residence time calculations do not account for timescale(s) associated with lake turnover. A more physically realistic model that incorporates seasonal (and perhaps even diurnal) turnover is beyond the scope of this work. Within our simplified model, we use orbital (major lake stands at ~300-, 400-, and 700-meter depths (70)) and in situ (deltas ~1 to 4 meters thick, requiring standing water of at least the same (72)) observations at Mars in conjunction with the terrestrial lakes database HydroLAKES (https://www.hydrosheds.org/) to place order-of-magnitude bounds on our mixed layer depth of 10, 100, and 1000 meters.

The piston velocity (k_w) is most simply calculated as the ratio of the molecular diffusivity of CO_2 in water (ε_w) to the water stagnant film boundary layer thickness (Δz_w). Many different, more complicated (and hence more realistic) formulations for determining piston velocities exist within the chemical oceanography literature; however, no one expression is favored over another (149). Some of these additional terms and variables include accounting for wind speed, turbulence near the air-water interface, water kinematic viscosities, wave types, bubble formation, gradients in temperature and humidity, and the presence of organic films on the water surface (149). Given all the unknowns surrounding specific conditions at Gale during lake stands, we did not attempt to account for any of these additional variables in our calculations of k_w.

To convert τ (the time required for complete CO_2 molecular exchange) to an oxygen-isotope exchange timescale, we multiplied each case's τ by the ratio of H_2O-contributed oxygen atoms to CO_2-contributed oxygen atoms within the water column. The molarity of pure water (in moles H_2O / L) was determined using the densities of pure water at 273.16 K and 293.15 K (**Table S4**), and these values were then converted to moles of oxygen per cubic meter. The moles of CO_2-contributed oxygen atoms in the water column were calculated using five different initial conditions. Even though only a small amount of dissolved carbon is in the form of CO_2 at circumneutral pH, we assumed for the first case that the total amount of dissolved CO_2 was equal to the mean total DIC in terrestrial oceans: ~3 mM (i.e., 3 moles CO_2 / m^3, corresponding to 6 moles of O from CO_2 per m^3). This value was used in both the 273 K and the 293 K calculations, whereas the four other values were temperature-dependent. In those remaining four cases, we used a solubility-defined application of Henry's Law to calculate the moles of dissolved CO_2 that would be in equilibrium with our defined pCO_2 values at 273 and 293 K; within the parameters of our model (**Table S4**), this calculation is: $[CO_2]_{liq} = S_{CO2} * pCO_2$. We note that many of the CO_2-specific parameters presented in **Table S4** such as S_{CO2} are based on empirical fits to data either collected from or designed to mimic natural terrestrial conditions, where pCO_2 is 365 μatm and the corresponding equilibrium $[CO_2]_{liq}$ would be ~12 μM (i.e., 0.012 moles CO_2 / m^3) at 293 K. As such, we emphasize that the parameters given in **Table S4**, the

application of a Henrian-based equation—which is more suitable for solutions approaching infinite dilution—and our overall results should be taken as order-of-magnitude estimates for comparison across bounding cases rather than definitive, absolute timescales. The isotopic-exchange timescales calculated thusly are plotted in **Figure S9** (with a single subset in **Figure 4** of the main text). Due to the many simplifying assumptions employed, we made no attempt to modify these values based on a dissolved CO_2 to total DIC ratio (i.e., based on carbonate speciation), as is done in terrestrial calculations of the same. We further justify this decision based on the fact that the other potentially rate-limiting processes of CO_2 hydration (seconds to minutes depending on T and pH (165-168)) and carbonate species isotopic equilibrium (~90 hours, (169)) are orders of magnitude faster than the timescale for complete physical 'swapping' of atmospheric CO_2 for dissolved CO_2, calculated as described in the preceding sections.

Figure S9 (next page). Timescales in Earth years for complete oxygen exchange between H_2O in a hypothetical lake of constant depth at Gale and atmospheric CO_2. Values were calculated for the parameter space bounded by variables given in **Table S4**. Colors correspond to the concentration of dissolved CO_2, which was assigned to be either equal to mean terrestrial oceanic DIC (3 mmol/L, blue symbols) or the concentration in equilibrium with a CO_2 partial pressure of either 0.5 atm (~500 mbar; yellow symbols) or 1 atm (~1 bar; orange symbols). Calculations were performed at two salinities: S_{salt} = 0 (freshwater, open symbols) and S_{salt} = 35 (terrestrial oceans, filled symbols). The thickness of the boundary layer (i.e., the stagnant film at the water surface) was either 30, 100, or 300 microns (circles, triangles, or squares, respectively). The well-mixed region of the water column—taken as a proxy for lake depth—is given on the x-axis. Subplots show results for a constant system temperature and total atmospheric pressure.

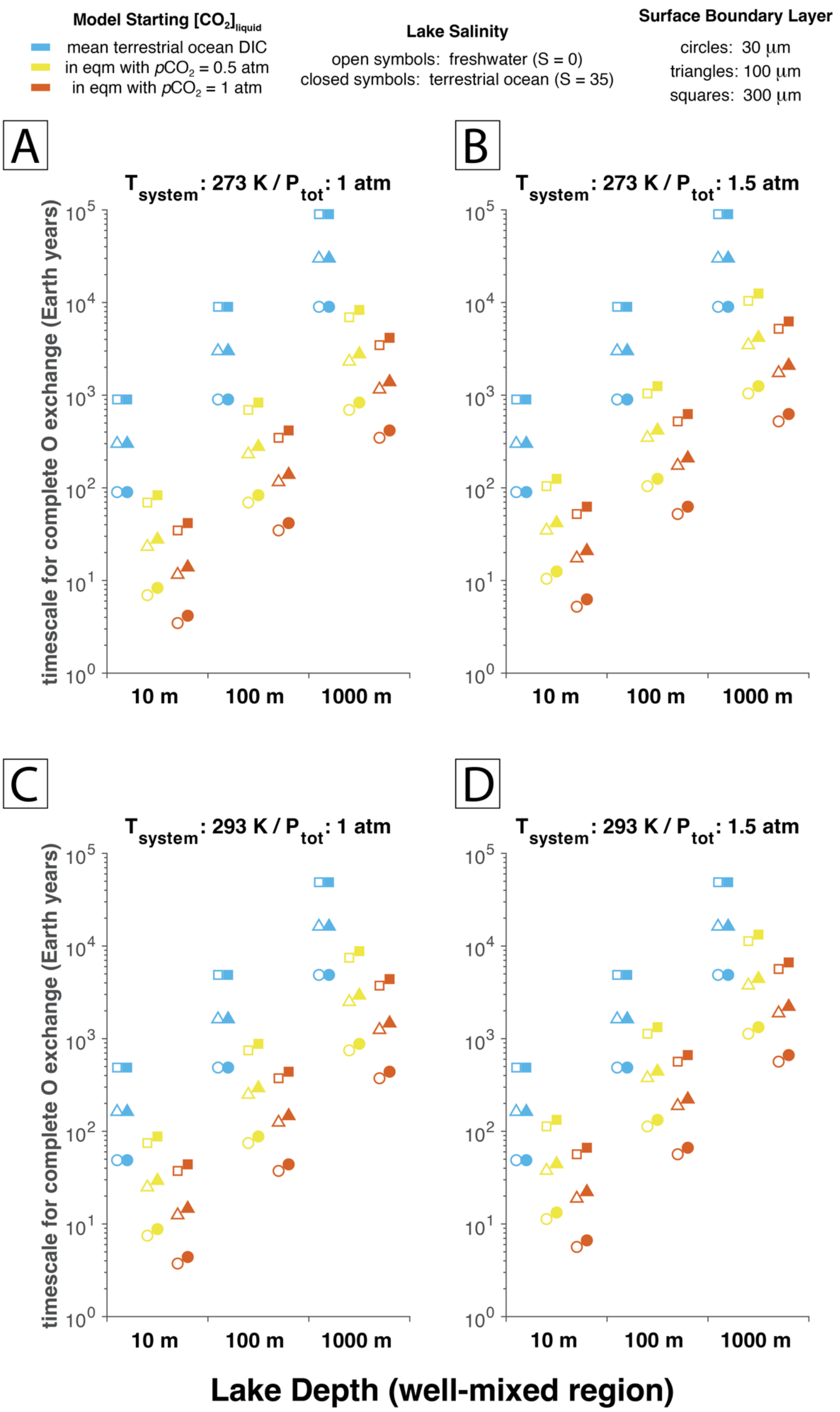
Model Starting $[CO_2]_{liquid}$
mean terrestrial ocean DIC
in eqm with pCO_2 = 0.5 atm
in eqm with pCO_2 = 1 atm
Lake Salinity
open symbols: freshwater (S = 0)
closed symbols: terrestrial ocean (S = 35)
Surface Boundary Layer
circles: 30 μm
triangles: 100 μm
squares: 300 μm
A
T_{system}: 273 K / P_{tot}: 1 atm
B
T_{system}: 273 K / P_{tot}: 1.5 atm
C
T_{system}: 293 K / P_{tot}: 1 atm
D
T_{system}: 293 K / P_{tot}: 1.5 atm
timescale for complete O exchange (Earth years)
10^0
10^1
10^2
10^3
10^4
10^5
10 m
100 m
1000 m
Lake Depth (well-mixed region)

Numerical Modeling II. Linear relationships in isotope ratio space

In the following subsections, we describe the quantitative models used to assess the observed linear relationship in D/H versus $^{18}O/^{16}O$ for the Cumberland subsamples, as presented in **Figure 5** of the main text and **Figure S10**, **Figure S11**, and **Figure S12**. No corrections based on these models were made to data presented in **Table S1**. Different physical and chemical processes manifest as different, known or predictable slopes in an isotope ratio-ratio plot. Calculation of these characteristic slopes requires input of the appropriate fractionation factors (i.e., alpha values) into physical and chemical models relevant to the dataset. We have normalized the Cumberland D/H and $^{18}O/^{16}O$ data with respect to the isotopic composition of water evolved during dehydroxylation of a hypothetical smectite ($\delta^{18}O_{VSMOW}$ = 31‰ and δD_{VSMOW} = 2024‰) because it is the relative change in the isotope ratios with respect to one another—rather than the absolute change—that presents as a process-diagnostic slope. The hypothetical smectite's oxygen isotopic composition corresponds to that expected for a smectite in isotopic equilibrium at 273 K with water having a $\delta^{18}O_{VSMOW}$ = 0‰. The smectite $\delta^{18}O_{VSMOW}$ value is also comparable to the median value for all Gale clay minerals (i.e., $\delta^{18}O_{VSMOW}$ ~ 38‰). The hypothetical smectite's hydrogen isotopic composition corresponds to the δD_{VSMOW} value reported by (142) for water evolved during a combustion experiment, which was designed in part to isolate high-temperature water associated with smectite dehydroxylation in a triple portion of Cumberland rock powder. **Figure S12** is presented as individual panels (**a**, **b**, **c**, and **d**) across four modeling subsections.

In **Figure S10**, we present the Cumberland data fit by York-type weighted linear least squares (WLLS) regressions (i.e., weighted to one standard error in both *x* and *y*) to the full dataset (CB1, CB2, CB3, CB5, CB6, CB7; black line), to the high-temperature smectite dehydroxylation dataset (CB3, CB5, CB6; blue dotted line), and to a dataset combining CB3, CB5, and CB6 with probable brucite dehydroxylation from CB7 (green dashed line). For simplicity, we assume that the errors in D/H and $^{18}O/^{16}O$ are uncorrelated. Regression equations, uncertainties (1 SE) on each fitted parameter, squares of the correlation coefficients, and p-values are as follows:

All Data: D/H = 4.9504 (± 0.4765) × $^{18}O/^{16}O$ – 3.5196 (± 0.4894)
r^2 = 0.7921; p-value = 0.0004848

Smectite Dehydroxylation (CB3, CB5, CB6):
D/H = 4.7268 (± 0.7897) × $^{18}O/^{16}O$ – 3.3222 (± 0.8165)
r^2 = 0.6459, p-value: 0.1054

Smectite + Brucite Dehydroxylation (CB3, CB5, CB6, CB7):
D/H = 4.2442 (± 0.6422) × $^{18}O/^{16}O$ – 2.8092 (± 0.6602)
r^2 = 0.6317, p-value: 0.02213

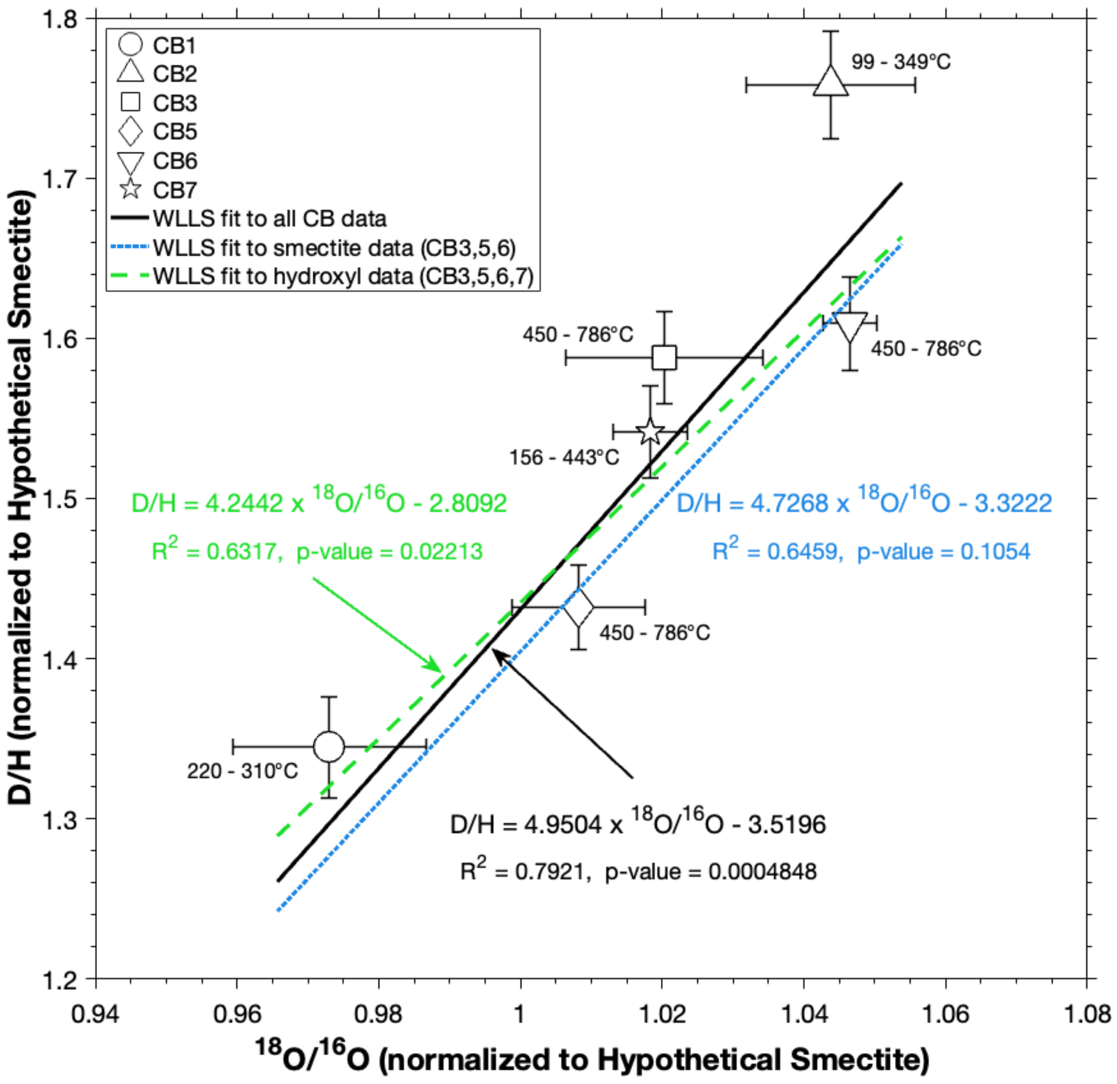

Figure S10. Comparison of trends in D/H versus $^{18}O/^{16}O$ space for different subsets of the Cumberland isotopic data (expanding upon Figure 5 in the main text). All data presented are normalized to the 'Hypothetical Smectite' (described in the Supplementary Text) for comparison. Temperature ranges correspond to the 'cut' of evolved water measured by TLS for each subsample. Error bars are two standard error of the mean. Regression lines are weighted (in both x and y) linear least squares fits (WLLS) to the normalized Cumberland data following (170). Solid black line is the best fit to all data. Dotted blue line is the best fit to the high-temperature data from smectite dehydroxylation (CB3, CB5, and CB6). Dashed green line is the best fit to smectite dehydroxylation from CB3, CB5, and CB6 and probable brucite dehydroxylation from CB7. Corresponding equations, R^2 values, and p-values for each regression are given in the same color as the line. Full equations with uncertainties on the regression parameters are as follows:

All data (black solid line): $D/H = 4.9504 (\pm 0.4765) \times {}^{18}O/^{16}O - 3.5196 (\pm 0.4894)$;

Smectite data (dashed blue line): $D/H = 4.7268 (\pm 0.7897) \times {}^{18}O/^{16}O - 3.3222 (\pm 0.8165)$;

Hydroxyl data (dashed green line): $D/H = 4.2442 (\pm 0.6422) \times {}^{18}O/^{16}O - 2.8092 (\pm 0.6602)$.

All three regression lines are indistinguishable from one another when uncertainties on the slopes and y-intercepts are included. As mentioned in the main text, this similarity could reflect a common paragenesis for the mineralogical sources of evolved water in each

of the six CB subsamples. To assess whether the linear relationship among the CB subsamples is random (null hypothesis), we performed a collinearity permutation test on the full Cumberland dataset. To do so, we randomly selected six paired $^{18}O/^{16}O$ and D/H ratios from the accepted TLS water isotopes dataset (i.e., those plotted in Figure 1 of the main text) and calculated their correlation coefficient (r) and its square (r^2). This process was performed 100,000 times, and the calculated r^2 values were each compared to the r^2 value calculated for the full Cumberland dataset to determine how many of the '6 random sample' simulations produced a collinearity equal to or greater than the Cumberland dataset. The resulting p-value of ~0.034 suggests that the Cumberland collinearity is unlikely to be random, leading to the conclusion that the mineralogical sources of evolved water are likely to be paragenetically related.

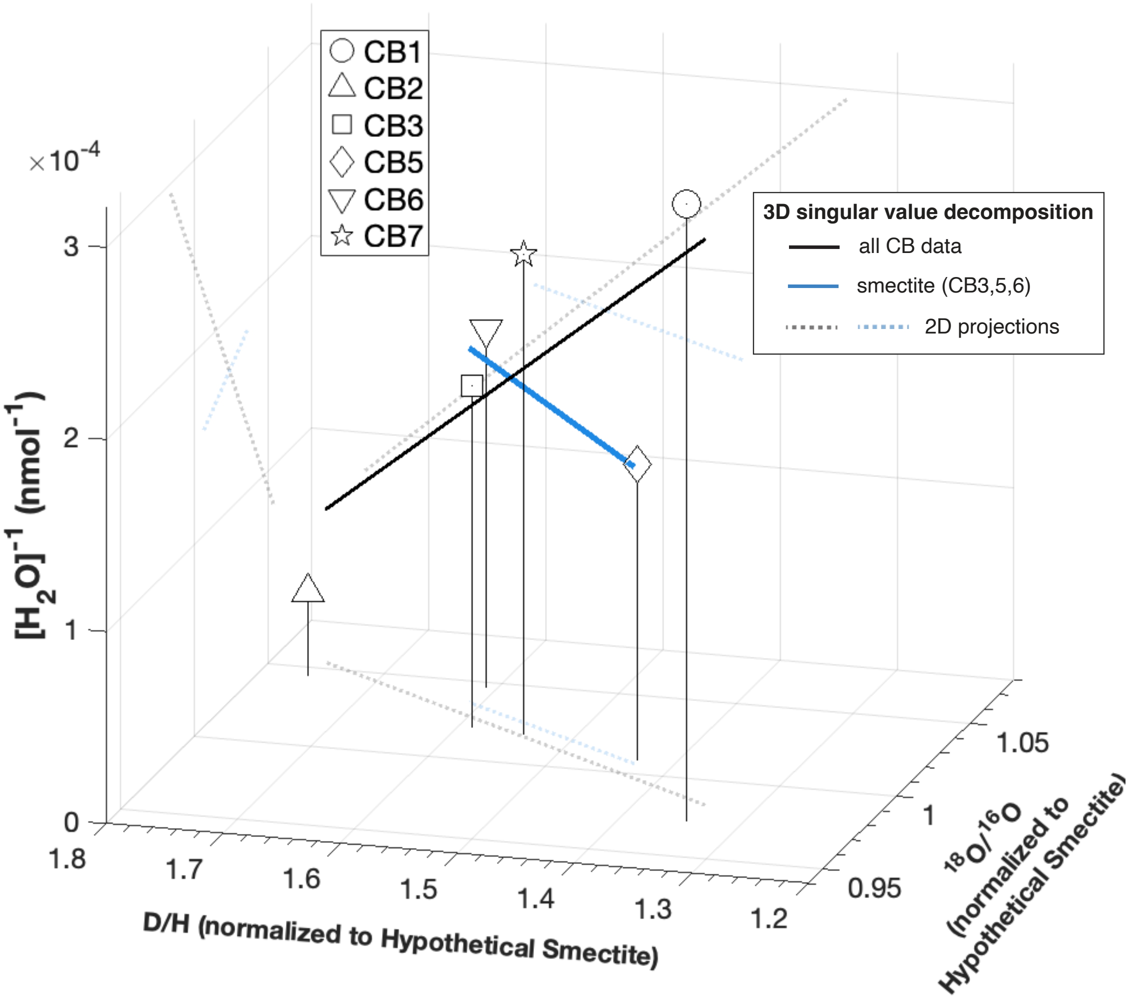

Figure S11. Evolved water abundances (in reciprocal nanomoles) are linearly correlated (positively sloped thick black line) with the D/H and $^{18}O/^{16}O$ ratios of the six Cumberland subsamples when the entire CB population is assessed together. In contrast, the smectite dehydroxylation subsamples (CB3, CB5, CB6) display a very slight negative correlation (thick blue line) between their evolved water abundances and their corresponding $^{18}O/^{16}O$ and D/H ratios. The black and blue orthogonal distance regression lines were calculated using Singular Value Decomposition. Error bars on isotope ratios are not given for figure

simplicity. All CB isotope ratios have been normalized to the corresponding D/H and $^{18}O/^{16}O$ values of the 'Hypothetical Smectite' described in the Supplementary Text. CB1, CB2, CB3, and CB5 were 'single portions' of rock powder whereas CB6 and CB7 were triple portions of the same. Due to the large absolute mass uncertainties for a given portion, we have pseudo-normalized the water concentrations of CB6 and CB7 by dividing them by three before taking the inverse. Faint dotted lines correspond to the projections of the three-dimensional regression lines into the corresponding two dimensions.

Diffusion-driven fractionation within the SAM instrument

Isotopic fractionation associated with gas-phase 'particle' diffusion through a gaseous medium of constant composition (e.g., the He carrier gas inside SAM) can be modeled via the equation for gas-phase interdiffusion:

$$\alpha_{\text{diffused-residual}} = \frac{1}{\sqrt{\left(\frac{M_i}{M_j}\right) \times \left(\frac{M_j + M_{\text{avg}}}{M_i + M_{\text{avg}}}\right)}} \qquad \textbf{Eqn. S3}$$

in which M_i = the molecular weight of the heavy isotopologue, M_j = the molecular weight of the light isotopologue, and M_{avg} = the mean molecular weight of the gas medium through which the other gas 'particles' are passing. The fractionation factor, alpha, describes the isotopic fractionation between the collection of molecules that has diffused (in the presence of an abundant background gas) from a hypothetical initial volume through an arbitrary restriction larger than the molecule's mean free path and the collection of molecules that remain (i.e., are residual) in the starting volume. The alpha value therefore equals the ratio $\frac{R^i_{\text{diffused}}}{R^i_{\text{residual}}}$ where $R^i = [i] / [j]$, the number of moles of the heavy isotopologue (i) over the number of moles of the light isotopologue (j). The hydrogen and oxygen isotope fractionation factors corresponding to gas-phase interdiffusion of water molecules in helium are thus $\alpha^D_{\text{diffused-residual}} = 0.99518$ for the $HD^{16}O/H_2{}^{16}O$ ratio and $\alpha^{18}_{\text{diffused-residual}} = 0.99085$ for the $H_2{}^{18}O/H_2{}^{16}O$ ratio. Both fractionation factors are less than 1, indicating that the material that has diffused out of the starting volume is depleted in the heavy isotopologue, as expected given **Equation S3**'s basis in the kinetic theory of gases. The slope in normalized D/H v. $^{18}O/^{16}O$ space for isotopic fractionation associated with interdiffusion of water vapor through the helium carrier gas was calculated using the expression $\left(1-\alpha^D_{\text{diffused-residual}}\right)/\left(1-\alpha^{18}_{\text{diffused-residual}}\right)$. The $1-\alpha$ formulation casts the slope with respect to the relative fractionation in D/H (~5‰) over that in $^{18}O/^{16}O$ (~10‰), leading to a slope of 0.5 in isotope ratio-ratio space (**Figure S12a**).

Figure S12a (next page). Assessment of isotopic fractionation processes that could lead to the linear relationship observed in Cumberland D/H versus $^{18}O/^{16}O$ data. All CB isotope ratios have been normalized to the corresponding D/H and $^{18}O/^{16}O$ values of the 'Hypothetical Smectite' described in the Supplementary Text. In all subplots, the weighted linear least squares fit (WLLS) to the Cumberland smectite data is given as a blue dotted line. Fit parameters have the same color as their corresponding regression line. Error bars correspond to two standard error of the mean. **(A)** Interdiffusion of water isotopologues ($H_2{}^{16}O$, $HD^{16}O$, and $H_2{}^{18}O$) through a He carrier gas as in SAM results in a slope of ~0.5 (red line) due to the relative differences in the isotopologue molecular masses.

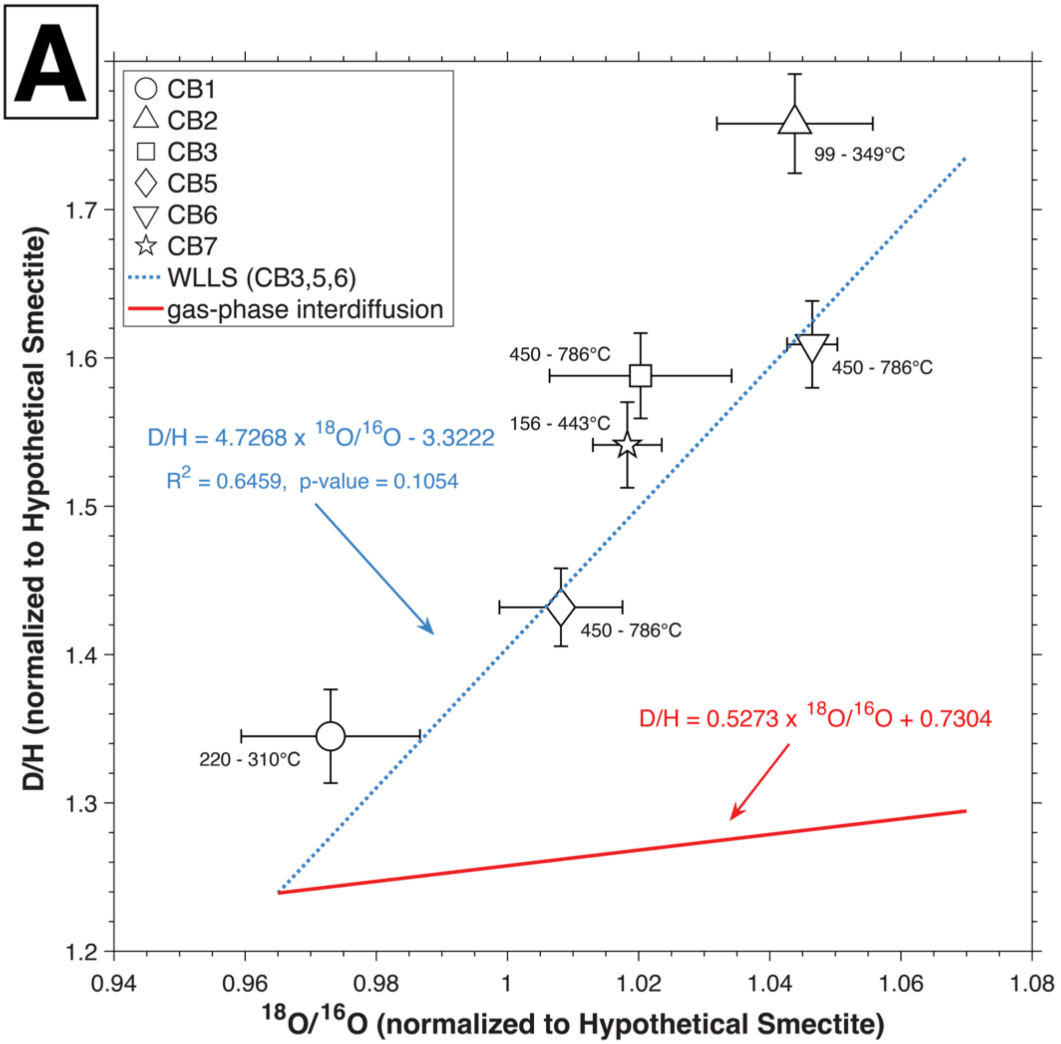

<u>Mineral-water equilibrium fractionation</u>

Equilibrium isotope fractionations are fundamentally exchange reactions—the transfer of isotopes between two phases or molecular species that share a common element. Equilibrium fractionation factors (α_{eqm}) describe this partitioning of isotopes between phases, between molecular species, and between phases and molecular species (e.g., mineral–water isotopic exchange) as a function of temperature (**Equations S4abc**; **Figure S12b**). We have compiled a list of mineral–water equilibrium fractionation factor expressions for hydrogen and oxygen isotope exchange based on existing literature data. These expressions (and the temperature-specific values used in the models) are given in **Table S5**. Minerals were chosen based on the *Curiosity* CheMin mineral assemblages and dominant mineralogical evolved water source for each sample in **Table S1**. In some cases, temperature-dependent expressions for a specific mineral and isotopic system were not available, necessitating the substitution of a mineralogically-related system instead.

Most significant among these 'substitutions' is the use of the published smectite–water oxygen isotopic fractionation factors, which were calculated based on fluorination

of natural smectite clays and thus integrate the oxygen isotopic compositions of tetrahedrally coordinated (i.e., Si-O-Si or Al-O-Si) and octahedrally coordinated hydroxyl (–OH) oxygen in the clay mineral lattice. The result is a weighted average of contributions from the two oxygen sites within the mineral. Based on studies of intracrystalline fractionation in kaolinite, the isotopic compositions of Si- or Al-bound oxygen and hydroxyl oxygen may differ by ~10–30‰ at temperatures of ~20°C (133). Comparable experimental work on illite indicates an ~15‰ fractionation between the tetrahedrally coordinated Si- or Al-bound oxygen and the octahedrally coordinated hydroxyl oxygen at 200°C, which decreases to ~8‰ at 300°C (171). In both experimental studies, the tetrahedrally coordinated oxygen is ^{18}O-enriched relative to the hydroxyl oxygen. As a result, our use of the 'integrated' smectite–water oxygen isotope fractionation factors compiled in **Table S5** and plotted in **Figure S12b** to calculate the isotopic composition of coexisting liquid water (and, hence, the resulting oxygen isotopic composition of smectite in equilibrium with that water) will always result in an underestimate of the true isotopic composition. We have chosen not to add a 'correction factor' to the Cumberland data based on the kaolinite intracrystalline fractionation results due to the significant mineralogical differences between smectite clay minerals and kaolinite clays. Similarly, we have chosen not to add a correction factor based on the illite experimental results due to the significant (~200°) temperature extrapolation that would be required for application to Gale crater samples.

Although several alpha values exist for montmorillonite (smectite)–water hydrogen isotope exchange, these values correspond to fractionation at a single temperature or inferred to correspond to a small temperature range; thus, we also include a temperature-dependent expression for mineral–water hydrogen isotope exchange in illite/smectite mixed clays. We present the illite–water oxygen isotope equilibrium fractionation factor for comparison to that of smectite–water even though illite has not been definitively identified in any clay samples from Gale crater (108). Likewise, goethite was chosen as a stand-in for akaganeite, and gypsum was chosen for bassanite although both bassanite–water and gypsum–water equilibrium fractionation factors have been determined from *ab initio* methods. We therefore also present the first principles results for comparison with the experimentally and naturally-determined gypsum–water results. Whenever possible, we have selected fractionation factors that have been determined—either experimentally, theoretically, or from a suite of equilibrated natural samples—over a temperature range suitable for liquid water-bearing systems on the surface of Mars.

In three cases, we recast the mineral–water equilibrium fractionation expressions given in the literature because those expressions were calculated based on fits to temperature (T) rather than to inverse temperature (T^{-1}) or to inverse temperature-squared (T^{-2}). The latter two forms are dependencies approximating the 'low-temperature' and 'high-temperature' limits of the statistical mechanics treatment of isotopic fractionation by (172) and thus are a more appropriate treatment of the relationship between alpha and temperature. The relevant formulations are:

$$1000 \ln \alpha_{\mathrm{eqm}} = C_1 + C_2/T \quad \textbf{Eqn. S4a},$$

$$1000 \ln \alpha_{\mathrm{eqm}} = C_1 + C_3/T^2 \quad \textbf{Eqn. S4b},$$

$$1000 \ln \alpha_{\mathrm{eqm}} = C_1 + C_2/T + C_3/T^2 \quad \textbf{Eqn. S4c}.$$

We recalculated the gypsum–water hydrogen and oxygen equilibrium fractionation expression given in (173) using the data provided in their Appendices; the reformulated expression is given in **Table S5**. There was no statistical difference between fitting the data with **Equation S4a** or **Equation S4b**; thus, we chose to use **Equation S4b**, as this formulation is more common in treatments of solid–water equilibria (e.g., (174)). We recast the third-order polynomial fit to the *ab initio* results for gypsum–water and bassanite–water fractionations presented in (175) using **Equation S4c**, as this functional form provided the best fit to the data. In **Figure S12b** (next page), we present the relevant mineral-water equilibrium isotope fractionation factors as a function of temperature. These alpha values correspond to the ratio $\frac{R^i_{\text{mineral}}}{R^i_{\text{water}}}$ where, as before, $R^i = [i] / [j]$, the number of moles of the heavy isotopologue (i) over the number of moles of the light isotopologue (j).

In our assessment of Cumberland data, we assumed for illustrative purposes a hypothetical starting isotopic composition for the water reservoir of D/H = 5 × VSMOW and $^{18}O/^{16}O$ = VSMOW based on existing data from martian materials. Equilibrium hydrogen and oxygen isotope fractionations between water structurally bound to specific minerals and the water reservoir itself were calculated for temperatures between 273 K and 373 K using the fractionation factors (or fractionation expressions) in **Table S5**. We acknowledge that this temperature range encompasses extrapolated portions of certain mineral-water fractionation expressions; however, this exercise was designed to illustrate the general slope of equilibrium fractionation trends compared to the slope in D/H versus $^{18}O/^{16}O$ space for the Cumberland data. Results are shown in **Figure S12b** below.

Figure S12b (next page). Assessment of isotopic fractionation processes that could lead to the linear relationship observed in Cumberland D/H versus $^{18}O/^{16}O$ data. All CB isotope ratios have been normalized to the corresponding D/H and $^{18}O/^{16}O$ values of the 'Hypothetical Smectite' described in the Supplementary Text. In all subplots, the weighted linear least squares fit (WLLS) to the Cumberland smectite data is given as a blue dotted line. Error bars correspond to two standard error of the mean. **(B)** Equilibrium isotope fractionation between various minerals and water of a constant (hypothetical) isotopic composition (black star) leads to negative or flat slopes in D/H versus $^{18}O/^{16}O$ space. Equilibrium fractionations were calculated over a temperature range of 373 K (left-most) to 273 K (right-most) for illustrative purposes. The reader is referred to **Table S5** and **Figure S13** for information on the different fractionation factors used.

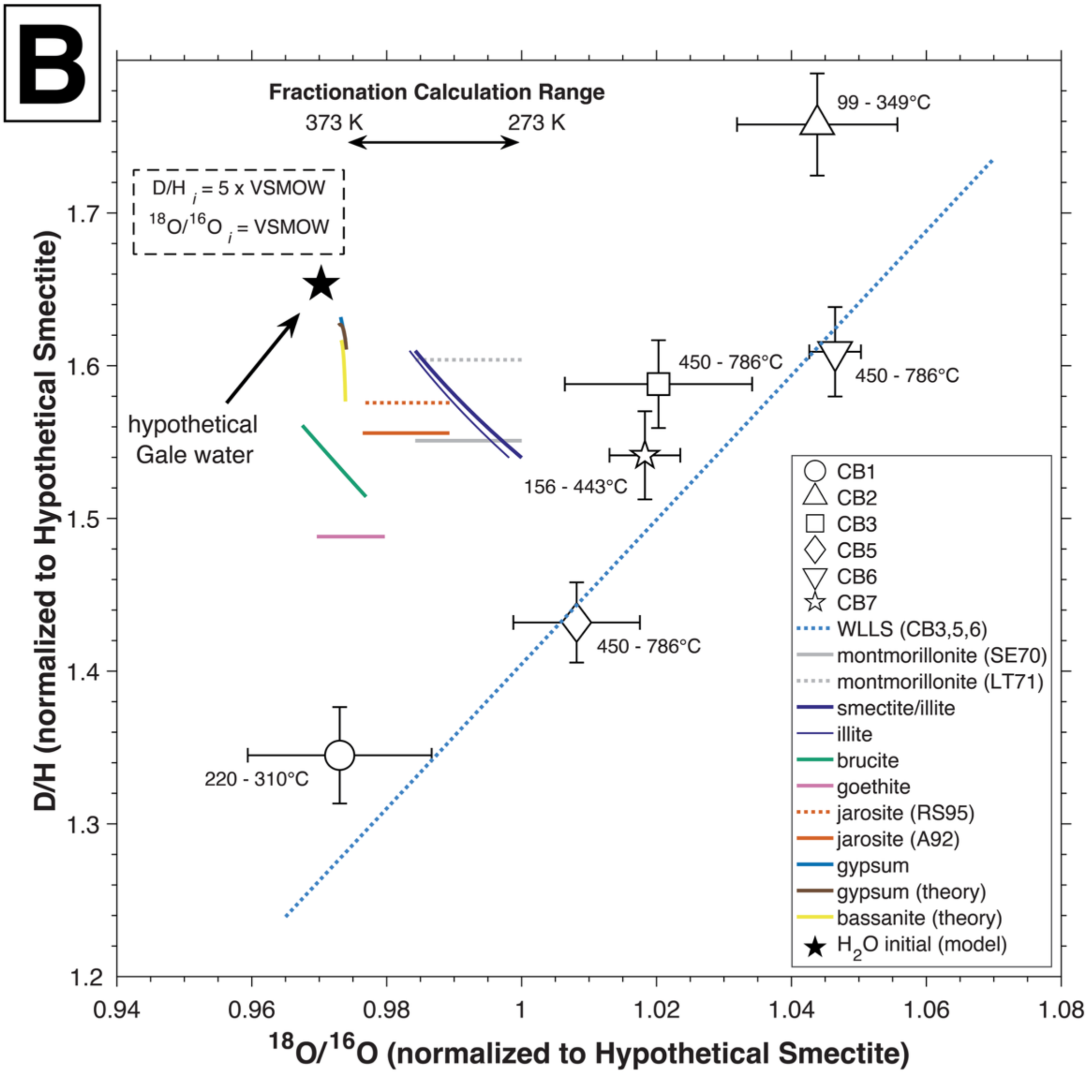

<u>Rayleigh fractionation via evaporation into an infinite atmosphere under conditions of liquid-vapor thermodynamic equilibrium</u>

Rayleigh fractionation mathematically describes the isotopic evolution of components within an open system as a function of the progressive removal—in fractional increments—of a minor component from a larger, well-mixed reservoir. Each increment is assumed to form in isotopic equilibrium with the reservoir but is then completely removed from the system (i.e., no additional isotopic exchange occurs between the reservoir and the vapor increment). Rayleigh processes are formulated via the following differential equation:

$$d \ln R = (\alpha - 1) d \ln f \qquad \textbf{Eqn. S5}.$$

In this expression, R represents the isotopic ratio of interest in the reservoir, α is the fractionation factor describing how the heavy and light isotopes are partitioned between

the reservoir and the removed increment, and f is a progress variable indicating the fraction of original material remaining in the reservoir. In **Equation S5**, alpha represents the equilibrium liquid-vapor isotopic fractionation factor, α_{eqm}. Note that the traditional formulation of α_{eqm} in vapor pressure isotope effect calculations is written as $\alpha_{vapor-liquid}$, whereas the Majoube formulation (**Table S6)** calculates the inverse, $\alpha_{liquid-vapor}$. We therefore use the reciprocal of the Majoube alpha value when solving **Equation S5**.

In this general Rayleigh model for evaporation, we assumed that the water reservoir's starting isotopic composition was equivalent to that which would be in isotopic equilibrium with the hypothetical smectite at 273 K and 323 K. The two temperatures were selected to simulate near-freezing and warm waters, respectively, as end-member cases for illustration. To determine the instantaneous isotopic composition of the evaporating reservoir, we numerically integrated and iteratively solved **Equation S5** using a step-size of 0.001 (f, constant amount of liquid removed from the liquid reservoir) and assuming a constant fractionation factor between liquid water and water vapor for the hydrogen ($\alpha^{\mathrm{D}}_{\text{liquid-vapor}}$) and oxygen ($\alpha^{18}_{\text{liquid-vapor}}$) isotope systems. The corresponding liquid-vapor alpha values are $\alpha^{\mathrm{D}}_{\text{liquid-vapor}} = 1.113$ and $\alpha^{18}_{\text{liquid-vapor}} = 1.012$ at 273 K and $\alpha^{\mathrm{D}}_{\text{liquid-vapor}} = 1.056$ and $\alpha^{18}_{\text{liquid-vapor}} = 1.008$ at 323 K (calculated from equations in **Table S6**). Although dissolved solutes would suppress isotopic fractionation associated with the vapor pressure isotope effect (176), the magnitude of these salt effects at the $MgCl_2$ concentrations (~0.1 M) required to saturate the Cumberland smectites with Mg is small (<1 to 2‰) compared to the TLS measurement uncertainties (177). In the absence of additional geochemical constraints on the early diagenetic fluid compositions at Cumberland, such effects are not modeled here. The results from calculations at 273 K are plotted in **Figure S14**; comparable calculations for the same system at 323 K result in a shallower slope of ~7.7 and require complete evaporation to capture the spread in Cumberland data. Chemical—i.e., total moles of H_2O—and isotopic mass balance were maintained throughout the calculation. Material flux in this system occurs in one direction only: liquid to vapor via evaporation.

We then used the D/H and $^{18}O/^{16}O$ values of the residual water reservoir at each 'timestep' to determine the corresponding instantaneous D/H and $^{18}O/^{16}O$ composition of mineralogical water—in this case, hydroxyl groups incorporated during authigenic clay formation—in equilibrium with that reservoir. The instantaneous $^{18}O/^{16}O$ of the clay hydroxyl water at 273 K and at 323 K was calculated using the smectite mineral–water equilibrium expression given in **Table S5** without any correction for isotopic fractionation between the octahedrally coordinated hydroxyl oxygens and tetrahedrally coordinated Si- or Al-bound oxygens in the smectite crystal lattice. The instantaneous D/H of the clay hydroxyl water was calculated at 273 K using the (178) montmorillonite alpha value (0.938), while that at 323 K was calculated using the (179) montmorillonite alpha value (0.970), thus avoiding potential pitfalls associated with extrapolation of the expression by (180) to much lower temperatures. Even so, use of the Lawrence & Taylor (179) value assumes its validity at temperatures higher than those of modern terrestrial surface weathering conditions (~5 to 30°C) and thus likely represents an overestimate of the actual hydrogen isotope fractionation between clay and water to be expected at 50°C. The clay hydroxyl D/H and $^{18}O/^{16}O$ isotopic compositions calculated in this way were then divided by the absolute D/H and $^{18}O/^{16}O$ isotopic ratios of the hypothetical smectite to normalize

them in the same fashion as all the Cumberland isotope ratios. The model results for this simple Rayleigh scenario are presented in **Figure S12c** below.

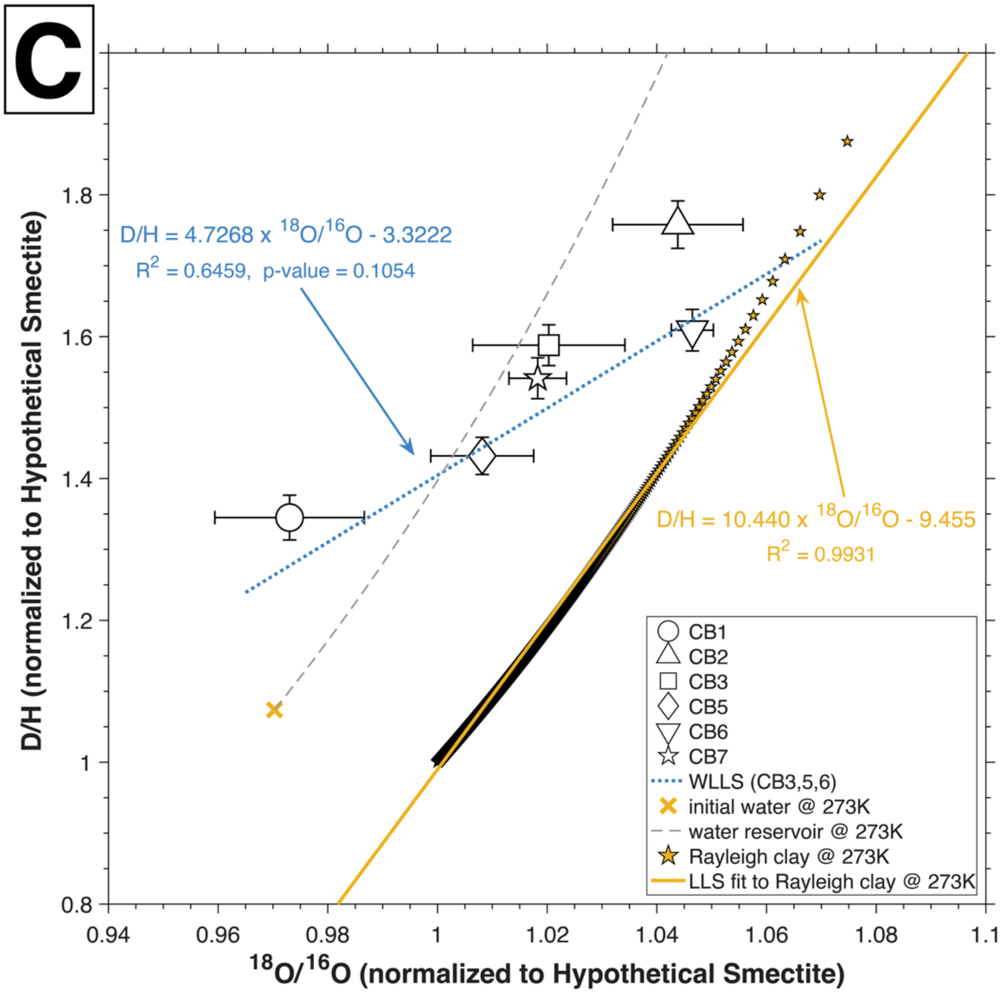

Figure S12c. Assessment of isotopic fractionation processes that could lead to the linear relationship observed in Cumberland D/H versus $^{18}O/^{16}O$ data. All CB isotope ratios have been normalized to the corresponding D/H and $^{18}O/^{16}O$ values of the 'Hypothetical Smectite' described in the Supplementary Text. In all subplots, the weighted linear least squares fit (WLLS) to the Cumberland smectite data is given as a blue dotted line. Fit parameters have the same color as their corresponding regression line. Error bars correspond to two standard error of the mean. **(C)** The isotopic composition of authigenic smectite passively recording the evolving isotopic composition of a water body undergoing Rayleigh distillative loss at 273 K (yellow stars). Increments—represented as individual symbols—are calculated based on the fraction of initial water remaining in the reservoir, with the last increment corresponding to $f = 0.01$ (i.e., 1%). Starting water composition (bolded yellow X) is assumed to be that which is in equilibrium with the 'Hypothetical Smectite'. The isotopic composition of the residual water in the reservoir is presented as a dashed gray line.

Craig-Gordon model for evaporative fractionation into a low-humidity atmosphere of constant isotopic composition

The Craig-Gordon model uses a modified Rayleigh expression that accounts for fluxes in two directions—out of and back into the liquid reservoir—by incorporating an empirical relative humidity term that serves as a physical metric accounting for the 'reversibility' of the evaporative flux, i.e., the ability of water molecules to leave the liquid without recondensation. The relevant Rayleigh differential equation in this case is like that in **Equation S5**:

$$d\ln R = \left({}^{1}\!/\alpha_{evap} - 1\right) d\ln f \qquad \textbf{Eqn. S6}$$

The term α_{evap} in **Equation S6** is the isotopic fractionation factor between water and water vapor at a relative humidity $h < 1$. Relative humidity is defined as the ratio of the actual partial pressure of water vapor (p_{H2O}) to the saturation (equilibrium) vapor pressure at a given temperature ($p_{satH2O,T}$). In the Craig-Gordon model, the relevant temperature is that at the water surface, and water vapor is treated as an ideal gas (181). Note that, unlike absolute humidity, which is a function of both p_{H2O} and total atmospheric pressure, the relative humidity depends only on the partial pressure of water with respect to its saturation (equilibrium) vapor pressure. The latter is a characteristic property of water that varies as a function of temperature. Because neither term depends upon the total atmospheric pressure, we need not account for differences between martian (e.g., ~500 mbar) and terrestrial (e.g., 1 bar) atmospheric pressures in our calculations.

In this formulation, α_{evap} is calculated as $\alpha_{liquid-vapor}$, which can be directly compared to the equilibrium alpha values calculated using the equations in **Table S6**. The α_{evap} fractionation factor, which changes continuously over the course of an evaporative process, is parameterized as a function of the relative humidity (h), the same equilibrium isotope fractionation factor (α_{eqm}) between liquid and vapor implemented in **Equation S5**, a disequilibrium fractionation factor (α_{evap}^{0}) corresponding to the instantaneous isotopic fractionation between the water reservoir and a completely dry ($h = 0$) atmosphere, and two isotope ratios—that of the water reservoir (R_w) and the atmospheric water vapor (R_v):

$$\alpha_{evap} = \frac{\alpha_{evap}^{0}(1-h)}{1-\alpha_{eqm} h R_v / R_w} \qquad \textbf{Eqn. S7.}$$

The ratio R_v reflects the isotopic composition of an air mass assumed to be substantially larger than the water reservoir (i.e., effectively infinite) and thus can be treated as a constant. We modeled the evaporative process using a step-size of 0.001 (f, constant amount of liquid removed from the liquid reservoir), as in the simple Rayleigh case, and the constants given in **Table S7** for the initial $^{18}O/^{16}O$ and D/H values of the liquid reservoir, R_w, before any evaporation has occurred. As in the Rayleigh distillation scenario, the initial water reservoir's isotopic composition was assumed to be that in equilibrium with the hypothetical smectite's hydroxyl water at 273 K. The zero-humidity disequilibrium fractionation factor (α_{evap}^{0}) was calculated at 273 K using the relationship $\alpha_{evap}^{0} = \alpha_{eqm} \times \sqrt{D/D^{*}}$. In this expression, D/D^{*} corresponds to the ratio of isotopologue diffusivities, here calculated using the expression in the denominator of **Equation S3**, the

molar mass of CO_2 for M_{avg}, and the equilibrium liquid-vapor fractionation factors given in **Table S6** without modification.

To model a given evaporation 'event' at each timestep, we first calculated α_{evap} using **Equation S7** and the pre-evaporation value of R_w. We then used the numerically integrated version of **Equation S6** with the calculated α_{evap} value to determine the post-evaporation isotopic composition of the liquid reservoir, R_w. This post-evaporation R_w then became the pre-evaporation value for the next timestep, and the same process repeated iteratively.

We used the D/H and $^{18}O/^{16}O$ values of the post-evaporation water at each timestep to determine the corresponding instantaneous D/H and $^{18}O/^{16}O$ composition of mineralogical water—as before, hydroxyl groups incorporated during authigenic clay formation—in equilibrium with the water reservoir. The instantaneous $^{18}O/^{16}O$ of the clay hydroxyl water at 273 K was calculated using the smectite-water equilibrium expression given in **Table S5** without any correction for isotopic fractionation between hydroxyl oxygens and tetrahedrally coordinated oxygens in the smectite crystal lattice. The instantaneous D/H of the clay hydroxyl water was calculated using the (178) montmorillonite–water equilibrium alpha value (0.938; **Table S5**). The resulting clay hydroxyl water isotopic compositions at each timestep were then normalized to the hypothetical smectite isotope ratios. Relative humidities were chosen based on measurements by the REMS instrument on *Curiosity*, which show strong diurnal fluctuations, with daytime values (between ~10:00–18:00 LMST) typically <5%, while seasonal fluctuations lead to daily maxima (between 04:00–06:00 LMST) of ~10% in late spring/early summer and up to ~70% in early winter at Gale (182). The Craig-Gordon fractionation model results are plotted in **Figure S12d** on the following page.

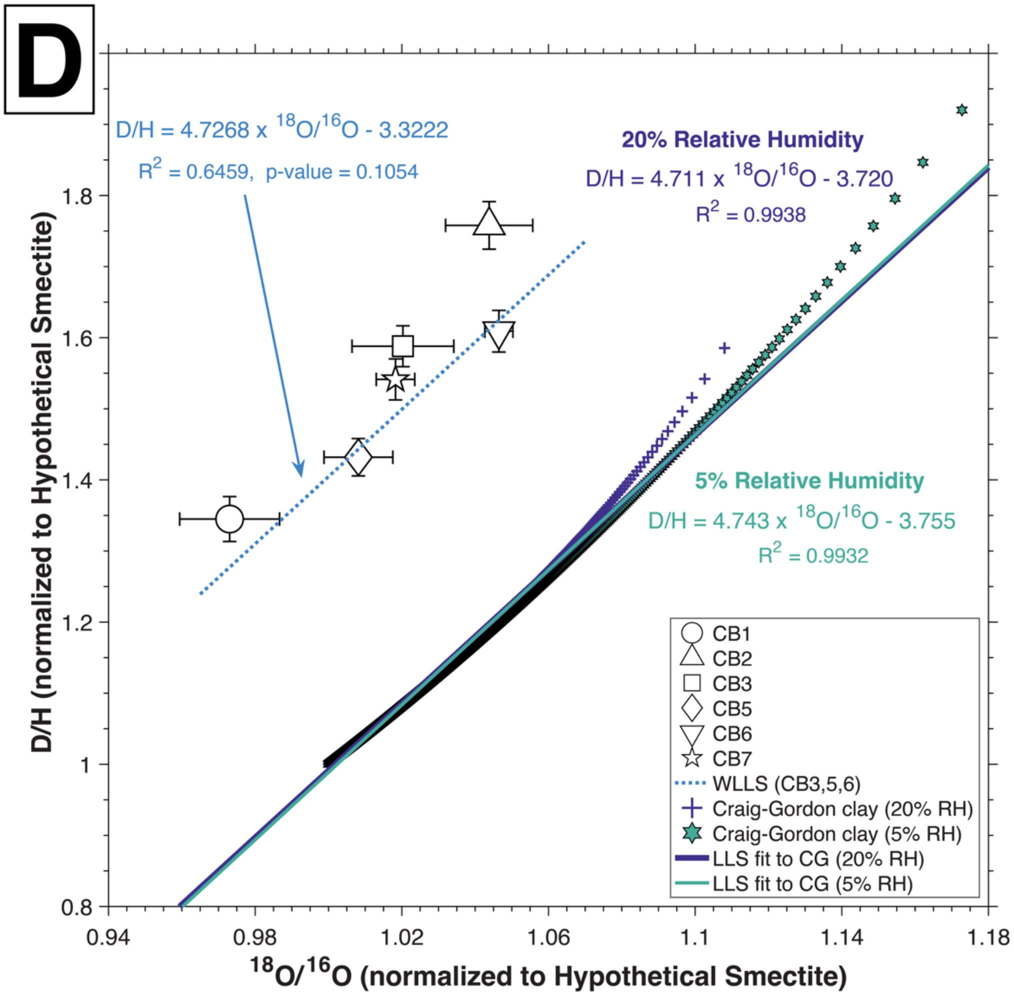

Figure S12d. Assessment of isotopic fractionation processes that could lead to the linear relationship observed in Cumberland D/H versus $^{18}O/^{16}O$ data. All CB isotope ratios have been normalized to the corresponding D/H and $^{18}O/^{16}O$ values of the 'Hypothetical Smectite' described in the Supplementary Text. In all subplots, the weighted linear least squares fit (WLLS) to the Cumberland smectite data is given as a blue dotted line. Fit parameters have the same color as their corresponding regression line. Error bars correspond to two standard error of the mean. **(D)** The isotopic composition of authigenic smectite passively recording the evolving isotopic composition of a water body undergoing evaporation at 273 K into ambient air with a relative humidity of either 20% (purple crosses) or 5% (turquoise hexagons). Increments—represented as individual symbols—are calculated based on the fraction of initial water remaining in the reservoir, with the last increment corresponding to $f = 0.01$ (i.e., 1%). Slopes for this 'Craig-Gordon' model of evaporative fractionation at different relative humidities are indistinguishable from one another and the weighted linear least squares fit (WLLS) to the Cumberland smectite data (blue dotted line).

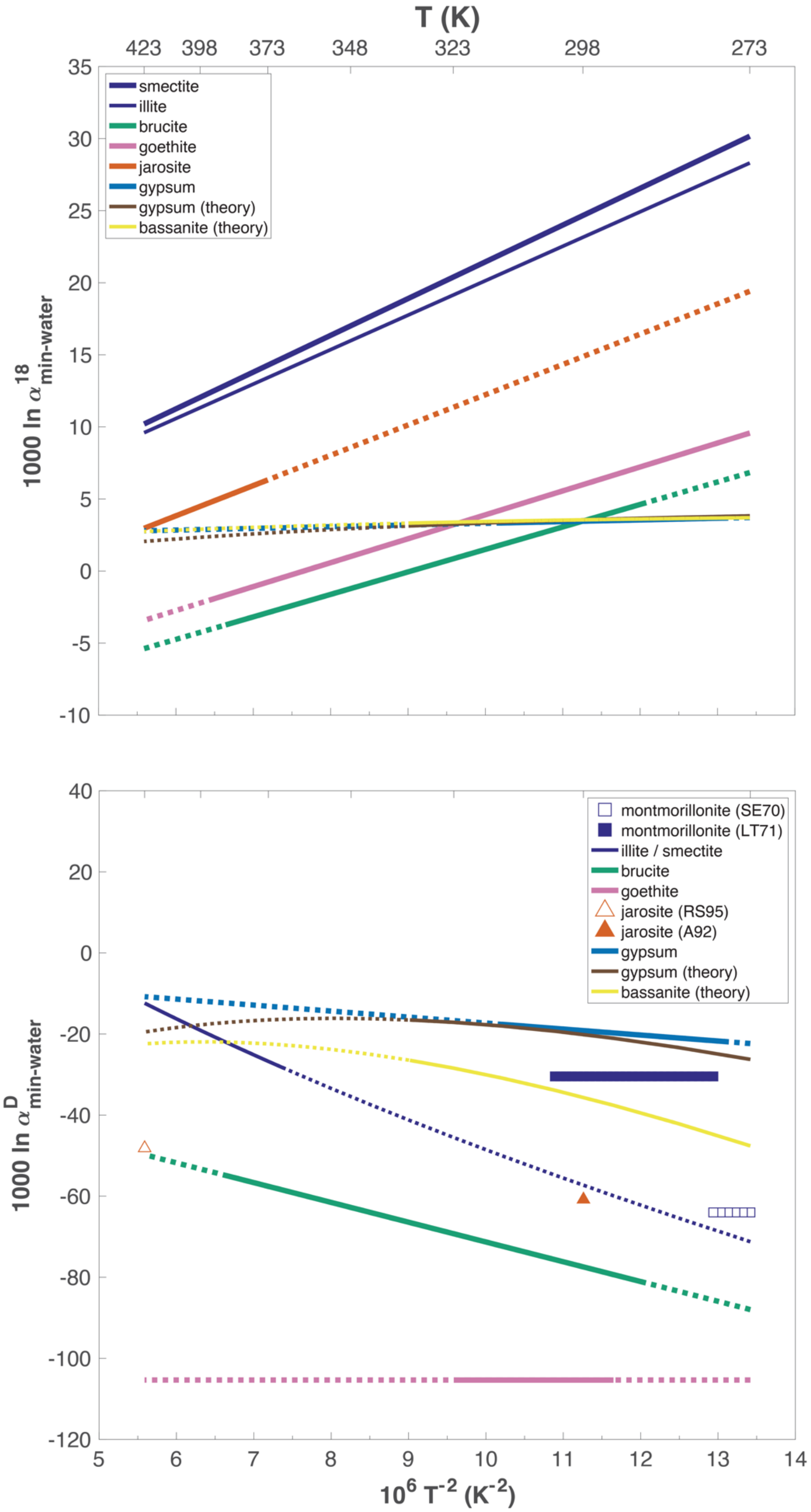
T (K)
423
398
373
348
323
298
273
smectite
illite
brucite
goethite
jarosite
gypsum
gypsum (theory)
bassanite (theory)
1000 ln α18min-water
35
30
25
20
15
10
5
0
-5
-10
montmorillonite (SE70)
montmorillonite (LT71)
illite / smectite
brucite
goethite
jarosite (RS95)
jarosite (A92)
gypsum
gypsum (theory)
bassanite (theory)
1000 ln αDmin-water
40
20
0
-20
-40
-60
-80
-100
-120
5
6
7
8
9
10
11
12
13
14
10^6 T^-2 (K^-2)

Figure S13 (previous page). Equilibrium isotopic fractionation factors for hydrogen ($\boldsymbol{\alpha}^{\mathrm{D}}_{\text{mineral-H2O}}$) and oxygen ($\boldsymbol{\alpha}^{18}_{\text{mineral-H2O}}$) isotope exchange between various minerals and liquid water as a function of temperature. Fractionation factors were calculated using the expressions given in **Table S5**. Solid lines represent the temperature range over which the equation was determined. Dashed lines represent the extrapolation of an individual equation to temperatures outside of the experimentally (or theoretically) determined range. Note that it is not strictly correct to use extrapolated fractionation factors; rather, they are presented for visualization purposes. Individual data points correspond to those studies in **Table S5** for which an equation was not determined. SE70 = Savin & Epstein (1970); LT71 = Lawrence & Taylor (1971); RS95 = Rye & Stoffregen (1995); A92 = Alpers (1992).

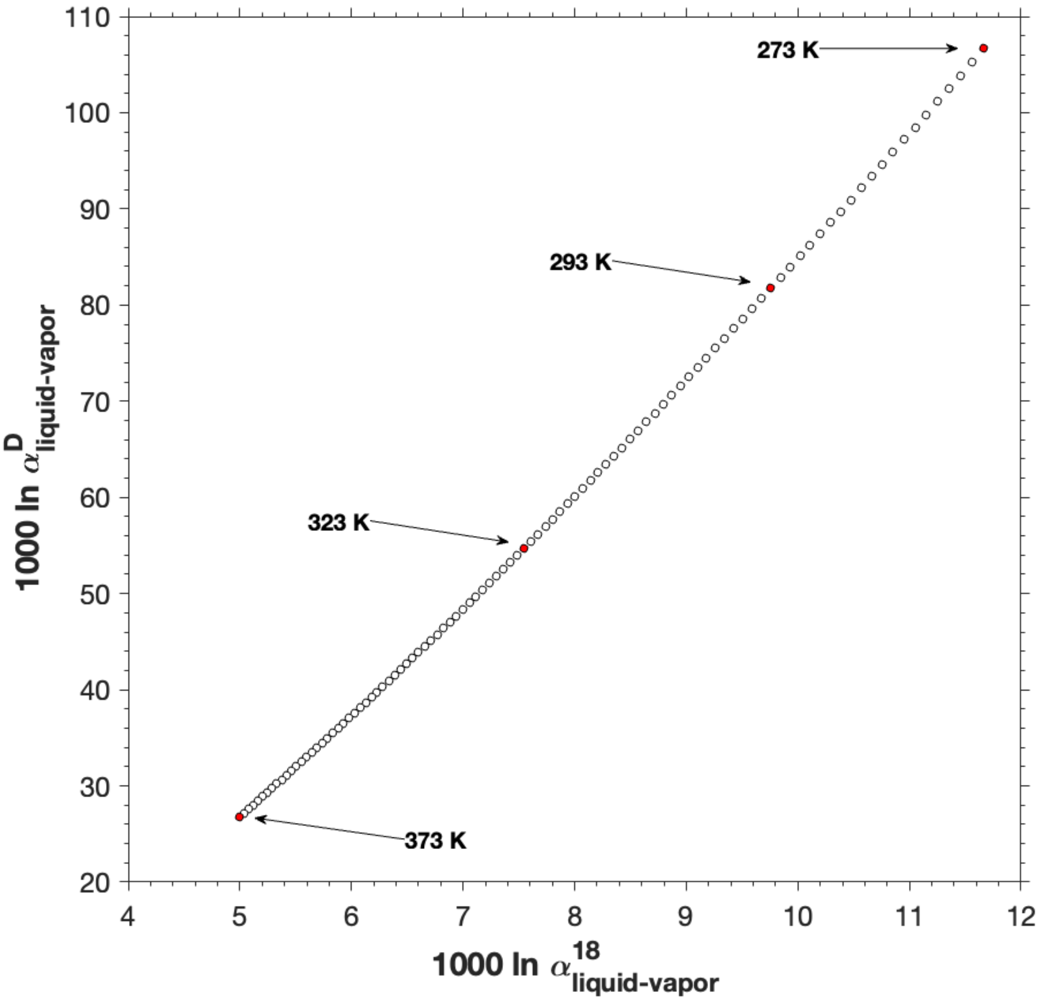

Figure S14. Vapor pressure isotope effect (VPIE) equilibrium fractionation factors for hydrogen ($\boldsymbol{\alpha}^{\mathrm{D}}_{\text{liquid-vapor}}$) and oxygen ($\boldsymbol{\alpha}^{18}_{\text{liquid-vapor}}$) isotope exchange between liquid water and water vapor calculated over the range 273–373 K via the equations from (147) given in **Table S6**. Filled circles highlight individual alpha pairs for the labeled temperature. Note that the absolute magnitude of isotopic fractionation in the two systems differ, but in both cases the liquid phase is preferentially enriched in the heavy isotope (D, ^{18}O).

SI Tables

Table S1. SAM-TLS water isotope values from evolved gas analyses of individual powdered (or scooped) rock samples. Sample identities correspond to named sites described in **Table S2**. As described in the Methods section of the main text, TLS ingests water vapor evolved over a predetermined temperature range, which, in conjunction with CheMin data, can be used to infer the likely mineralogical source of the ingested water. Multiple analyses with the same rock ID (e.g., JK2, JK3) are separate sieved aliquots from the same drilled (or scooped) powder. All paired isotope data collected prior to the clay-sulfate transition region are reported. See text above for an explanation of which data points have been culled from Figure 1 in the main text and not included in the discussion of results. Mineralogy and lithology designations are explained below the table. Numerical values for the VSMOW hydrogen and oxygen isotopic compositions used in this manuscript are provided below the table and based on data in Meija et al. (2016) (113). Numbers in the TID (test identification) column can be used to find the corresponding SAM-TLS Level 1a (spectral) datasets on NASA's Planetary Data System (PDS) Geosciences node: https://pds-geosciences.wustl.edu/msl/msl-m-sam-2-rdr-l0-v1/mslsam_1xxx/data/.

ID	TID	Analysis Sol	T Range (°C)	H_2O (nmol)	± 2 SE (nmol)	D/H (x E-4)	± 2 SE (x E-6)	δD_{VSMOW} (‰)	± 2 SE (‰)	$^{18}O/^{16}O$ (x E-3)	± 2 SE (x E-5)	$\delta^{18}O_{VSMOW}$ (‰)	± 2 SE (‰)	Mineralogy †	Lithology ‡
RN2	25041	96	440–601	3818	24	6.6028	6.5265	3239	42	2.5482	4.1146	271	21	A	H
RN3	25044	99	234–425	15845	88	7.9206	6.0810	4085	39	2.1403	2.1423	67	11	B	H
RN4	25048	117	349–450	7265	46	6.8583	6.7103	3403	43	1.9943	1.5632	-5	8	B	H
JK2*	25067	199	650–801	2907	20	6.1215	9.6231	2930	62	2.0706	2.9324	33	15	C	I
JK3*	25074	224	750–801	5179	55	6.9097	7.2275	3436	46	2.0699	2.2310	32	11	C	I
JK4	25077	227	300–549	5976	20	6.9502	6.0343	3462	39	2.0494	1.5015	22	7	A, B	I
CB1	25087	281	220–319	3114	14	6.3349	10.118	3067	65	2.0108	2.8161	3	14	B	I
CB2	25091	286	99–349	22654	178	8.2804	6.7259	4316	43	2.1571	2.4563	76	12	B	I
CB3	25094	290	450–786	5620	26	7.4798	4.2492	3802	27	2.1085	2.8686	52	14	C, A	I
CB5	25123	368	450–786	6464	28	6.7446	4.2960	3330	28	2.0834	1.9390	39	10	C, A	I
CB6*	25130	382	450–786	16080	50	7.5795	4.5234	3866	29	2.1626	0.7912	79	4	C	I
CB7*	25142	415	159–499	11987	54	7.2602	5.3770	3661	35	2.1043	1.0848	49	5	B, C2	I
CH	25226	773	39–156	3259	10	5.5016	10.692	2532	69	2.0650	2.6027	30	13	B	I
MJ	25242	887	39–156	4778	12	5.6714	5.4517	2641	35	1.9804	3.0904	-12	15	B	I
TP	25257	928	301–577	8830	22	6.6916	3.4985	3296	22	2.0337	1.3988	14	7	D	I
BS	25297	1130	607–897	36648	168	7.4455	4.9907	3780	32	2.1339	2.2446	64	11	E	J
GH1	25306	1147	608–898	37640	224	7.3085	4.2337	3692	27	2.0268	1.5745	11	8	E	J
GB1	25327	1224	34–147	984	2	5.3131	33.957	2411	218	1.8596	9.0032	-73	45	B	H
GB2	25333	1237	534–798	2978	6	6.6184	9.3427	3249	60	2.1016	2.8465	48	14	F	H
OU	25350	1382	264–797	8477	20	6.6106	5.6387	3244	36	2.0815	2.0140	38	10	G**, B2	J, K
MB	25366	1443	268–799	10447	74	6.2025	5.7508	2982	37	2.0752	2.2129	35	11	C	I
QL	25413	1722	432–895	34694	470	5.0203	4.7570	2223	31	2.0592	2.2462	27	11	C	I

DU	25484	2072	500–873	7052	32	6.0779	4.1464	2902	27	2.1072	2.3950	51	12	C	I
ST	25493	2147	341–788	5424	28	6.4362	8.0655	3132	52	2.0512	2.8461	23	14	D2	I
HF	25500	2231	234–451	2508	10	6.5561	10.418	3209	67	2.0140	3.6927	4	18	B2**, G	I
RH	25505	2281	285–472	3821	24	6.7010	10.368	3302	67	2.0055	3.6282	0	18	B**, D2	I
KM1	25515	2393	374–549	6803	28	7.3318	7.7446	3707	50	2.0739	1.7301	34	9	C	I
GE1	25530	2497	268–541	32332	220	7.6371	6.5359	3903	42	2.0801	1.9763	37	10	C	K
GE3	25538	2531	346–600	14168	54	7.6418	3.1527	3906	20	2.1034	1.6835	49	8	C	K
HU1	25565	2677	452–765	2007	4	7.0655	12.421	3536	80	2.0605	3.5247	28	18	G	I
EB	25572	2721	264–538	13297	54	7.8443	4.7072	4036	30	2.0426	1.2179	19	6	G**, E	J
GG	25579	2765	270–556	26071	134	7.4658	6.0717	3793	39	2.0326	0.9994	14	5	C**, B2	I
MA1	25596	2844	270–556	47748	368	7.9253	7.5608	4088	49	2.0398	1.6924	17	8	C**, B2	K
NT	25635	3061	299–549	27888	70	7.7508	3.6947	3976	24	2.0857	1.0086	40	5	C**, B2	I

VSMOW D/H = 1.5576426 $\times 10^{-4}$; $^{18}O/^{16}O = 2.005171 \times 10^{-3}$ based on relative abundances of the isotopes given in (113).
*These samples underwent an additional preparatory step in which they were heated (25 min at 320°C for JK2, JK3; 27 min at 250°C for CB6, CB7) prior to the EGA and GCMS analysis to release and vent low-temperature volatiles, including MTBSTFA by-products (139).
**These mineralogy designations represent the dominant sources of evolved water during these sample runs at the temperatures of the TLS cut.
†Column labeled "Mineralogy" indicates the mineralogical sources of water contributing to the integrated water aliquot analyzed by the TLS.
‡Column labeled "Lithology" indicates the rock type from which the sample was collected.

†Mineralogy Designations
A: residual adsorbed water, residual hydrated salt water
B: adsorbed water to ~200°C, <450°C hydrated salt water (e.g., perchlorate/chlorate; Mg,Ca-chlorides; Ca,Mg,Fe sulfates; Fe-oxyhydroxides)
B2: adsorbed water to ~200°C, <450°C hydrated salt water (e.g., Mg,Ca-chlorides; Ca,Mg,Fe-sulfates; Fe-oxyhydroxides; perchlorate NOT detected (183-185))
C: smectite
C2: intercalated Mg-hydroxyl ('brucite') groups within partially chloritized smectite (177)
D: jarosite with possible contributions from A, B
D2: jarosite and smectite / phyllosilicate
E: dissolved water from poorly crystalline phases (186, 187)
F: mixture of A and E
G: phyllosilicate (likely smectite but XRD peak too low to assign phyllosilicate phase (188))

‡Lithology Designations
H: modern aeolian
I: lacustrine mudstone
J: aeolian sandstone
K: near-shore/fluvial siltstone/sandstone

Table S2. Sample identification information for rock powders analyzed by SAM-TLS. Samples were kept in the sample processing unit onboard *Curiosity* (CHIMRA) between physical collection sol and SAM analysis. Multiple collection sols correspond to different numbered subsamples given in **Table S1** (e.g., RN1, RN2).

Sample	Abbreviation	Sol(s) Collected	Unit // Formation (Rock Type)	Depositional Environment
Rocknest	RN	61, 66, 69, 74, 93	Wind-blown dust / sand mixtures from multiple lithologies	modern aeolian
John Klein	JK	182	Bradbury // Yellowknife Bay (laminated mudstone with minor sandstone)	lacustrine
Cumberland	CB	279	Bradbury // Yellowknife Bay (laminated mudstone with minor sandstone)	lacustrine
Confidence Hills	CH	759	Mount Sharp // Murray (laminated mudstone with minor sandstone)	lacustrine
Mojave2	MJ	882	Mount Sharp // Murray (laminated mudstone with minor sandstone)	lacustrine
Telegraph Peak	TP	908	Mount Sharp // Murray (laminated mudstone with minor sandstone)	lacustrine
Big Sky	BS	1119	Siccar Point // Stimson (sandstone)	ancient aeolian
Greenhorn	GH	1137	Siccar Point // Stimson (sandstone)	ancient aeolian
Gobabeb	GB	1224	Wind-blown dust / sand mixtures from multiple lithologies	modern aeolian
Oudam	OU	1361	Mount Sharp // Murray (cross-stratified sandstone)	reworked aeolian and fluvial
Marimba	MB	1422	Mount Sharp // Murray (laminated mudstone with minor sandstone)	lacustrine
Quela	QL	1464	Mount Sharp // Murray (laminated mudstone with minor sandstone)	lacustrine
Duluth	DU	2057	Mount Sharp // Murray (laminated mudstone with minor sandstone)	lacustrine
Stoer	ST	2136	Mount Sharp // Murray (laminated mudstone with minor sandstone)	lacustrine
Highfield	HF	2223	Mount Sharp // Murray (laminated mudstone with minor sandstone)	lacustrine
Rock Hall	RH	2261	Mount Sharp // Murray (laminated mudstone with minor sandstone)	lacustrine
Kilmarie	KM	2384	Mount Sharp // Murray (laminated mudstone)	lacustrine
Glen Etive	GE	2486, 2527	Mount Sharp // Carolyn Shoemaker (cross-stratified pebbly sandstone)	fluvial
Hutton	HU	2668	Mount Sharp // Carolyn Shoemaker (faintly laminated mudstone)	lacustrine
Edinburgh	EB	2711	Siccar Point // Stimson (sandstone)	ancient aeolian
Glasgow	GG	2754	Mount Sharp // Carolyn Shoemaker (faintly laminated mudstone)	lacustrine
Mary Anning	MA	2838, 2870	Mount Sharp // Carolyn Shoemaker (cross-stratified pebbly sandstone)	fluvial
Nontron	NT	3056	Mount Sharp // Carolyn Shoemaker (faintly laminated mudstone)	lacustrine

Table S3. Oxygen isotopic compositions for water samples analyzed via a commercial PICARRO cavity ringdown spectrometer and a benchtop TLS at JPL. 'Calculated Vapor' values correspond to the $\delta^{18}O_{VSMOW}$ expected for water vapor in isotopic equilibrium at 25°C with liquid water having the $\delta^{18}O_{VSMOW}$ values in the 'PICARRO Measured Liquid' column. Uncertainties on the PICARRO liquid compositions were propagated forward to the calculated vapor compositions.

Identifier	*Expected* Liquid* $\delta^{18}O_{VSMOW}$ *(‰)*	*PICARRO Measured Liquid* † $\delta^{18}O_{VSMOW}$ *(‰)*	*PICARRO Calculated Vapor* † $\delta^{18}O_{VSMOW}$ *(‰)*	*JPL Benchtop TLS-Measured Vapor* ‡ $\delta^{18}O_{VSMOW}$ *± 1 SE (‰)*
MilliQ (DI tap Pasadena, CA)	–9	–9.1 ± 0.2	–18.3 ± 0.2	–33.7 ± 4.6
70:30 (DI:eBOC)	1.2	2.2 ± 0.2	–7.1 ± 0.2	–18.8 ± 2.5
eBOC	25	24.8 ± 0.3	15.3 ± 0.3	8.4 ± 2.8
60:40 (DI:SEW)	38	33.5 ± 0.7	23.9 ± 0.7	17.3 ± 3.5
50:50 (DI:SEW)	50	47.6 ± 0.9	37.8 ± 0.9	47.9 ± 1.8
40:60 (DI:SEW)	62	61.0 ± 1.2	51.1 ± 1.2	43.2 ± 3.5
20:80 (DI:SEW)	86	84.7 ± 1.4	74.6 ± 1.4	68.7 ± 2.1
SEW	110	111.2 ± 1.9	100.9 ± 1.9	137.8 ± 5.2

*Expected values for prepared samples were calculated prior to analysis based on volumetric mixing of endmembers given in parentheses.

†Uncertainties on PICARRO measurements represent combined uncertainty as calculated by the FLIIMP software (146).

‡TLS measurements are averages of $\delta^{18}O$ values calculated based on the B and F spectral lines; reported uncertainties were calculated by combining the associated errors in quadrature.

Table S4. Variables and associated numerical values used in modeling CO_2-H_2O oxygen exchange time constants. An asterisk (*) indicates that the reference provided the equation used to calculate numerical values.

Variable	*Symbol*	*Units*	*Values Used*	*Reference*
H_2O molar mass	-	g mol^{-1}	18.0153	NIST Chemistry WebBook, SRD 69 [*online*]
H_2O density	-	g cm^{-3}	273.16 K: 0.9998495<br><br>293.15 K: 0.9982067	CRC Handbook of Chemistry & Physics [*online*]
system temperature	T	K	273, 293	*bounding values from terrestrial systems*
total atmospheric pressure	P	atm	1, 1.5	Based on ranges from multiple papers: (151-159)
CO_2 partial pressure	pCO_2	atm	0.5, 1	
salinity	S_{salt}	--	0 (freshwater)<br><br>35 (terrestrial ocean)	*bounding values from terrestrial systems*
CO_2 volumetric solubility function	F_{CO2}	mol L^{-1}	273 K, S_{salt} = 0: 7.7823E-2<br><br>293 K, S_{salt} = 0: 3.8251E-2<br><br>273 K, S_{salt} = 35: 6.4406E-2<br><br>293 K, S_{salt} = 35: 3.2502E-2	Sarmiento & Gruber 2006* (149)<br><br>Coefficients from Weiss & Price 1980 (189)
H_2O saturation vapor pressure	pH_2O_{sat}	atm	273.16 K (SMOW): 6.0365E-3<br><br>293.15 K (SMOW): 2.3087E-3	CRC Handbook of Chemistry & Physics [*online*]<br><br>Wagner & Pruss 2002* (190)
CO_2 solubility parameter<br><br>(moist air, i.e., water-vapor saturated at air-water interface)	S_{CO2}	mmol m^{-3} atm^{-1}	273 K, S_{salt} = 0, P = 1: 7.7752E4<br><br>293 K, S_{salt} = 0, P = 1: 3.9154E4<br><br>273 K, S_{salt} = 35, P = 1: 6.4797E4<br><br>293 K, S_{salt} = 35, P = 1: 3.3270E4<br><br>273 K, S_{salt} = 0, P = 1.5: 5.1730E4<br><br>293 K, S_{salt} = 0, P = 1.5: 2.5899E4	Sarmiento & Gruber 2006* (149)

			273 K, S_{salt} = 35, P = 1.5: 4.3111E4<br><br>293 K, S_{salt} = 35, P = 1.5: 2.2007E4	
water boundary layer thickness	Δz_w	μm	30 (mean ocean)<br><br>100 (agitated, experimental)<br><br>300 (stagnant, experimental)	Broecker & Peng 1974 (162)
CO_2 molecular diffusivity in water	ε_w	$cm^2\ s^{-1}$	273 K: 9.7842E-6<br><br>293 K: 1.8007E-5	Zeebe 2011* (163)
lake mixed-layer depth	z_{ml}	m	10, 100, 1000	*order-of-magnitude bounding values*

Table S5. Temperature-dependent equations for Mars-relevant equilibrium mineral-water hydrogen and oxygen isotope fractionation. Water speciation for each mineral is given in parentheses: 'OH' refers to the bound hydroxyl group whereas 'H_2O' refers to structurally bound molecular water. Smectite and illite oxygen fractionation factors were determined via chemical methods that extract both the tetrahedrally (i.e., Si- and Al-bound) and octahedrally (i.e., the -OH) coordinated oxygen atoms within the clay framework and thus integrate over 'all structural O'. 'Method' refers to whether the calculated expression was determined via analyses of experimental results, natural samples, or calculated theoretically via *ab initio* methods. Temperatures are given in Celsius for convenience, but temperature (T) in all equations should be entered in Kevin. Equations are given in the 1000 ln α format (i.e., each expression is equated to 1000 times the natural logarithm of the fractionation factor). All alpha values are of the form $\alpha_{mineral–water}$.

**Oxygen Isotopes ($^{18}O/^{16}O$)**					
Mineral	*Method*	*T Range (°C)*	*Equation (= 1000 ln $\alpha_{mineral–water}$)*	*Reference*	*Notes*
smectite (all structural O)	natural and experimental	0–300	$2.55 \times 10^6 \times (1/T^2) - 4.05$	(191)	Data compilation
illite (all structural O)	natural and experimental	0–700	$2.39 \times 10^6 \times (1/T^2) - 3.76$	(191)	Data compilation
brucite (OH)	experimental	15–120	$1.56 \times 10^6 \times (1/T^2) - 14.1$	(192)	Calibrations at atmospheric pressure
goethite (OH)	natural and experimental	0–120	$1.66 \times 10^6 \times (1/T^2) - 12.6$	(193)	Over a pH range of 1–6
jarosite (OH)	experimental	100–250	$2.1 \times 10^6 \times (1/T^2) - 8.77$	(194)	Experiments showed between 40–100% oxygen isotope exchange
gypsum (H_2O)	experimental	3–40	$0.1177 \times 10^6 \times (1/T^2) + 2.134$	(173)	Salt effects at >150 g/L NaCl; data refit to proper functional form
gypsum (H_2O)	theoretical	0–60	$-0.4884 \times 10^6 \times (1/T^2) + 4.309 \times 10^3 \times (1/T) - 5.404$	(175)	Data from polynomial expression refit*
bassanite (H_2O)	theoretical	0–60	$-0.2667 \times 10^6 \times (1/T^2) + 2.342 \times 10^3 \times (1/T) - 1.301$	(175)	Data from polynomial expression refit*
**Hydrogen Isotopes (D/H)**					
Mineral	*Method*	*T Range (°C)*	*Value or Equation (= 1000 ln $\alpha_{mineral–water}$)*	*Reference*	*Notes*

montmorillonite smectite (OH)	natural samples	~0–5† (ocean sediments)	0.938	(178)	†Estimate; actual temperatures not reported
montmorillonite smectite (OH)	natural samples	~5–30†	0.970	(179)	†Estimate covering a range of weathering temperatures; clays were extracted from continental soils and have different chemical compositions than those in (178)
illite/smectite mixed clays (OH)	natural samples	95–150	$-45.3 \times 10^3 \times (1/T) + 94.7$	(180)	From geopressurized fluids
brucite (OH)	experimental	15–120	$-4.88 \times 10^6 \times (1/T^2) - 22.5$	(192)	Calibrations at atmospheric pressure
goethite (OH)	natural samples	22–48	0.900	(195)	No temperature dependence observed
jarosite (OH)	experimental	150	0.953	(194)	~89.5% hydrogen isotope exchange
jarosite (OH)	experimental	25	0.941	(196)	Value from fit to acid mine drainage jarosite precipitates
gypsum (H_2O)	experimental	3–40	$-1.477 \times 10^6 \times (1/T^2) - 2.534$	(173)	Salt effects at < 80 g/L NaCl; affected by crystallization kinetics at low T; data refit to proper functional form
gypsum (H_2O)	theoretical	0–60	$-14.92 \times 10^6 \times (1/T^2) + 84.72 \times 10^3 \times (1/T) - 136.4$	(175)	Data from polynomial expression refit to proper functional form*
bassanite (H_2O)	theoretical	0–60	$-19.69 \times 10^6 \times (1/T^2) + 99.29 \times 10^3 \times (1/T) - 147.1$	(175)	Data from polynomial expression refit to proper functional form*

*This approach led to oxygen alpha values an average of ~2E-6 (gypsum) and ~7E-7 (bassanite) greater than those calculated using the polynomial given in (175) and hydrogen alpha values an average of ~2E-5 (gypsum) and ~7E-5 (bassanite) greater than the same.

Table S6. Temperature-dependent equations for hydrogen and oxygen isotope fractionation between liquid water and water vapor. Temperatures ranges over which the equations are valid are given in Celsius for convenience, but temperature (*T*) in all equations is calculated in Kevin. Equations are given in the 1000 ln α format (i.e., each expression is equated to 1000 times the natural logarithm of the fractionation factor). All alpha values are of the form $\alpha_{liquid–vapor}$.

**Oxygen Isotopes**				
Method	*T Range (°C)*	*Equation (= 1000 ln $\alpha_{liq\text{-}vap}$)*	*Reference*	*Notes*
Experimental	0–100	$1.137 \times 10^6 \times (1/T^2) - 0.4156 \times 10^3 \times (1/T) - 2.0667$	(147)	Experimental results confirmed via theoretical treatment
**Hydrogen Isotopes**				
Method	*T Range (°C)*	*Equation (= 1000 ln $\alpha_{liq\text{-}vap}$)*	*Reference*	*Notes*
Experimental	0–100	$24.844 \times 10^6 \times (1/T^2) - 76.248 \times 10^3 \times (1/T) + 52.612$	(147)	Experimental results confirmed via theoretical treatment

Table S7. Constants used in the Craig-Gordon model calculation for evaporation into a low-humidity, CO_2-rich atmosphere. All calculations were performed for a temperature of 273 K and for two values of *h*: 0.05 (5% relative humidity) and 0.20 (20% relative humidity). Equilibrium fractionation factors ($\boldsymbol{\alpha_{eqm}}$) were calculated with the respective equations for vapor pressure isotope effects given in **Table S6**.

**Oxygen Isotopes ($^{18}O/^{16}O$)**			
Atmospheric Vapor (R_v)	*Initial Water Reservoir* (R_w^i)	α_{eqm}	α_{evap}^0
1.98191E-3	2.00517E-3	1.01174	1.03055
**Hydrogen Isotopes (D/H)**			
Atmospheric Vapor (R_v)	*Initial Water Reservoir* (R_w^i)	α_{eqm}	α_{evap}^0
4.54636-4	5.05809E-4	1.11256	1.13325

SI References

1. R. N. Clayton, T. K. Mayeda, Oxygen isotopes in eucrites, shergottites, nakhlites, and chassignites. *Earth and Planetary Science Letters* **62**, 1–6 (1983).
2. R. N. Clayton, T. K. Mayeda, Oxygen isotopes in Shergotty. *Geochimica et Cosmochimica Acta* **50**, 979–982 (1986).
3. R. N. Clayton, T. K. Mayeda, Oxygen isotope studies of achondrites. *Geochimica et Cosmochimica Acta* **60**, 1999–2017 (1996).
4. I. A. Franchi, I. P. Wright, A. S. Sexton, C. T. Pillinger, The oxygen-isotope composition of Earth and Mars. *Meteoritics & Planetary Science* **34**, 657–661 (1999).
5. C. K. Shearer, L. A. Leshin, C. T. Adcock, Olivine in martian meteorite Allan Hills 84001: Evidence for a high-temperature origin and implications for signs of life. *Meteoritics & Planetary Science* **34**, 331–339 (1999).
6. D. Rumble III, A. J. Irving (2009) Dispersion of oxygen isotopic compositions among 42 martian meteorites determined by laser fluorination: Evidence for assimilation of (ancient) altered crust. in *40th Lunar and Planetary Science Conference* (Houston, TX), p 2293.
7. M. B. Channon, E. M. Stolper, J. M. Eiler (2010) Oxygen isotope compositions of mineral separates from SNC meteorites: Constraints on SNC parental magmas. in *41st Lunar and Planetary Science Conference* (Houston, TX).
8. K. Ziegler, Z. D. Sharp, C. B. Agee (2013) The unique NWA 7034 martian meteorite: Evidence for multiple oxygen isotope reservoirs. in *44th Lunar and Planetary Science Conference* (Houston, TX).
9. A. Ali, I. Jabeen, D. Gregory, R. Verish, N. R. Banerjee, New triple oxygen isotope data of bulk and separated fractions from SNC meteorites: Evidence for mantle homogeneity of Mars. *Meteoritics & Planetary Science* **51**, 981–995 (2016).
10. J. M. Eiler, Oxygen isotope variations of basaltic lavas and upper mantle rocks. *Reviews in Mineralogy and Geochemistry* **43**, 319–364 (2001).
11. J. P. Greenwood, R. E. Blake, C. D. Coath, Ion microprobe measurements of $^{18}O/^{16}O$ ratios of phosphate minerals in the martian meteorites ALH84001 and Los Angeles. *Geochimica et Cosmochimica Acta* **67**, 2289–2298 (2003).
12. J. J. Belluci *et al.*, Tracing martian surface interactions with the triple O isotope compositions of meteoritic phosphates. *Earth and Planetary Science Letters* **531**, 115977 (2020).
13. J. W. Valley *et al.*, Low-temperature carbonate concretions in the martian meteorite ALH84001: Evidence from stable isotopes and mineralogy. *Science* **275**, 1633–1638 (1997).
14. L. A. Leshin, K. D. McKeegan, P. K. Carpenter, R. P. Harvey, Oxygen isotope constraints on the genesis of carbonates from martian meteorite ALH84001. *Geochimica et Cosmochimica Acta* **62**, 3–13 (1998).
15. J. M. Saxton, I. C. Lyon, G. Turner, Correlated chemical and isotopic zoning in carbonates in the martian meteorite ALH84001. *Earth and Planetary Science Letters* **160**, 811–822 (1998).

16. J. M. Saxton, I. C. Lyon, E. Chatzitheodoridis, G. Turner, Oxygen isotopic composition of carbonate in the Nakhla meteorite: Implications for the hydrosphere and atmosphere of Mars. *Geochimica et Cosmochimica Acta* **64**, 1299–1309 (2000).
17. J. M. Eiler, J. W. Valley, C. M. Graham, J. Fournelle, Two populations of carbonate in ALH84001: Geochemical evidence for discrimination and genesis. *Geochimica et Cosmochimica Acta* **66**, 1285–1303 (2002).
18. G. Holland, J. M. Saxton, I. C. Lyon, G. Turner, Negative $\delta^{18}O$ values in Allan Hills 84001 carbonate: Possible evidence for water precipitation on Mars. *Geochimica et Cosmochimica Acta* **69**, 1359–1369 (2005).
19. I. P. Wright, M. M. Grady, C. T. Pillinger, Carbon, oxygen, and nitrogen isotopic compositions of possible martian weathering products in EETA 79001. *Geochimica et Cosmochimica Acta* **52**, 917–924 (1988).
20. I. P. Wright, M. M. Grady, C. T. Pillinger, Chassigny and the nakhlites: Carbon-bearing components and their relationship to martian environmental conditions. *Geochimica et Cosmochimica Acta* **56**, 817–826 (1992).
21. C. S. Romanek *et al.*, Record of fluid-rock interactions on Mars from the meteorite ALH84001. *Nature* **372**, 655–657 (1994).
22. A. J. T. Jull, C. J. Eastoe, S. Xue, G. F. Herzog, Isotopic composition of carbonates in the SNC meteorites Allan Hills 84001 and Nakhla. *Meteoritics* **30**, 311–318 (1995).
23. A. J. T. Jull, C. J. Eastoe, S. Cloudt, Isotopic composition of carbonates in the SNC meteorites, Allan Hills 84001 and Zagami. *Journal of Geophysical Research* **102**, 1663–1669 (1997).
24. J. Farquhar, M. H. Thiemens, T. L. Jackson, Atmosphere-surface interactions on Mars: $\Delta^{17}O$ measurements of carbonate from ALH 84001. *Science* **280**, 1580–1582 (1998).
25. J. Farquhar, M. H. Thiemens, Oxygen cycle of the martian atmosphere-regolith system: $\Delta^{17}O$ of secondary phases in Nakhla and Lafayette. *Journal of Geophysical Research* **105**, 11991–11997 (2000).
26. I. Halevy, W. W. Fischer, J. M. Eiler, Carbonates in the martian meteorite Allan Hills 84001 formed at 18 ± 4°C in a near-surface aqueous environment. *Proceedings of the National Academy of Sciences USA* **108**, 16895–16899 (2011).
27. R. Shaheen, P. B. Niles, K. Chong, C. M. Corrigan, M. H. Thiemens, Carbonate formation events in ALH 84001 trace the evolution of the martian atmosphere. *Proceedings of the National Academy of Sciences USA* **112**, 336–341 (2015).
28. H. R. Karlsson, R. N. Clayton, E. K. Gibson Jr, T. K. Mayeda, Water in SNC meteorites: Evidence for a martian hydrosphere. *Science* **255**, 1409–1411 (1992).
29. L. A. Leshin, S. Epstein, E. M. Stolper, Hydrogen isotope geochemistry of SNC meteorites. *Geochimica et Cosmochimica Acta* **60**, 2635–2650 (1996).
30. C. B. Agee *et al.*, Unique meteorite from early Amazonian Mars: Water-rich basaltic breccia Northwest Africa 7034. *Science* **339**, 780–785 (2013).
31. O. V. Maltsev (2017) Oxygen isotope composition of water in martian meteorites. M.S. in *Earth and Planetary Sciences* (University of New Mexico).

32. G. L. Bjoraker, M. J. Mumma, H. P. Larson (1989) Isotopic abundance ratios for hydrogen and oxygen in the martian atmosphere. in *21st Annual Division of Planetary Sciences (DPS) Meeting*, p 991.
33. S. M. F. Sheppard, "Characterization and Isotopic Variations in Natural Waters" in Stable Isotopes in High Temperature Geological Processes*,* P. H. Ribbe, Ed. (Mineralogical Society of America, Washinton, D.C., 1986), vol. 16, chap. 6, pp. 165–183.
34. R. E. Criss, H. P. Taylor Jr., "Meteoric-Hydrothermal Systems" in Stable Isotopes in High Temperature Geological Processes*,* P. H. Ribbe, Ed. (Mineralogical Society of America, Washington, D.C., 1986), vol. 16, chap. 11, pp. 373–424.
35. M.-C. Liang, S. Mahata, A. H. Laskar, M. H. Thiemens, S. Newman, Oxygen isotope anomaly in tropospheric CO_2 and implications for CO_2 residence time in the atmosphere and gross primary productivity. *Scientific Reports* **7**, 13180 (2017).
36. M. H. Thiemens, S. Chakraborty, T. L. Jackson, Decadal $\Delta^{17}O$ record of tropospheric CO_2: Verification of a stratospheric component in the troposphere. *Journal of Geophysical Research: Atmospheres* **119**, 6221–6229 (2014).
37. A. O. Nier, M. B. McElroy, Y. L. Yung, Isotopic composition of the martian atmosphere. *Science* **194**, 68–70 (1976).
38. P. B. Niles, W. V. Boynton, J. H. Hoffman, D. W. Ming, D. Hamara, Stable isotope measurements of the martian atmospheric CO_2 at the Phoenix landing site. *Science* **329**, 1334–1337 (2010).
39. C. R. Webster *et al.*, Isotope ratios of H, C, and O in CO_2 and H_2O of the martian atmosphere. *Science* **341**, 260–263 (2013).
40. H. B. Franz *et al.*, Indigenous and exogenous organics and surface-atmosphere cycling inferred from carbon and oxygen isotopes at Gale crater. *Nature Astronomy* **4**, 526–532 (2020).
41. C. S. Romanek *et al.*, Oxygen isotopic record of silicate alteration in the Shergotty-Nakhla-Chassigny meteorite Lafayette. *Meteoritics and Planetary Science* **33**, 775–784 (1998).
42. A. A. Nemchin *et al.*, Record of the ancient martian hydrosphere and atmosphere preserved in zircon from a martian meteorite. *Nature Geoscience* **7**, 638–642 (2014).
43. R. N. Clayton, T. K. Mayeda, Isotopic composition of carbonate in EETA79001 and its relation to parent body volatiles. *Geochimica et Cosmochimica Acta* **52**, 925–927 (1988).
44. J. Alday *et al.*, Oxygen isotope ratios in martian water vapor observed by ACS MIR on board the ExoMars Trace Gas Orbiter. *Astronomy & Astrophysics* **630**, A91 (2019).
45. J. Alday *et al.*, Constraining the global composiiton of D/H and $^{18}O/^{16}O$ in Martian water using SOFIA/EXES. *Monthly Notices of the Royal Astronomical Society* **530**, 2919–2932 (2024).
46. J. L. Fox, A. Hác, Isotope fractionation in the photochemical escape from Mars. *Icarus* **208**, 176–191 (2010).
47. Y. Chen *et al.*, Evidence in Tissint for recent subsurface water on Mars. *Earth and Planetary Science Letters* **425**, 55–63 (2015).

48. N. Z. Boctor, C. M. O. D. Alexander, J. Wang, E. Hauri, The sources of water in martian meteorites: Clues from hydrogen isotopes. *Geochimica et Cosmochimica Acta* **67**, 3971–3989 (2003).
49. T. Usui, C. M. O. D. Alexander, J. Wang, J. I. Simon, J. H. Jones, Origin of water and mantle-crust interactions on Mars inferred from hydrogen isotopes and volatile element abundances of olivine-hosted melt inclusions of primitive shergottites. *Earth and Planetary Science Letters* **357–358**, 119–129 (2012).
50. T. Usui, C. M. O. D. Alexander, J. Wang, J. I. Simon, J. H. Jones, Meteoritic evidence for a previously unrecognized hydrogen reservoir on Mars. *Earth and Planetary Science Letters* **410**, 140–151 (2015).
51. Y. Liu *et al.*, Impact-melt hygrometer for Mars: The case of shergottite Elephant Moraine (EETA) 79001. *Earth and Planetary Science Letters* **490**, 206–215 (2018).
52. A. H. Peslier *et al.*, Determination of the water content and D/H ratio of the martian mantle by unraveling degassing and crystallization effects in nakhlites. *Geochimica et Cosmochimica Acta* **266**, 382–415 (2019).
53. J. J. Barnes *et al.*, Multiple early-formed water reservoirs in the interior of Mars. *Nature Geoscience* **13**, 260–264 (2020).
54. S. Aoki *et al.*, Seasonal variation of the HDO/H_2O ratio in the atmosphere of Mars at the middle of northern spring and the beginning of northern summer. *Icarus* **260**, 7–22 (2015).
55. T. Encrenaz *et al.*, New measurements of D/H on Mars using EXES aboard SOFIA. *Astronomy & Astrophysics* **612**, A112 (2018).
56. A. S. J. Khayat, G. L. Villanueva, M. D. Smith, S. D. Guzewich, IRTF/CSHELL mapping of atmospheric HDO, H_2O, and D/H on Mars during northern summer. *Icarus* **330**, 204–216 (2019).
57. V. A. Krasnopolsky, Variations of the HDO/H_2O ratio in the martian atmosphere and loss of water from Mars. *Icarus* **257**, 377–386 (2015).
58. R. E. Novak, M. J. Mumma, G. L. Villanueva, Measurement of the isotopic signatures of water on Mars: Implications for studying methane. *Planetary and Space Science* **59**, 163–168 (2011).
59. T. Owen, J. P. Maillard, C. de Bergh, B. L. Lutz, Deuterium on Mars: The abundance of HDO and the value of D/H. *Science* **240**, 1767–1770 (1988).
60. A. C. Vandaele *et al.*, Martian dust storm impact on atmospheric H_2O and D/H observed by ExoMars Trace Gas Orbiter. *Nature* **568**, 521–525 (2019).
61. G. L. Villanueva *et al.*, Strong water isotopic anomalies in the martian atmosphere: Probing current and ancient reservoirs. *Science* **348**, 218–221 (2015).
62. G. L. Villanueva *et al.*, Water heavily fractionated as it ascends on Mars as revealed by ExoMars/NOMAD. *Science Advances* **7**, eabc8843 (2021).
63. G. L. Villanueva *et al.*, The deuterium isotope ratio of water released from the martian caps as measured with TGO/NOMAD. *Geophysical Research Letters* **49**, e2022GL098161 (2022).
64. B. J. Thomson *et al.*, Constraints on the origin and evolution of the layered mound in Gale crater, Mars using Mars Reconnaissance Orbiter data. *Icarus* **214**, 413–432 (2011).

65. L. Le Deit *et al.*, Sequence of infilling events in Gale crater, Mars: Results from morphology, stratigraphy, and mineralogy. *Journal of Geophysical Research: Planets* **118**, 2439–2473 (2013).
66. K. A. Farley *et al.*, In situ radiometric and exposure age dating of the martian surface. *Science* **343**, 1247166 (2014).
67. R. B. Anderson, J. F. Bell, III, Geologic mapping and characterization of Gale crater and implications for its potential as a Mars Science Laboratory landing site. *Mars* **5**, 76–128 (2010).
68. M. C. Palucis *et al.*, The origin and evolution of the Peace Vallis fan system that drains to the Curiosity landing area, Gale crater, Mars. *Journal of Geophysical Research: Planets* **119**, 705–728 (2014).
69. R. M. E. Williams *et al.*, Martian fluvial conglomerates at Gale crater. *Science* **340**, 1068–1072 (2013).
70. M. C. Palucis *et al.*, Sequence and relative timing of large lakes in Gale crater (Mars) after the formation of Mount Sharp. *Journal of Geophysical Research: Planets* **121**, 472–496 (2016).
71. J. A. Grant, S. A. Wilson, N. Mangold, F. Calef III, J. P. Grotzinger, The timing of alluvial activity in Gale crater, Mars. *Geophysical Research Letters* **41**, 1142–1148 (2014).
72. J. P. Grotzinger *et al.*, Deposition, exhumation, and paleoclimate of an ancient lake deposit, Gale crater, Mars. *Science* **350**, aac7575 (2015).
73. J. P. Grotzinger *et al.*, A habiable fluvio-lacustrine environment at Yellowknife Bay, Gale crater, Mars. *Science* **343**, 1242777 (2014).
74. R. E. Milliken, J. P. Grotzinger, B. J. Thomson, Paleoclimate of Mars as captured by the stratigraphic record in Gale crater. *Geophysical Research Letters* **37**, L04201 (2010).
75. A. A. Fraeman *et al.*, A hematite-bearing layer in Gale crater, Mars: Mapping and implications for past aqueous conditions. *Geology* **41**, 1103–1106 (2013).
76. E. B. Rampe *et al.*, Mineralogy and geochemistry of sedimentary rocks and eolian sediments in Gale crater, Mars: A review after six Earth years of exploration with Curiosity. *Geochemistry* **80**, 125605 (2020).
77. S. G. Banham *et al.*, Ancient Martian aeolian processes and palaeomorphology reconstructed from the Stimson formation on the lower slop of Aeolis Mons, Gale crater, Mars. *Sedimentology* **65**, 993–1042 (2018).
78. R. E. Milliken, R. C. Ewing, W. W. Fischer, J. Hurowitz, Wind-blown sandstones cemented by sulfate and clay minerals in Gale crater, Mars. *Geophysical Research Letters* **41**, 1149–1154 (2014).
79. D. F. Blake *et al.*, Curiosity at Gale crater, Mars: Characterization and analysis of the Rocknest sand shadow. *Science*, 1239505 (2013).
80. E. B. Rampe *et al.*, Sand mineralogy within the Bagnold Dunes, Gale crater, as observe in situ and from orbit. *Geophysical Research Letters* **45**, 9488–9497 (2018).
81. K. M. Stack *et al.*, Evidence for plunging river plume deposits in the Pahrump Hills member of the Murray formation, Gale crater, Mars. *Sedimentology* **66**, 1768–1802 (2019).

82. L. A. Edgar *et al.*, A lacustrine paleoenvironment recorded at Vera Rubin Ridge, Gale crater: Overview of the sedimentology and stratigraphy observed by the Mars Science Laboratory Curiosity rover. *Journal of Geophysical Research: Planets* **125**, e2019JE006307 (2020).
83. E. B. Rampe *et al.*, Mineralogy of an ancient lacustrine mudstone succession from the Murray formation, Gale crater, Mars. *Earth and Planetary Science Letters* **471**, 172–185 (2017).
84. D. L. Blaney *et al.*, Chemistry and texture of the rocks at Rocknest, Gale crater: Evidence for sedimentary origin and diagenetic alteration. *Journal of Geophysical Research: Planets* **119**, 2109–2131 (2014).
85. J. C. Bridges *et al.*, Diagenesis and clay mineral formation at Gale crater, Mars. *Journal of Geophysical Research: Planets* **120**, 1–19 (2014).
86. R. J. Léveillé *et al.*, Chemistry of fracture-filled raised ridges in Yellowknife Bay, Gale crater: Window into past aqueous activity and habitability on Mars. *Journal of Geophysical Research: Planets* **119**, 2398–2415 (2014).
87. M. Nachon *et al.*, Calcium sulfate veins characterized by ChemCam/Curiosity at Gale crater, Mars. *Journal of Geophysical Research: Planets* **119**, 1991–2016 (2014).
88. K. L. Siebach *et al.*, Subaqueous shrinkage cracks in the Sheepbed mudstone: Implications for early fluid diagenesis, Gale crater, Mars. *Journal of Geophysical Research: Planets* **119**, 1597–1613 (2014).
89. K. M. Stack *et al.*, Diagenetic origin of nodules in the Sheepbed member, Yellowknife Bay formation, Gale crater, Mars. *Journal of Geophysical Research: Planets* **119**, 1637–1664 (2014).
90. S. P. Schwenzer *et al.*, Fluids during diagenesis and sulfate vein formation in sediments at Gale crater, Mars. *Meteoritics & Planetary Science* **51**, 2175–2202 (2016).
91. A. H. Treiman *et al.*, Mineralogy, provenance, and diagenesis of a potassic basaltic sandstone on Mars: CheMin X-ray diffraction of the Windjana sample (Kimberley area, Gale crater). *Journal of Geophysical Research: Planets* **121**, 75–106 (2016).
92. J. Frydenvang *et al.*, Diagenetic silica enrichment and late-stage groundwater activity in Gale crater, Mars. *Geophysical Research Letters* **44**, 4716–4724 (2017).
93. P. E. Martin *et al.*, A two-step K-Ar experiment on Mars: Dating the diagenetic formation of jarosite from Amazonian groundwater. *Journal of Geophysical Research: Planets* **122**, 2803–2818 (2017).
94. M. Nachon *et al.*, Chemistry of diagenetic features analyzed by ChemCam at Pahrump Hills, Gale crater, Mars. *Icarus* **281**, 121–136 (2017).
95. A. S. Yen *et al.*, Multiple stages of aqueous alteration along fractures in mudstone and sandstone strata in Gale crater, Mars. *Earth and Planetary Science Letters* **471**, 186–198 (2017).
96. C. C. Bedford *et al.*, Alteration trends and geochemical source region characteristics preserved in the fluviolacustrine sedimentary record of Gale crater, Mars. *Geochimica et Cosmochimica Acta* **246**, 234–266 (2019).

97. R. E. Kronyak *et al.*, Mineral-filled fractures as indicators of multigenerational fluid flow in the Pahrump Hills member of the Murray formation, Gale crater, Mars. *Earth and Space Science* **6**, 238–265 (2019).
98. R. E. Kronyak *et al.*, Extensive polygonal fracture network in Siccar Point group strata: Fracture mechanisms and implications for fluid circulation in Gale crater, Mars. *Journal of Geophysical Research: Planets* **124**, 2613–2634 (2019).
99. N. Mangold *et al.*, Chemical alteration of fine-grained sedimentary rocks at Gale crater. *Icarus* **321**, 619–631 (2019).
100. C. N. Achilles *et al.*, Evidence for multiple diagenetic episodes in ancient fluvial-lacustrine sedimentary rocks in Gale crater, Mars. *Journal of Geophysical Research: Planets* **125**, e2019JE006295 (2020).
101. A. A. Fraeman *et al.*, Evidence for a diagenetic origin of Vera Rubin Ridge, Gale crater, Mars: Summary and synethesis of Curiosity's exploration campaign. *Journal of Geophysical Research: Planets* **125**, e2020JE006527 (2020).
102. K. A. Bennett *et al.*, Diagenesis revealed by fine-scale features at Vera Rubin Ridge, Gale crater, Mars. *Journal of Geophysical Research: Planets* **126**, e2019JE006311 (2021).
103. P. J. Gasda *et al.*, In situ detection of boron by ChemCam on Mars. *Geophysical Research Letters* **44**, 8739–8748 (2017).
104. N. Stein *et al.*, Desiccation cracks provide evidence of lake drying on Mars, Sutton Island member, Murray formation, Gale crater. *Geology* **46**, 515–518 (2018).
105. W. Rapin *et al.*, An interval of high salinity in ancient Gale crater lake on Mars. *Nature Geoscience* **12**, 889–895 (2019).
106. N. H. Thomas *et al.*, Mars Science Laboratory observations of chloride salts in Gale crater, Mars. *Geophysical Research Letters* **46**, 10754–10763 (2019).
107. J. A. Hurowitz *et al.*, Redox stratification of an ancient lake in Gale crater, Mars. *Science* **356**, eaah6849 (2017).
108. T. F. Bristow *et al.*, Clay mineral diversity and abundance in sedimentary rocks of Gale crater, Mars. *Science Advances* **4**, eaar3330 (2018).
109. W. Rapin *et al.*, Sustained wet-dry cycling on early Mars. *Nature* **620**, 299–302 (2023).
110. T. F. Bristow *et al.*, Brine-driven destruction of clay minerals in Gale crater, Mars. *Science* **373**, 198–204 (2021).
111. P. J. Gasda *et al.*, Overview of the morphology and chemistry of diagenetic features in the clay-rich Glen Torridon unit of Gale crater, Mars. *Journal of Geophysical Research: Planets* **127**, e2021JE007097 (2022).
112. M. T. Thorpe *et al.*, Mars Science Laboratory CheMin data from the Glen Torridon region and the significance of lake-groundwater interactions in interpreting mineralogy and sedimentary history. *Journal of Geophysical Research: Planets* **127**, e2021JE007099 (2022).
113. J. Meija *et al.*, Isotopic compositions of the elements 2013 (IUPAC Technical Report). *Pure and Applied Chemistry* **88**, 293–306 (2016).
114. L. A. Leshin *et al.*, Volatile, isotope, and organic analysis of martian fines with the Mars Curiosity rover. *Science* **341**, 1238937 (2013).

115. B. Sutter *et al.*, Evolved gas analyses of sedimentary rocks and eolian sediment in Gale crater, Mars: Results of the Curiosity rover's sample analysis at Mars instrument from Yellowknife Bay to the Namib Dune. *Journal of Geophysical Research: Planets* **122**, 2574–2609 (2017).
116. C. N. Achilles *et al.*, Mineralogy of an active eolian sediment from the Namib dune, Gale crater, Mars. *Journal of Geophysical Research: Planets* **122**, 2344–2361 (2017).
117. S. M. Morrison *et al.*, Crystal chemistry of martian minerals from Bradbury Landing through Naukluft Plateau, Gale crater, Mars. *American Mineralogist* **103**, 857–871 (2018).
118. E. Dehouck, S. M. McLennan, P.-Y. Meslin, A. Cousin, Constraints on abundance, composition, and nature of X-ray amorphous components of soils and rocks at Gale crater, Mars. *Journal of Geophysical Research: Planets* **119**, 2640–2657 (2014).
119. S. Hu *et al.*, NanoSIMS analyses of apatite and melt inclusions in the GRV 020090 Martian meteorite: Hydrogen isotope evidence for recent past underground hydrothermal activity on Mars. *Geochimica et Cosmochimica Acta* **140**, 321–333 (2014).
120. R. C. Anderson *et al.*, Collecting samples in Gale crater, Mars; an overview of the Mars Science Laboratory Sample Acquisition, Sample Processing and Handling System. *Space Science Reviews* **170**, 57–75 (2012).
121. M. Clog, D. Stolper, J. M. Eiler, Kinetics of CO_2(g)–H_2O(l) isotopic exchange, including mass 47 isotopologues. *Chemical Geology* **395**, 1–10 (2015).
122. C. A. M. Brenninkmeijer, P. Kraft, W. G. Mook, Oxygen isotope fractionation between CO_2 and H_2O. *Isotope Geoscience* **1**, 181–190 (1983).
123. J. Bigeleisen, Statistical mechanics of isotope effects on the thermodynamic properties of condensed systems. *The Journal of Chemical Physics* **34**, 1485–1493 (1961).
124. W. A. Van Hook, Isotope effects on vaporization from the adsorbed state. The methane system. *Journal of Physical Chemistry* **71**, 3270–3275 (1967).
125. T. Richard, L. Mercury, M. Massault, J.-L. Michelot, Experimental study of D/H isotopic fractionation factor of water adsorbed on porous silica tubes. *Geochimica et Cosmochimica Acta* **71**, 1159–1169 (2007).
126. E. Oerter *et al.*, Oxygen isotope fractionation effects in soil water via interaction with cations (Mg, Ca, K, Na) adsorbed to phyllosilicate clay minerals. *Journal of Hydrology* **515**, 1–9 (2014).
127. Y. Lin, J. Horita, An experimental stufy on isotope fractionation in a mesoporous silica-water system with implications for vadose-zone hydrology. *Geochimica et Cosmochimica Acta* **184**, 257–271 (2016).
128. Y. Lin, J. Horita, O. Abe, Adsorption isotope effects of water on mesoporous silica and alumina with implications for the land-vegetation-atmosphere system. *Geochimica et Cosmochimica Acta* **223**, 520–536 (2017).
129. J. Jänchen, R. V. Morris, D. L. Bish, M. Janssen, U. Hellwig, The H_2O and CO_2 adsorption properties of phyllosilicate-poor palagonitic dust and smectites under martian environmental conditions. *Icarus* **200**, 463–467 (2009).

130. P. Richet, Y. Bottinga, M. Javoy, A review of hydrogen, carbon, nitrogen, oxygen, sulphur, and chlorine stable isotope fractionation among gaseous molecules. *Annual Review of Earth and Planetary Sciences* **5**, 65–110 (1977).
131. M. S. Hamza, S. Epstein, Oxygen isotope fractionation between oxygen of different sites in hydroxyl-bearing silicate minerals. *Geochimica et Cosmochimica Acta* **44**, 173–182 (1980).
132. X. Feng, S. M. Savin, Oxygen isotope studies of zeolites—Stilbite, analcime, heulandite, and clinoptilolote: III. Oxygen isotope fractionation between stilbite and water or water vapor. *Geochimica et Cosmochimica Acta* **57**, 4239–4247 (1993).
133. J.-P. Girard, S. M. Savin, Intracrystalline fractionation of oxygen isotopes between hydroxyl and non-hydroxyl sites in kaolinite measured by thermal dehydroxylation and partial fluorination. *Geochimica et Cosmochimica Acta* **60**, 469–487 (1996).
134. D. W. Ming *et al.*, Volatile and organic composiitons of sedimentary rocks in Yellowknife Bay, Gale crater, Mars. *Science* **6169**, 1245267 (2014).
135. J. C. Stern *et al.*, Organic carbon concentrations in 3.5-billion-year-old lacustrine mudstones of Mars. *Proceedings of the National Academy of Sciences USA* **119**, e2201139119 (2022).
136. D. P. Glavin *et al.*, Evidence for perchlorates and the origin of chlorinated hydrocarbons detected by SAM at the Rocknest aeolian deposit in Gale crater. *Journal of Geophysical Research: Planets* **118**, 1955–1973 (2013).
137. C. H. House *et al.*, Depleted carbon isotope compositions observed at Gale crater, Mars. *Proceedings of the National Academy of Sciences USA* **119**, e2115651119 (2022).
138. K. Peters, M. Schoell, ^{13}C-depleted methane pyrolyzed from contaminated Gale Crater sediment cores, Mars. *Icarus* **410**, 115890 (2024).
139. C. Freissinet *et al.*, Organic molecules in the Sheepbed Mudstone, Gale crater, Mars. *Journal of Geophysical Research: Planets* **120**, 495–514 (2015).
140. J. L. Eigenbrode *et al.*, Organic matter preserved in 3-billion-year-old mudstones at Gale crater, Mars. *Science* **360**, 1096–1101 (2018).
141. P. R. Mahaffy *et al.*, The Sample Analysis at Mars investigation and instrument suite. *Space Science Reviews* **170**, 401–478 (2012).
142. P. R. Mahaffy *et al.*, The imprint of atmospheric evolution in the D/H of Hesperian clay minerals on Mars. *Science* **6220**, 412–414 (2015).
143. L. E. Gordon *et al.*, The HITRAN2016 molecular spectroscopic database. *Journal of Quantitative Spectroscopy and Radiative Transfer* **203**, 3–69 (2017).
144. J. Manne, C. R. Webster, Determination of spectral parameters for lines targeted by the Tunable Laser Spectrometer (TLS) on the Mars Curiosity rover. *Journal of Quantitative Spectroscopy and Radiative Transfer* **171**, 28–38 (2016).
145. G. J. Bowen, J. R. Ehleringer, L. A. Chesson, E. Stange, T. E. Cerling, Stable isotope ratios of tap water in the contiguous United States. *Water Resources Research* **43**, W03419 (2007).
146. H. Sodemann, P. T. Mørkved, S. Wahl, FLIIMP – a community software for the processing, calibration, and reporting of liquid water isotope measurements on cavity-ring down spectrometers. *MethodsX* **11**, 102297 (2023).

147. M. Majoube, Fractionnement en oxygène 18 at en deutérium entre l'eau et sa vapeur. *Journal de chimie physique et de physico-chimie biologique* **68**, 1423–1436 (1971).
148. J. Browaeys (2024) Linear fit with both uncertainties in x and in y. (MATLAB Central File Exchange).
149. J. L. Sarmiento, N. Gruber, *Ocean Biogeochemical Dynamics* (Princeton University Press, Princeton, NJ, 2006), pp. 528.
150. P. S. Liss, P. G. Slater, Flux of gases across the air-sea interface. *Nature* **247**, 181–184 (1974).
151. E. S. Kite, J.-P. Williams, A. Lucas, O. Aharonson, Low palaeopressure of the martian atmosphere estimated from the size distribution of ancient craters. *Nature Geoscience* **7**, 335–338 (2014).
152. R. Hu, D. M. Kass, B. L. Ehlmann, Y. L. Yung, Tracing the fate of carbon and the atmospheric evolution of Mars. *Nature Communications* **6**, 10003 (2015).
153. U. V. Amerstorfer *et al.*, Escape and evolution of Mars's CO_2 atmosphere: Influence of suprathermal atoms. *Journal of Geophysical Research: Planets* **122**, 1321–1337 (2017).
154. R. M. Ramirez, A warmer and wetter solution for early Mars and the challenges with transient warming. *Icarus* **297**, 71–82 (2017).
155. P. J. Godin *et al.*, Collision-induced absorption of CH_4-CO_2 and H_2-CO_2 complexes and their effect on ancient martian atmosphere. *Journal of Geophysical Research: Planets* **125**, e2019JE006357 (2020).
156. R. M. Ramirez, R. A. Craddock, T. Usui, Climate simulations of early Mars with estimated precipitation, runoff, and erosion rates. *Journal of Geophysical Research: Planets* **125**, e2019JE006160 (2020).
157. R. Wordsworth *et al.*, A coupled model of episodic warming, oxidation and geochemical transitions on early Mars. *Nature Geoscience* **14**, 127–132 (2021).
158. R. Hu, T. B. Thomas, A nitrogen-rich atmosphere on ancient Mars consistent with isotopic evolution models. *Nature Geoscience* **15**, 106–111 (2022).
159. E. S. Kite *et al.*, Changing spatial distribution of water flow charts major change in Mars's greenhouse effect. *Science Advances* **8**, eabo5894 (2022).
160. S. M. McLennan *et al.*, Elemental geochemistry of sedimentary rocks at Yellowknife Bay, Gale crater, Mars. *Science* **343**, 1244734 (2014).
161. D. T. Vaniman *et al.*, Mineralogy of a mudstone at Yellowknife Bay, Gale crater, Mars. *Science* **343**, 1243480 (2014).
162. W. S. Broecker, T.-H. Peng, Gas exchange rates between air and sea. *Tellus* **26**, 21–35 (1974).
163. R. E. Zeebe, On the molecular diffusion coefficients of dissolved CO_2, HCO_3^-, and CO_3^{2-} and their dependence on isotopic mass. *Geochimica et Cosmochimica Acta* **75**, 2483–2498 (2011).
164. B. Jähne, G. Heinz, W. Dietrich, Measurement of the diffusion coefficients of sparingly soluble gases in water. *Journal of Geophysical Research* **10**, 10767–10776 (1987).
165. D. M. Kern, The hydration of carbon dioxide. *Journal of Chemical Education* **37**, 14–23 (1960).

166. R. F. Miller, D. C. Berkshire, J. J. Kelley, D. W. Hood, Method for determination of reaction rates of carbon dioxide with water and hydroxyl ion in seawater. *Environmental Science & Technology* **5**, 127–133 (1971).
167. K. S. Johnson, Carbon dioxide hydration and dehydration kinetics in seawater. *Limnology and Oceanography* **27**, 849–855 (1982).
168. A. L. Soli, R. H. Byrne, CO_2 system hydration and dehydration kinetics and the equilibrium CO_2/H_2CO_3 ratio in aqueous NaCl solution. *Marine Chemistry* **78**, 65–73 (2002).
169. J. M. Watkins, L. C. Nielsen, F. J. Ryerson, D. J. DePaolo, The influence of kinetics on the oxygen isotope composition of calcium carbonate. *Earth and Planetary Science Letters* **375**, 349–360 (2013).
170. D. York, N. M. Evensen, M. López-Martínez, Unified equations for the slope, intercept, and standard errors of the best straight line. *American Journal of Physics* **72**, 367–375 (2004).
171. A. Bechtel, S. Hoernes, Oxygen isotope fractionation between oxygen of different sites in illite minerals: a potential single-mineral thermometer. *Contributions to Mineralogy and Petrology* **104**, 463–470 (1990).
172. H. C. Urey, The thermodynamic properties of isotopic substances. *Journal of the Chemical Society* 10.1039/JR9470000562, 562–581 (1947).
173. F. Gázquez, N. P. Evans, D. A. Hodell, Precise and accurate isotope fractionation factors ($\alpha^{17}O$, $\alpha^{18}O$, and αD) for water and $CaSO_4 \cdot 2H_2O$ (gypsum). *Geochimica et Cosmochimica Acta* **198**, 259–270 (2017).
174. Y. Bottinga, M. Javoy, Comments on oxygen isotope geothermometry. *Earth and Planetary Science Letters* **20**, 250–265 (1973).
175. T. Liu, E. Artacho, F. Gázquez, G. Walters, D. A. Hodell, Prediction of equilibrium isotopic fractionation of the gypsum/bassanite/water system using first-principles calculations. *Geochimica et Cosmochimica Acta* **244**, 1–11 (2019).
176. I. Friedman, J. R. O'Neil, "Compilation of stable isotope fractionation factors of geochemical interest" in Data of Geochemistry, Sixth Edition, M. Fleischer, Ed. (United States Geological Survey, Washinton, D.C., 1977), chap. KK.
177. E. B. Rampe *et al.*, Evidence for partially chloritized smectite in Gale crater, Mars. *Clays and Clay Minerals* **73**, 1–21 (2025).
178. S. M. Savin, S. Epstein, The oxygen and hydrogen isotope geochemistry of clay minerals. *Geochimica et Cosmochimica Acta* **34**, 25–42 (1970).
179. J. R. Lawrence, H. P. Taylor Jr., Deuterium and oxygen-18 correlation: Clay minerals and hydroxides in Quaternary soils compared to meteoric waters. *Geochimica et Cosmochimica Acta* **35**, 993–1003 (1971).
180. R. M. Capuano, The temperature dependence of hydrogen isotope fractionation between clay minerals and water: Evidence from a geopressured system. *Geochimica et Cosmochimica Acta* **56**, 2547–2554 (1992).
181. J. Horita, K. Rozanski, S. Cohen, Isotope effects in the evaporation of water: a status report of the Craig-Gordon model. *Isotopes in Environmental and Health Studies* **44**, 23–49 (2008).
182. G. M. Martínez *et al.*, The modern near-surface martian climate: A review of in-situ meteorological data from Viking to Curiosity. *Space Science Reviews* **212**, 295–338 (2017).

183. J. V. Clark *et al.*, High-temperature HCl evolutions from mixtures of perchlorates and chlorides with water-bearing phases: Implications for the Sample Analysis at Mars (SAM) instrument in Gale crater, Mars. *Journal of Geophysical Research: Planets* **125**, e2019JE006173 (2020).
184. A. C. McAdam *et al.*, Evolved gas analyses of sedimentary rocks from the Glen Torridon clay-bearing unit, Gale crater, Mars: Results from the Mars Science Laboratory Sample Analysis at Mars Instrument Suite. *Journal of Geophysical Research: Planets* **127**, e2022JE007179 (2022).
185. A. C. McAdam *et al.*, Constraints on mineralogy and geochemistry of Vera Rubin Ridge, Gale crater, Mars, from Mars Science Laboratory Sample Analysis at Mars evolved gas analyses. *Journal of Geophysical Research: Planets* **125**, e2019JE006309 (2020).
186. H. R. Westrich, Determination of water in volcanic glasses by Karl-Fischer titration. *Chemical Geology* **63**, 335–340 (1987).
187. F. Pineau, M. Javoy, Strong degassing at ridge crests: The behaviour of dissolved carbon and water in basalt glasses at 14°N, Mid-Atlantic Ridge. *Earth and Planetary Science Letters* **123**, 179–198 (1994).
188. M. T. Thorpe *et al.*, Mars Science Laboratory CheMin data from the Glen Torridon region and the significance of lake-groundwater interactions in interpreting mineralogy and sedimentary history. *Journal of Geophysical Research: Planets* (2022).
189. R. F. Weiss, B. A. Price, Nitrous oxide solubility in water and seawater. *Marine Chemistry* **8**, 347–359 (1980).
190. W. Wagner, A. Pruss, The IAPWS formulation 1995 for the thermodynamic properties of ordinary water substance for general and scientific use. *Journal of Physical and Chemical Reference Data* **31**, 387–535 (2002).
191. S. M. F. Sheppard, H. A. Gilg, Stable isotope geochemistry of clay minerals. *Clay Minerals* **31**, 1–24 (1996).
192. B.-L. Xu, Y.-F. Zheng, Experimental studies of oxygen and hydrogen isotope fractionations between precipitated brucite and water at low temperatures. *Geochimica et Cosmochimica Acta* **63**, 2009–2018 (1999).
193. C. J. Yapp, Oxygen isotopes in synthetic goethite and a model for the apparent pH dependence of goethite–water $^{18}O/^{16}O$ fractionation. *Geochimica et Cosmochimica Acta* **71**, 1115–1129 (2007).
194. R. O. Rye, R. E. Stoffregen, Jarosite-water oxygen and hydrogen isotope fractionations: Preliminary experimental data. *Economic Geology* **90**, 2336–2342 (1995).
195. W. Feng, C. J. Yapp, Experimental tests of the effects of Al substitution on the goethite–water D/H fractionation factor. *Geochimica et Cosmochimica Acta* **72**, 1295–1311 (2009).
196. C. N. Alpers, R. O. Rye, D. K. Nordstrom, L. D. White, B.-S. King, Chemical, crystallographic, and stable isotopic properties of alunite and jarosite from acid-hypersaline Australian lakes. *Chemical Geology* **96**, 203–226 (1992).

© 2025. All rights reserved.
